# Relict duck-billed dinosaurs survived into the last age of the dinosaurs in subantarctic Chile

**DOI:** 10.1126/sciadv.adg2456

**Published:** 2023-06-16

**Authors:** Jhonatan Alarcón-Muñoz, Alexander O. Vargas, Hans P. Püschel, Sergio Soto-Acuña, Leslie Manríquez, Marcelo Leppe, Jonatan Kaluza, Verónica Milla, Carolina S. Gutstein, José Palma-Liberona, Wolfgang Stinnesbeck, Eberhard Frey, Juan Pablo Pino, Dániel Bajor, Elaine Núñez, Héctor Ortiz, David Rubilar-Rogers, Penélope Cruzado-Caballero

**Affiliations:** ^1^ Red Paleontológica U-Chile, Departamento de Biología, Facultad de Ciencias, Universidad de Chile, Santiago, Chile.; ^2^ Departamento de Ciencias Ecológicas, Facultad de Ciencias, Universidad de Chile, Santiago, Chile.; ^3^ Área Paleontología, Museo Nacional de Historia Natural de Chile, Santiago, Chile.; ^4^ School of GeoSciences, University of Edinburgh, Grant Institute, Edinburgh, UK.; ^5^ KayTreng Consultores SpA, Ñuñoa, Santiago, Chile.; ^6^ Escuela de Geología, Facultad de Ciencias, Universidad Mayor, Manuel Montt 367, Providencia, Santiago, Chile.; ^7^ Universidade do Vale do Rio dos Sinos, São Leopoldo, Brazil.; ^8^ Laboratorio de Paleobiología, Instituto Nacional Antártico Chileno, Punta Arenas, Chile.; ^9^ Fundación Félix de Azara, Argentina, CONICET, Buenos Aires, Argentina.; ^10^ Universidad de Concepción, Concepción, Chile.; ^11^ Paleo Consultores, Pedro de Valdivia 273, Providencia 1602, Chile.; ^12^ Facultad de Ciencias Biológicas, Pontificia Universidad Católica de Chile, Santiago, Chile.; ^13^ Institut für Geowissenschaften, Ruprecht-Karls-Universität Heidelberg, Im Neuenheimer Feld 234-236, Heidelberg 69120, Germany.; ^14^ Staatliches Museum für Naturkunde Karlsruhe (SMNK), Erbprinzenstraße 13, Karlsruhe 76133, Germany.; ^15^ Universidad de Magallanes, Punta Arenas, Chile.; ^16^ Área de Paleontología, Departamento de Biología Animal, Edafología y Geología, Universidad de La Laguna, Tenerife, Spain.; ^17^ Grupo Aragosaurus-IUCA, Facultad de Ciencias, Universidad de Zaragoza, Zaragoza, Spain.

## Abstract

In the dusk of the Mesozoic, advanced duck-billed dinosaurs (Hadrosauridae) were so successful that they likely outcompeted other herbivores, contributing to declines in dinosaur diversity. From Laurasia, hadrosaurids dispersed widely, colonizing Africa, South America, and, allegedly, Antarctica. Here, we present the first species of a duck-billed dinosaur from a subantarctic region, *Gonkoken nanoi*, of early Maastrichtian age in Magallanes, Chile. Unlike duckbills further north in Patagonia, *Gonkoken* descends from North American forms diverging shortly before the origin of Hadrosauridae. However, at the time, non-hadrosaurids in North America had become replaced by hadrosaurids. We propose that the ancestors of *Gonkoken* arrived earlier in South America and reached further south, into regions where hadrosaurids never arrived: All alleged subantarctic and Antarctic remains of hadrosaurids could belong to non-hadrosaurid duckbills like *Gonkoken*. Dinosaur faunas of the world underwent qualitatively different changes before the Cretaceous-Paleogene asteroid impact, which should be considered when discussing their possible vulnerability.

## Teaser

Subantarctic fossils rewrite the biogeographic history of duck-billed dinosaurs and their role in pre-extinction ecosystems.

## INTRODUCTION

Duck-billed dinosaurs (Hadrosauroidea) are among the most successful herbivores in the history of life on Earth. Their tooth batteries with hundreds of teeth are arguably the most complex in vertebrate evolution (*1*) and were capable of crushing, grinding, and shearing, allowing them to exploit a broad range of plant resources that they could further reach at both short and high distances above ground (*2*). The duck-billed dinosaurs attained their maximum historical diversity in the dusk of the dinosaur age, upon radiation of the advanced forms of the family Hadrosauridae (the “true” duckbills) during the Campanian-Maastrichtian (*3*). Hadrosaurids became so successful that they are thought to have outcompeted other herbivores, in relation to evidence for declines of dinosaur diversity in North America and Central China (*2*). In addition, during the Campanian-Maastrichtian, hadrosaurids were perhaps the only dinosaurs from Laurasia to have successfully colonized Gondwanan continents (*4*). One species has been described from partial remains in Africa (*5*) and as many as five species have been named from central and northern Patagonia, where their remains are abundant (*4*, *6*–*9*). This opens interesting questions about the impact that hadrosaurids could have had on otherwise highly endemic dinosaur faunas of Gondwana and whether these faunas also experienced a decline in diversity previous to the asteroid impact of the Cretaceous-Paleogene (K-Pg).

Late Cretaceous faunas of continental vertebrates are poorly known in Antarctica and subantarctic regions. Here, we will use the term “subantarctic” in the physiographic sense, as it refers to the territories immediately north of the Antarctic convergence and roughly south 46°S (the southern limit of Chubut, central Patagonia). The arrival of duck-billed dinosaurs into southern Patagonia and the Antarctic Peninsula is documented by unnamed partial remains that are currently assumed to belong to hadrosaurids like those of central and northern Patagonia (*10*–*13*). At the time, southern Patagonia and the Antarctic Peninsula retained close geographic proximity to each other, with intermittent formation of land bridges and similar biotic components that have led to the suggestion of a distinct West Weddelian Terrestrial Biogeographic Province (*10*). These territories may have also become at least partially isolated from the rest of South America due to marine transgressions, such as the Kawas Sea (*14*).

**Fig. 1.**
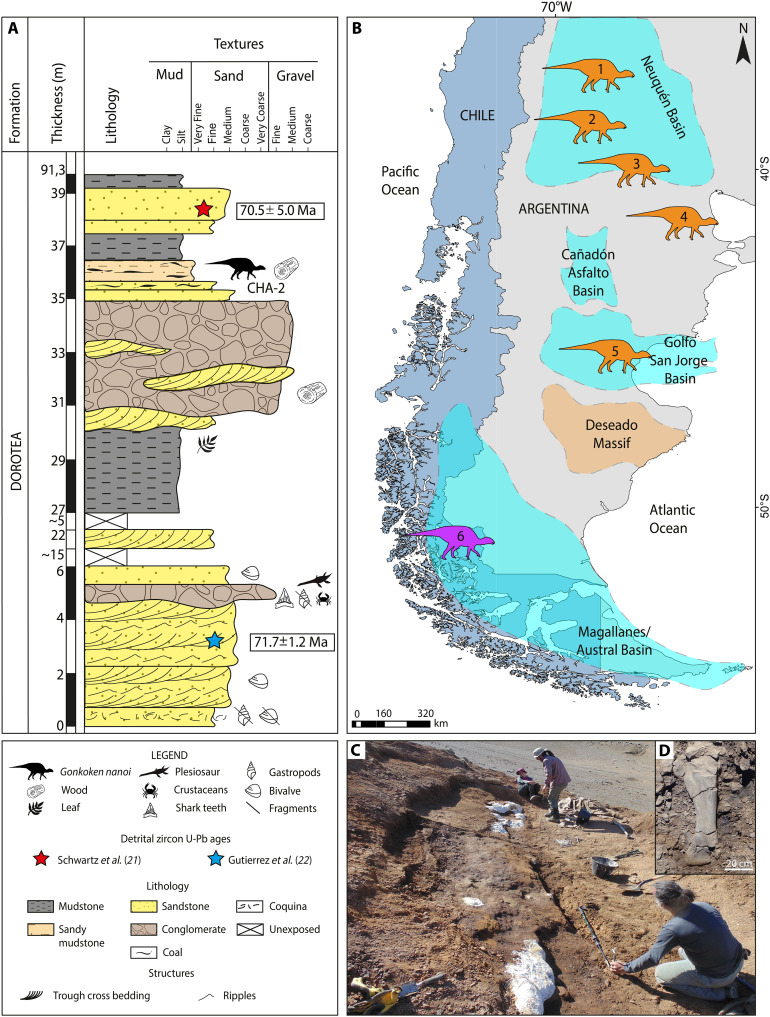
Stratigraphic location of *Gonkoken nanoi* and geographic distribution of South American duck-billed dinosaurs. (**A**) Stratigraphic section of the Dorotea Formation, indicating the location of the bones of *Gonkoken nanoi*. (**B**) Location of the duck-billed dinosaurs from South America: 1, *Lapampasaurus cholinoi* (Islas Malvinas locality, La Pampa Province, late Campanian to early Maastrichtian, Allen Formation); 2, *Kelumapusaura machi* (Matadero Hill, General Roca city, Río Negro Province, middle Campanian to early Maastrichtian, Allen Formation); 3, *Bonapartesaurus rionegrensis* (Salitral Moreno, General Roca Department, Río Negro Province, middle Campanian to early Maastrichtian, Allen Formation); 4, *Huallasaurus australis* (Arroyo Verde Puelén Department, Río Negro Province, late Campanian to early Maastrichtian, Los Alamitos Formation); 5, *Secernosaurus koerneri* (Río Chico, east of Lake Colhué Huapi, Chubut Province, Maastrichtian, Lago Colhué Huapi Formation); and 6, *Gonkoken nanoi* (Río de las Chinas Valley, Magallanes Region, late Campanian to early Maastrichtian, Dorotea Formation). (**C**) The quarry from which *Gonkoken nanoi* bones were excavated (bonebed level). (**D**) Detail of a nearly complete tibia.

In 2013, we initiated excavation of a monodominant bonebed with abundant disarticulated remains from several individuals of a subantarctic duck-billed dinosaur. The site is of early Maastrichtian age (see Materials and Methods) and is located in the Río de las Chinas valley of the Magallanes Region of subantarctic Chile, at the southernmost tip of southern Patagonia (51°S; Fig. 1). Here, we describe 45 skeletal elements from at least three different individuals (as inferred from three left humeri and three right femora) that were recovered from the excavation of about 28 m^2^ of a layer approximately 80 cm thick. The site shows no signs of running out of fossils, and excavations continue with many additional remains currently in preparation at the lab (fig. S1). Further outcrops with abundant duckbill bones are found in an extension of about 5 km (see Materials and Methods). The materials recovered so far are the most informative for any duck-billed dinosaur this far south and yield unexpected conclusions. Instead of being closely related to other South American duckbills, the dinosaur described here is not a hadrosaurid: Instead, it is similar to ancient Laurasian lineages that diverged shortly before the origin of Hadrosauridae (*15*). In technical terms, it is a non-hadrosaurid hadrosauroid that is closely related to Hadrosauridae. For the sake of simplicity, we will refer to it as a “transitional” duckbill, to emphasize that its morphology is transitional to that of the "true" (later diverging) duckbills of the family Hadrosauridae.

## RESULTS

### Systematic paleontology

  Dinosauria Owen, 1842

Ornithischia Seeley, 1887

Ornithopoda Marsh, 1881

Hadrosauroidea Cope, 1870, sensu Madzia *et al.* (*16*)

*Gonkoken nanoi* gen. et sp. nov.

### Holotype—CPAP 3054, right ilium

Paleontological Collection of Antarctica and Patagonia (CPAP) 3054 was found among several other disarticulated skeletal elements, precluding a confident assignment of multiple elements to specific individuals. We therefore designated CPAP 3054 as the holotype because of its evident diagnostic features. The preacetabular process of CPAP 3054 is incomplete but shows a “T”-shaped cross section due to the well-developed lateral and medial crests that characterize adults (*17*). These features allowed us to exclude a juvenile status [that is, an animal with no signs of impending maturity, (*18*)].

### Paratypes

**Fig. 2.**
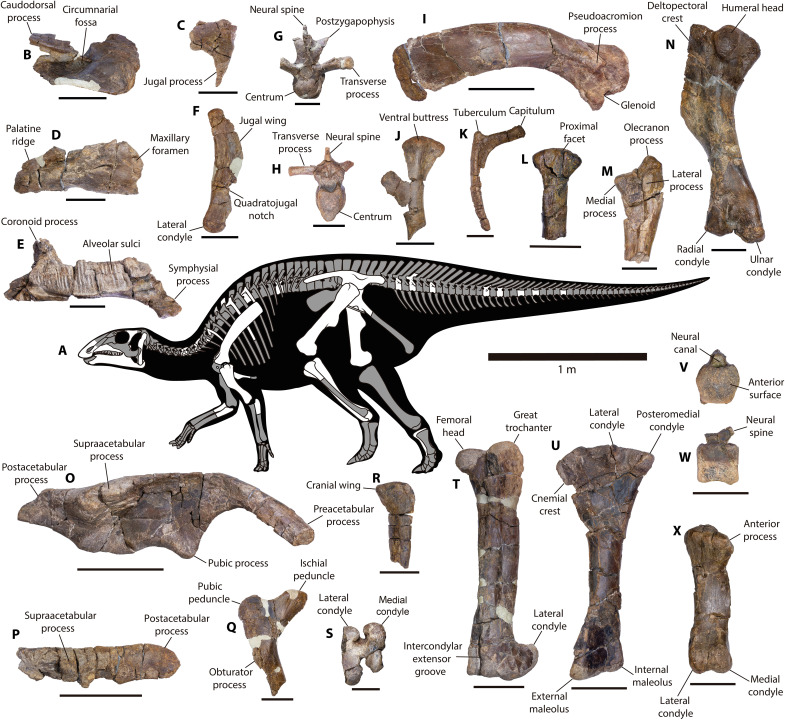
*Gonkoken nanoi* gen. et. sp. nov., skeletal anatomy. (**A**) Bones described in this work (white). Some elements are indicated specularly to facilitate their representation. (**B**) CPAP 5337, right premaxilla in lateral view. (**C**) CPAP 5341, incomplete left postorbital in lateral view. (**D**) CPAP 5340, incomplete right maxilla in lateral view. (**E**) CPAP 5370, left dentary in medial view. (**F**) CPAP 5343, right quadrate in lateral view. (**G**) CPAP 5344, cervical vertebra in anterior view. (**H**) CPAP 5346, dorsal vertebra in anterior view. (**I**) CPAP 5371, right scapula in lateral view. (**J**) CPAP 5352, left sternum in ventral view. (**K**) CPAP 5400, incomplete right rib in anterior view. (**L**) CPAP 5379, proximal portion of right radius in posterior view. (**M**) CPAP 5355, incomplete left ulna in anterolateral view. (**N**) CPAP 5353, left humerus in posterolateral view. (**O**) CPAP 3054 (holotype), right ilium in lateral view. (**P**) CPAP 5356, left postacetabular process in lateral view. (**Q**) CPAP 5357, proximal portion of right ischium in medial view. (**R**) CPAP 5363, proximal portion of left fibula in lateral view. (**S**) CPAP 5360, incomplete right femur in distal view. (**T**) CPAP 5358, left femur in anterior view. (**U**) CPAP 5362, left tibia in lateral view. (**V** and **W**) CPAP 5349, caudal vertebra in anterior and lateral views. (**X**) CPAP 5364, right metatarsal III in anterior view. Skeleton modified from (*31*). Scale bars, 50 mm [(B) to (G), (J), (K) to (N), (Q), (S), and (V) to (X)] and 100 mm [(H), (I), (O), (P), (R), (T), and (U)].

Several disarticulated bones were selected as paratypes of the same species (Fig. 2 and figs. S2 to S4). This is based on extensive overlapping elements from different individuals, without any evidence for more than one species at the site (see the Supplementary Materials). Taphonomical aspects at the site such as hydraulic equivalence and lack of abrasion also suggest that bones underwent minimal transport (see the Taphonomy section in the Supplementary Materials and tables S1 and S2) and do not point to any clear nonbiological reason (such as a river bend) for the accumulated remains of numerous individuals. Instead, social behavior such as herding is often considered to explain monodominant accumulations of duck-billed dinosaurs, which are common in the fossil record (*19*). The bones assigned as paratypes are the following: a right premaxilla (CPAP 5337), the incomplete left maxilla of a juvenile individual (CPAP 5338), an incomplete left maxilla (CPAP 5339), an incomplete right maxilla (CPAP 5340), an incomplete left postorbital (CPAP 5341), an incomplete right dentary (CPAP 5342), a left dentary (CPAP 5370), a fragmentary right dentary (CPAP 5368), a right quadrate (CPAP 5343), a cervical vertebra (CPAP 5344), a mid-cervical centrum (CPAP 5380), two incomplete dorsal vertebrae (CPAP 5345 and CPAP 5346), a dorsal neural arch (CPAP 5396), eight proximal and mid caudal vertebrae (CPAP 5347, CPAP 5348, CPAP 5349, CPAP 5350, CPAP 5351, CPAP 5397, CPAP 5398, and CPAP 5399), four incomplete ribs (CPAP 5400, CPAP 5401, CPAP 5402, and CPAP 5403), a right scapula (CPAP 5371), an incomplete left sternum (CPAP 5352), a complete left humerus (CPAP 5353), an incomplete left humerus (CPAP 5354), two fragments of a left humerus (CPAP 5369), an incomplete left ulna (CPAP 5355), an incomplete right ulna (CPAP 5392), an incomplete right radius (CPAP 5379), a left postacetabular process with part of the iliac blade (CPAP 5356), an incomplete right ischium (CPAP 5357), a complete left femur (CPAP 5358), an incomplete right femur (CPAP 5359), the distal end of a right femur (CPAP 5360), the distal end of the right femur of a juvenile individual (CPAP 5361), a complete left tibia (CPAP 5362), an incomplete left tibia (CPAP 5372), an incomplete left fibula (CPAP 5363), and a right metatarsal III (CPAP 5364).

### Etymology

The words “gon” (same as, similar to) and “koken” (wild duck or swan) are in the language of the Aónik’enk, the indigenous people that inhabited the region where this species was found (*20*). The specific name nanoi is in honor of Mario “Nano” Ulloa, who first found dinosaur bones at Río de las Chinas Valley and provided key logistic help during our expeditions.

### Type locality and horizon

Loma Koken (50°42′42″S and 72°32′29″W), El Puesto area, Río de las Chinas Valley, Estancia Cerro Guido, Magallanes Region, Chilean Patagonia (51°S). Upper section of the Dorotea Formation (lower Maastrichtian), between 71.7 ± 1.2 and 70.5 ± 5.0 million years (Ma) (*21*, *22*) (see Materials and Methods).

### Diagnosis

Small-sized hadrosauroid dinosaur (total body length ~4 m) presenting the following autapomorphies: a scapula with an anteroventrally curved pseudoacromion process and an ilium with a medioventrally and anteroposteriorly well-developed sacral crest extending posterior to the base of the postacetabular process. *Gonkoken* also differs from all other members of Hadrosauroidea in its unique combination of characters. Unlike the Hadrosauridae, it shows a maxilla with a jugal articular surface showing a prominent and caudally projecting dorsal tubercle; a dentary with a short diastema, mandibular symphysis oblique relative to its long axis, and tooth rows with less than 30 tooth positions that do not extend beyond the coronoid process and converge anteriorly with the lateral surface of the dentary; quadrate with a medial condyle that is not markedly elevated dorsally compared with the lateral condyle; a deltopectoral crest with a rounded laterodistal end extending less than 48% of the total length of the humerus; ilium with a nearly straight dorsal border and a supraacetabular process longer than 70% of the length of the iliac blade; and tibia with a cnemial crest extending less than 50% of the total length of the bone. In addition to these characters, *Gonkoken* differs specifically from South American Hadrosauridae by a scapula with a mediolaterally narrow coracoidal facet and a more ventrally curved pseudoacromion process, and an ilium with a more laterally curved preacetabular process, a less laterally developed supraacetabular process, and a shorter and ventrally oriented pubic peduncle. *Gonkoken* differs from early-diverging Hadrosauroidea in that it presents derived characters also found in Hadrosauridae: a premaxilla with a “double-layer” of denticles; a supraacetabular process anteriorly located with respect to the dorsal tuberculum of the ischial peduncle; the ratio between the base of the preacetabular process and the distance between the dorsal border of the ilium and the pubic peduncle is greater than 0.5; and a posteromedial condyle of the proximal end of the tibia that is more robust than the lateral one. Regarding non-hadrosaurids that are closely related to Hadrosauridae, *Gonkoken* differs from *Eotrachodon* in that the precircumnarial region of the premaxilla is more anteroposteriorly extended, lacking a caudally everted oral margin, and the maxilla has an articular surface for the jugal located anterior to the dorsal process, rather than posterior to it. *Gonkoken* differs from *Lophorhothon* in that the jugal process of the postorbital is pointing ventrally and the posterior edge of this process is slightly convex, rather than concave, and the ilium is higher, with a laterally less developed supraacetabular process, located anterior to the dorsal tubercle of the ischial peduncle. *Gonkoken* differs from *Huehuecanauhtlus* in that the preacetabular process of the ilium in dorsal view is markedly curved laterally, less curved ventrally, and its base is dorsoventrally narrower; the dorsal tubercle of the ischial peduncle of the ilium is less dorsally elevated; and the postacetabular process is approximately rectangular in lateral view, rather than triangular.

### Diagnosis of the genus

As for the type species.

### Description

**Fig. 3.**
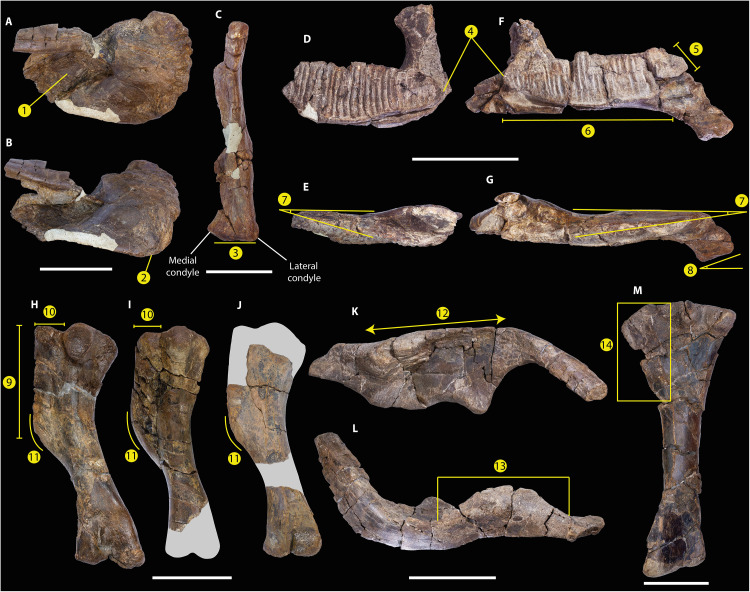
*Gonkoken nanoi* lacks several derived characters found in Hadrosauridae. A selection of plesiomorphic characters is shown. (**A** and **B**) CPAP 5337, right premaxilla in dorsal (A) and lateral (B) views showing a circumnarial fossa without accessory fossa or foramina (1) and an anteroventrally deflected oral margin (2). (**C**) CPAP 5343, right quadrate in posterior view. The lateral condyle is not markedly offset ventrally with respect to the medial condyle, unlike Hadrosauridae (3). CPAP 5342, right dentary in medial (**D**) and dorsal (**E**) views, and CPAP 5370, left dentary in medial (**F**) and dorsal (**G**) views. Tooth rows do not exceed the coronoid process (4), the diastema is short (5), there are less than 30 dental positions (6), tooth row converges anteriorly with the lateral surface of the dentary (7), and the symphysis is oblique (8). CPAP 5353 (**H**), CPAP 5354 (**I**), and CPAP 5369 (**J**), left humeri in posterolateral view, in which the ratio between length of the deltopectoral crest and the total length of the humerus is lower than 0.48 (9), and the deltopectoral crest is mediolaterally short (10), with a widely arcuate laterodistal corner (11). (**K** and **L**) CPAP 3054, right ilium in lateral (K) and dorsal (L) views. The dorsal border of the ilium is almost straight (12), and the length of the supraacetabular process is higher than 70% of the length of the iliac blade (13). (**M**) CPAP 5362, left tibia in lateral view. In *Gonkoken*, the length of the cnemial crest is lower than the 50% of the total length of the bone (14). Scale bars, 50 mm (A) to (C) and 100 mm (D) to (M).

**Fig. 4.**
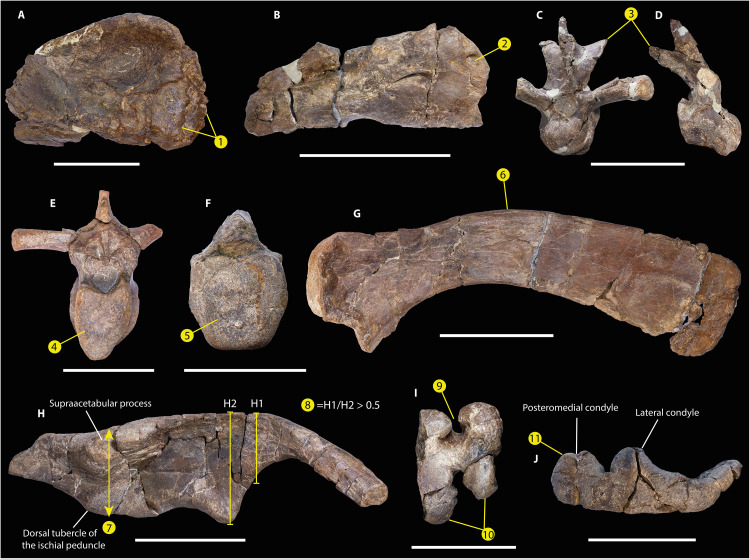
Derived characters of *Gonkoken nanoi* shared with Hadrosauridae. *G. nanoi* supports these characters evolved before the origin of this family. (**A**) CPAP 5337, right premaxilla in ventral view, which shows a double-layer morphology (1). (**B**) CPAP 5340, right maxilla in lateral view, with a rostral maxillary foramen on the lateral surface of the bone (2). (**C** and **D**) CPAP 5344, mid-cervical vertebra in anterior (C) and lateral view (D). This cervical vertebra has elevated and well-developed postzygapophyseal processes, which are dorsally arched (3). (**E**) CPAP 5346, dorsal vertebra whose centrum has a “heart-shaped” articular surface (4). (**F**) CPAP 5347, mid-caudal vertebra with a hexagonal articular surface (5). (**G**) CPAP 5371, right scapula in medial view, which is dorsally arched (6). (**H**) CPAP 3054, right ilium in lateral view. The supraacetabular process is anteriorly located with respect to the dorsal tuberculum of the ischial peduncle (7). Furthermore, the ratio between the base of the preacetabular process (H1) and the distance between the dorsal border of the ilium and the pubic peduncle (H2) is greater than 0.5 (8). (**I**) CPAP 5360, distal end of the right femur in distal view, showing a deep intercondylar extensor groove, in which the condyles nearly meet anteriorly (9). In addition, the condyles are posteriorly well-developed (10). (**J**) CPAP 5362, left tibia in proximal view in which the posteromedial condyle is wider than the lateral condyle (11). Scale bars, 50 mm (A) and (F) and 100 mm [(B) to (E) and (G) to (J)].

The premaxilla has an oval, very deep circumnarial fossa that lacks accessory fossae, foramina, and ridges, unlike Saurolophinae [Fig. 3; (*23*–*27*)]. The oral margin is convex and anteroventrally deflected, unlike most Saurolophinae [Fig. 3; (*28*)]. It shows a denticulate margin, separated by a shallow groove from the denticulate border of the palatine (“double layer”), as in saurolophines, lambeosaurines, and some non-hadrosaurid hadrosauroids [Fig. 4; (*23*, *27*, *29*–*32*)]. The maxilla is subtriangular in lateral view. The articular surface for the jugal is robust and subquadrangular in shape and has a prominent dorsal tubercle, unlike hadrosaurids (*33*, *34*). A large rostral maxillary foramen is present at the lateral side, as in Saurolophinae [Fig. 4; (*23*, *29*)]. The medial surface bears anteroposteriorly aligned neurovascular foramina (*23*, *29*). The teeth are poorly preserved, making it difficult to determine their morphology and number in the maxilla. The postorbital is T-shaped in lateral view. Unlike most hadrosaurids, the dentary has a short diastema (less than 20% of the length of the alveolar row), and the mandibular symphysis is oblique relative to the long axis of the dentary, as in several non-hadrosaurid duckbills [Fig. 3; (*26*, *35*, *36*)]. The alveolar furrows are parallel to each other and dorsoventrally extended. The most complete dentary (CPAP 5370) shows about 25 tooth positions, rather than more than 30 as in Hadrosauridae [Fig. 3; (*28*, *29*, *37*, *38*)]. The dentary tooth row extends further posteriorly than in basal iguanodontians, reaching the coronoid process, but not extending beyond it, unlike hadrosaurids [Fig. 3; (*29*, *34*, *38*)]. The tooth row converges anteriorly with the lateral surface of the dentary, unlike *Plesiohadros* and Hadrosauridae [Fig. 3; (*27*, *29*, *39*, *40*)]. The coronoid process is robust, almost straight, and separated from the alveolar row by a groove.

The quadrate is dorsoventrally elongated. Its dorsal half curves slightly posteriorly and lacks a squamosal buttress. The quadratojugal notch is ventrally positioned. Unlike most hadrosaurids, the lateral condyle is not markedly offset ventrally with respect to the medial condyle [Fig. 3; (*29*, *35*)]. The cervical centrum is longer than dorsoventrally high and strongly opisthocoelous. The anterior and posterior articular surfaces are oval. The postzygapophyses are well elevated dorsally to the roof of the neural canal and curved posteriorly and laterally, which is common in hadrosaurids [Fig. 4; (*23*)]. The dorsal centra are elongated, higher than wide, and opisthocoelous, and have heart-shaped articular faces like hadrosaurids and at least some non-hadrosaurid hadrosauroids [Fig. 4; (*23*, *25*, *31*)]. The caudal centra are amphiplatyan with hexagonal articular contours, which is typical but not exclusive to hadrosaurids [Fig. 4; (*23*, *25*, *28*)].

The sternum is hatchet-shaped. The scapula has a dorsally curved blade as is typical of hadrosaurids [Fig. 4; (*29*)], with a rectangular posterior end. The deltoid ridge is poorly developed. The glenoid fossa narrows mediolaterally, and the pseudoacromion process is prominent and anteroventrally curved, unlike in all other South American duckbills (fig. S5). In addition, unlike South American hadrosaurids such as *Huallasaurus*, *Lapampasaurus*, and *Kelumapusaura*, the coracoidal facet is mediolaterally narrower (fig. S5).

Unlike Hadrosauridae, the deltopectoral crest of the humerus extends less than 48% the total length of the bone (*29*, *41*). The ratio between length and maximum width of the deltopectoral crest is 3.9, which is higher than in Saurolophidae (*17*). The laterodistal corner of the deltopectoral crest is widely arcuate, unlike most hadrosaurids [Fig. 3; (*29*, *41*, *42*)]. The ulna has a prominent olecranon process. The iliac peduncle of the ischium is longer than the pubic peduncle. The dorsal border of the ilium is almost straight, unlike *Huehuecanauhtlus* and hadrosaurids, including South American duckbills [fig. S6 and (*25*, *26*, *28*, *41*, *43*)]. The sacral crest is prominent and more developed than in other South American duckbills (fig. S6). The preacetabular process is incomplete. This is robust, with a T-shaped cross section, and is more laterally curved than in other South American duckbills (fig. S6). The preserved anterior end of the preacetabular process surpasses the lateral extension of the supraacetabular process, as in *Secernosaurus*, but not in other South American duckbills (fig. S6). The ratio between the maximum height of the caudal end of the preacetabular process and the distance between the dorsal margin of the ilium and the ventral edge of the pubic peduncle is 0.58, which is higher than in non-hadrosaurid hadrosauroids [Fig. 4; (*9*, *29*)]. The pubic peduncle is robust and triangular in lateral view, unlike the elongated, rod-like pubic peduncle of basal iguanodontoids such as *Iguanodon* (*44*). The pubic peduncle is ventrally oriented and shorter than in some South American duckbills (fig. S6). The ischial peduncle consists of two low and blunt tubercles of similar size. The supraacetabular process projects slightly lateroventrally, with its ventral apex slightly anteriorly located with respect to the dorsal tubercle of the ischial peduncle, as in Hadrosauridae [Fig. 4; (*17*, *41*)]. The length of the supraacetabular process reaches approximately 82.6% of the length of the central plate of the ilium. This is within the range of non-hadrosaurid duckbills: lower than in basal iguanodontoids such as *Iguanodon* (>85%) and higher than in Hadrosauridae (<70%) [Fig. 3; (*41*)]. The lateroventral protrusion of the supraacetabular process is less marked than in other South American duckbills except for *Secernosaurus* (fig. S6). The postacetabular process is rectangular, slightly dorsally inclined, and twisted. The femur is straight, with a triangular fourth trochanter. The flexor and extensor grooves are deep, with the latter almost closed, and the distal condyles are strongly projected posteriorly, which are features typical of hadrosaurids [Fig. 4; (*23*)]. The lesser trochanter is small and well-separated from the greater trochanter. The cnemial crest extends to less than half the total length of the bone, which differs from all hadrosaurids except *Bonapartesaurus* [Fig. 3; (*9*, *35*, *45*)]. Posterior to the cnemial crest, there are two rounded condyles separated by a sulcus. The posteromedial condyle is more robust and longer than the lateral condyle, like Hadrosauridae [Fig. 4; (*46*)]. The tibia has a torsion of approximately 45° between the proximal and distal epiphysis. The external malleolus is more extended distally than the internal one. A fragment of a fibula reveals an enlarged proximal end. As in some non-hadrosaurid duckbills such as *Bactrosaurus*, the articular surface of the metatarsal III is triangular (*25*); the ratio between the length and mediolateral width of this bone is 4.5.

## DISCUSSION

**Fig. 5.**
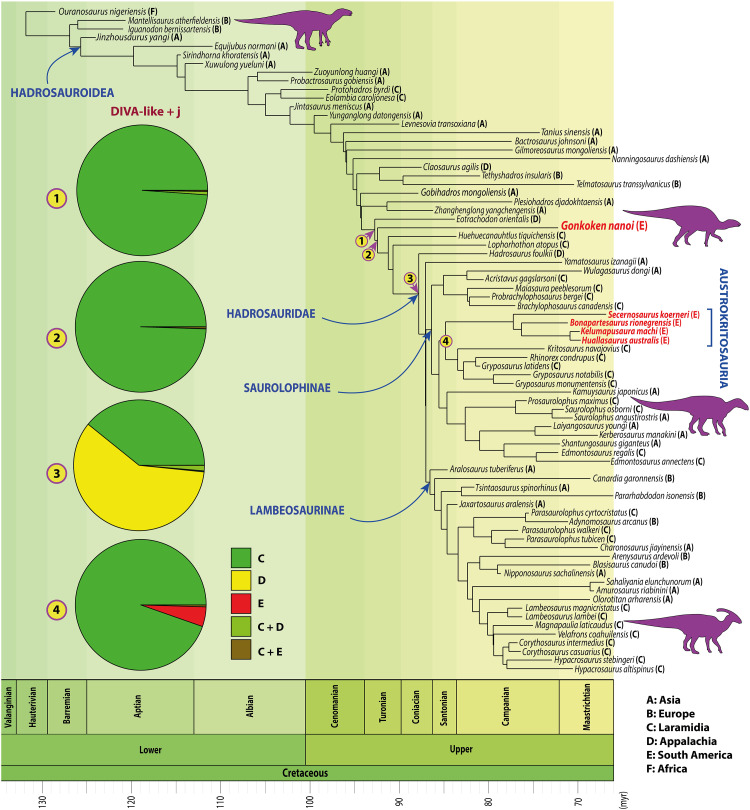
Phylogenetic relationships. Parsimony and Bayesian analyses recovered *Gonkoken* at a position transitional to Hadrosauridae, diverging shortly before the origin of this family; results of Parsimony are shown. The DIVA-like + j model (BioGeoBEARS) recovers Laramidia as the ancestral area for the most recent common ancestor of *Eotrachodon* and Hadrosauridae (node 1), as well as for the most recent common ancestor of *Gonkoken* and Hadrosauridae (node 2). Appalachia or Laramidia are recovered as ancestral areas for Hadrosauridae (node 3) and Laramidia as the ancestral area for Kritosaurini +Austrokritosauria (node 4). The names of the South American duck-billed dinosaurs are highlighted in red.

*Gonkoken* is a duck-billed dinosaur that shares some derived traits with true duckbills of the family Hadrosauridae (Fig. 4), while several other characters retain the ancestral condition for Hadrosauroidea (Fig. 3). This transitional morphology suggests that *Gonkoken* is not a hadrosaurid but is nevertheless closely related to Hadrosauridae, having diverged shortly before the origin of this family. To properly address the relationships of *Gonkoken*, we carried out the most comprehensive phylogenetic analysis of duck-billed dinosaurs to date, using a data matrix from a recent study (*4*), which we modified to correct scorings and include more characters and more taxa such as *Gobihadros* and *Huehuecanauhtlus*, which are important forms transitional to Hadrosauridae [(*31*, *43*); see Materials and Methods]. The South American duckbill *Lapampasaurus* was excluded because it is too fragmentary to assess its affinities (keeping this taxon also had no effect; see Materials and Methods). The results for our maximum parsimony analysis were unprecedentedly well-resolved, obtaining only four most parsimonious trees [1335 steps, Consistency Index (CI): 0.390; Retention Index (RI): 0.816]; further, all differences among these trees were in early-branching taxa at a node distant from *Gonkoken* (fig. S7 shows the strict consensus of all four trees). Parsimony places *Gonkoken* outside of Hadrosauridae but among transitional duckbills that are closest to this family (Fig. 5 and fig. S7; see Materials and Methods for selection and time calibration of this tree). Tip-dated Bayesian analyses also place *Gonkoken* as a close relative of Hadrosauridae, although its relations to other transitional duckbills were less resolved (figs. S8 and S9). Resolution improved in the undated Bayesian analysis, which coincides with parsimony in that *Gonkoken* and the North American taxa *Lophorhothon* and *Huehuecanauhtlus* are closer to Hadrosauridae than other transitional duckbills (fig. S8). Thus, these three taxa are likely most relevant to understanding the origins of Hadrosauridae. However, *Lophorhothon* and *Huehuecanauhtlus* are represented by very partial materials (*32*, *43*). *Gonkoken* provides more information, which is also bound to increase upon future excavations at the monotypic bonebed.

*Gonkoken* is not particularly related to other South American duckbills, which are advanced forms of the family Hadrosauridae. Recent work has proposed that all other South American duckbills are saurolophine hadrosaurids, forming a monophyletic group that is the sister to the Kritosaurini, a tribe that inhabited Laramidia in North America (*4*). The monophyly of these South American hadrosaurids suggests that all members of this group descend from a single species that dispersed into South America. In our own analysis, despite inclusion of *Gonkoken* and other taxa, and the recoding of numerous characters, we recovered the same monophyletic group of South American saurolophine hadrosaurids in both Parsimony and Bayesian analyses, which is further resolved by parsimony as the sister to Kritosaurini (Fig. 5 and figs. S7 to S9). Although these South American hadrosaurids are related to Kritosaurini, they cannot be referred to this tribe: Both parsimony and Bayesian analyses do not support a nested position within the North American forms that are used to define Kritosaurini (*47*). Thus, the current definition of Kritosaurini excludes these South American relatives. Given the need for a different and succinct term to refer to the monophyletic group of “South American saurolophine hadrosaurids closely related to Kritosaurini,” we propose the name Austrokritosauria for the most inclusive clade that contains *Huallasaurus* but not *Gryposaurus*. *Gonkoken* does not belong to this clade: placing *Gonkoken* as sister of Austrokritosauria requires a steep 15 extra steps in the parsimony analysis (table S3). Therefore, *Gonkoken* represents a completely different lineage of duck-billed dinosaurs that independently colonized South America.

As the most informative duck-billed dinosaur from far southern regions, *Gonkoken* also prescribes a reinterpretation of the record of partial duckbill fossils found in Southern Patagonia and Antarctica. Partial remains in these regions can no longer be assumed a priori to belong to hadrosaurids like those of central and northern Patagonia: none of these remains show characters that are exclusive to Hadrosauridae, especially when we take into account the hadrosaurid-like features of *Gonkoken*. In particular, two incomplete caudal centra from deposits of the Chorrillos Formation (Santa Cruz Province, Argentina) have been attributed to Hadrosauridae based on the hexagonal contour of their articular surfaces (*11*), but this trait is also present in *Gonkoken* (Fig. 4). An isolated and incomplete tooth from the Maastrichtian of Vega Island in the Antarctic Peninsula was attributed to Hadrosauridae and tentatively to Hadrosaurinae (=Saurolophinae) because of its relatively symmetrical crown, with a single and nearly straight medial keel, and poorly developed denticles (*13*). Although teeth are unknown in most non-hadrosaurid duckbills, including *Gonkoken*, these characters are documented in forms transitional to Hadrosauridae as in the maxillary teeth of *Eotrachodon* (*34*) and also in earlier lineages, as in the dentary teeth of *Tethyshadros* (*48*) and the maxillary teeth of *Eolambia* (*28*). Also from the Antarctic Peninsula, a metatarsal fragment found in Maastrichtian rocks of Seymour Island was attributed to Hadrosauridae (*12*), but its morphology has already been pointed out to be shared by other ornithopods, including those that inhabited austral regions such as the Elasmaria (*49*) and, now, non-hadrosaurid duckbills. In synthesis, hadrosaurids may have never reached into the Magallanes-Austral basin or further south, where no remains can be reliably attributed to Hadrosauridae. This may add to other faunistic differences that have been noticed between the fossil record of southern and northern Patagonia (*50*).

**Fig. 6.**
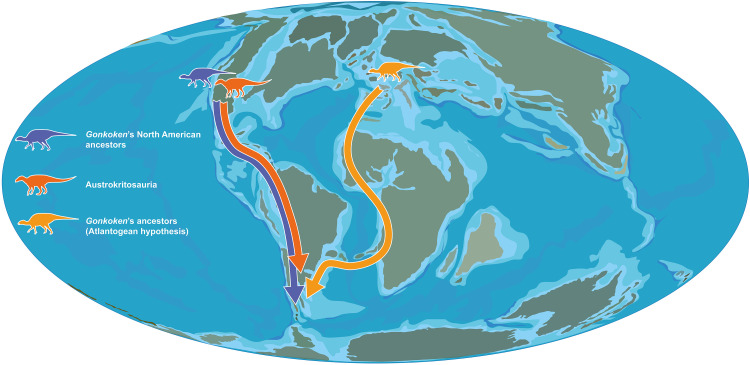
Biogeographic history of South American duck-bill dinosaurs. Biogeographic statistics suggest that *Gonkoken*’s ancestors arrived from North America (in purple). The alternative hypothesis that *Gonkoken’*s ancestors could have dispersed from Europe into South America via Africa (Atlantogean hypothesis, in orange) was not supported by biogeographic statistics. Hadrosaurids related to the tribe Kritosaurini (in red) would also have dispersed from North America, giving rise to the clade Austrokritosauria in South America. These hadrosaurids may have never reached the same high latitudes as *Gonkoken*’s ancestors, possibly because they arrived later into South America. Paleogeographic drawing is based on information from the PALEOMAP project of C. Scotese and (*10*).

*Gonkoken* is the first non-hadrosaurid duck-billed dinosaur ever found in Gondwana, so its presence so far south poses a challenging biogeographic enigma. Any explanation implies remarkably long routes, large gaps in between with no records of non-hadrosaurid duckbills, and marine barriers that stopped most Laurasian dinosaurs. Most likely, these marine barriers could have been breached through chains of islands rather than continuous land bridges (*51*), such that any dispersal of terrestrial animals must have involved swimming or “rafting” on debris (Fig. 6). The First American Biotic Exchange between North America and South America (*52*, *53*) is proposed to have initiated during the latest Cretaceous (Campanian-Maastrichtian) (*52*). Mammals of North American origin had already become diverse in South America in the early Paleocene, suggesting that they must have crossed over earlier: However, no remains have yet been found in the Cretaceous of South America (*53*, *54*). Currently, the most reliable fossil evidence of exchanges during the Cretaceous is provided by the Austrokritosauria, with other evidence also provided by *Patagopelta*, a possible nodosaurid from Patagonia (*4*, *55*), and the large North American titanosaur *Alamosaurus*, with potential ancestry in South America (*56*, *57*). Therefore, if the ancestors of *Gonkoken* were from North America, they could have followed a similar route. A radically different possibility is that the ancestors of *Gonkoken* were European, dispersing into Africa and from there into South America [see Fig. 6; the “Atlantogean” route (*57*)]. This is a longer route that also implies crossing two marine barriers (rather than one), although possible exchanges through this route have been argued to be better supported than those between the Americas, based on numerous shared biotic components between Europe and South America during the latest Cretaceous (*57*, *58*). Whichever the case, duck-billed dinosaurs had the highest vagility (dispersal capacity) documented for any dinosaurs, with the greatest number of dispersal events that likely crossed marine barriers, namely, between Asia and Laramidia, Appalachia and Laramidia (twice), Europe and Appalachia, Asia and Europe (twice), Europe and North Africa, Laramidia and South America, and South America and Antarctica (*5*). Duck-billed dinosaurs are also most often preserved near or within coastal environments [also the case for *Gonkoken*; see Materials and Methods; (*23*)] and have been suggested to be apt swimmers, or even semiaquatic (*5*).

To formally test hypotheses on the biogeographical history of *Gonkoken*, we reconstructed likely ancestral areas for all internal nodes of the phylogeny obtained from parsimony analysis, using BioGeoBEARS (*59*) as well as statistical Dispersal-Vicariance Analysis (s-DIVA) (*60*–*62*). The best-fit model in BioGeoBEARS was the DIVALIKE + j model (AICc: 200.80), which supports a strong role for events with founder effects [table S4 and (*5*)]. This model provides clear-cut support for a Laramidian origin for the last common ancestor shared by *Gonkoken* and Hadrosauridae, as well as for the last ancestor shared with *Eotrachodon*, at the previous node (Fig. 5 and fig. S11). The results of s-DIVA also support a North American origin, despite some differences, and decreased resolution: the last common ancestor shared by *Gonkoken* with Hadrosauridae is inferred in broad areas that combine Laramidia and/or Appalachia with South America, and the previous last ancestor shared with *Eotrachodon* is inferred as Appalachian (fig. S12). As for Austrokritosauria, the results of both s-DIVA and BioGeoBEARS support a Laramidian origin. This is the first time that formal biogeographical statistical analysis is shown to support the occurrence of the first American biotic exchange during the Cretaceous, for both *Gonkoken* and Austrokritosauria; similar studies may provide formal support for the dispersal of North American mammals and nodosaurid ankylosaurs into South America, and of titanosaurian sauropods from South America into North America.

Although the results of Biogeographic statistics did not support an Atlantogean route (from Europe via Africa) for the ancestors of *Gonkoken*, this could be a result of a sampling bias against latest Cretaceous faunas in Africa, which are poorly known (*5*, *63*, *64*). Non-hadrosaurid duckbills like *Tethyshadros* were present in Europe in the early Campanian (*26*, *40*, *48*), and later in the Maastrichtian, Lambeosaurine hadrosaurids managed to disperse from Europe into Africa despite the fact that these continents had drifted further apart (*5*). This has led to the suggestion that non-hadrosaurid duckbills could have well done the same before that, during the Cenomanian-Campanian (*48*). To explore this possibility, we added a hypothetical African taxon of non-hadrosaurid duckbill to our BioGeoBEARS and s-DIVA analyses, placing it between *Gonkoken* and *Eotrachodon*, or as sister to *Gonkoken*. A North American origin continued to be best supported, suggesting that this conclusion will not be easily overturned, even if such a discovery was made in Africa (figs. S13 to S16 and tables S5 and S6, see Materials and Methods and the Supplementary Materials for details).

Some dinosaur clades that are present in both North and South America have been shown to have ancient cosmopolitan origins before the final separation of Gondwana and Laurasia (*65*, *66*). Although *Gonkoken* is not a hadrosaurid, such a scenario would require for a much earlier divergence near the origin of duck-billed dinosaurs (Hadrosauroidea) in the late Hauterivian (130 Ma), when biotic exchanges between Europe and Gondwana were still possible (*57*). Such a basalmost position for *Gonkoken* would require a steep 46 extra steps (table S3). Given the actual phylogenetic position of *Gonkoken*, to insist on ancient cosmopolitan roots would imply that duckbills close to Hadrosauridae had already originated in the Hauterivian, despite a gap of about 45 Ma in their worldwide fossil record (Fig. 5).

We conclude that *Gonkoken* is very likely descended from North American non-hadrosaurids that are transitional to Hadrosauridae, the likes of *Eotrachodon*, *Lophorhothon*, and *Huehuecanauhtlus*. These were also the last non-hadrosaurids of North America, where they became replaced by hadrosaurids: *Eotrachodon* and *Huehuecanauhtlus* are of Santonian age; only *Lophorhothon* may have lived in the Campanian, given its large chronostratigraphic uncertainty, ranging from the latest Santonian (83.6 Ma) into the late Campanian (75.8 Ma) (*24*, *32*, *43*). Non-hadrosaurid duckbills are absent in the abundant and well-studied record of North American dinosaurs of the latest Campanian and Maastrichtian, strongly suggesting that they had become locally extinct (*30*, *67*). However, at some point before that, the non-hadrosaurid ancestors of *Gonkoken* managed to leave North America, surviving into the Maastrichtian as distant relict populations in Subantarctic Chile.

Given that *Gonkoken* is found so far south and that hadrosaurid remains cannot be confirmed from Subantarctic and Antarctic regions, this pattern suggests the ancestors of *Gonkoken* arrived earlier into South America than the hadrosaurids, giving the former a head start in reaching more Austral regions. Hadrosaurids in turn may have not had enough time to reach this far south before the K-Pg mass extinction. This interpretation is allowed by the divergence times in our time-calibrated tree: *Gonkoken* would have last shared an ancestor with North American forms around 91 Ma ago (Turonian), while the Austrokritosauria would have last shared an ancestor with the North American Kritosaurini around 85 Ma ago (Santonian; see Materials and Methods for calibration details). The discovery of *Gonkoken* provides empirical support to previous suggestions that the dispersion of duck-billed dinosaurs within South America could have followed such a phylogenetic-latitudinal pattern (*68*).

Another reason to suspect that hadrosaurids may have never arrived into Subantarctic and Antarctic regions is that they tended to replace non-hadrosaurids in those regions where they coexisted. The niches of hadrosaurids likely overlapped onto those of non-hadrosaurid duckbills that were often smaller-sized and had smaller tooth plates with fewer tooth positions, taking up a smaller proportion of the jaw (*28*, *29*, *37*, *38*). Given the importance of the tooth-jaw apparatus, this could explain why in the Maastrichtian non-hadrosaurid duckbills had disappeared in North America (*39*, *85*), while a single species is known from Asia (*69*). Non-hadrosaurid duckbills like *Telmatosaurus* persisted into the Maastrichtian in Europe, but this is argued to have resulted from their geographic isolation in islands (*26*, *40*). This is also a feasible explanation for *Gonkoken*, given evidence of marine transgression events and an archipelago at the time in southern South America.

The radiation and intercontinental dispersal of duck-billed dinosaurs are especially interesting given recent arguments that dinosaur faunas may have become vulnerable to extinction shortly before the K-Pg asteroid impact. Declines in the diversity of large-bodied, herbivorous ornithischians in North America (*70*) seem consistent with the possibility that hadrosaurids were outcompeting other herbivores. Duck-billed dinosaurs may have had an especially strong impact on the dinosaur faunas they encountered in Gondwana, which had evolved for a long time in isolation from Laurasia. However, pre-extinction declines in dinosaur diversity have been disputed on the grounds of potential geological and sampling biases, including the fact that they are purely based on records within Laurasia and may not represent a global phenomenon (*71*). No in-depth studies have yet been carried out in Gondwana that may support similar declines, and data collection in most regions (including Africa, Australia, New Zealand, Antarctica, and subantarctic South America) is still in the stage of obtaining the most basic qualitative information on the composition of pre-extinction dinosaur faunas. In this regard, it is relevant that non-hadrosaurid duckbills have now been proven to be present in subantarctic South America, whereas conclusive evidence for hadrosaurids is still lacking there and further south, in Antarctica. No remains of duck-billed dinosaurs (hadrosaurid or not) have yet been reported for Australia and New Zealand, although no Late Cretaceous continental deposits with dinosaurs are known in these regions [only partial remains from coastal deposits, (*72*)]. Late Cretaceous faunas from Indomadagascar are better documented, where the absence of duck-billed dinosaurs is more compelling in suggesting these may have never arrived into particularly isolated regions of Gondwana. Additional discoveries from underexplored regions in the global south will continue to uncover important information on the similarities and differences among pre-extinction ecosystems of the world and whether they can be related to patterns of extinction upon the K-Pg asteroid impact (*73*).

## MATERIALS AND METHODS

### Experimental model and subject details

The anatomical information used for both our comparisons and phylogenetic analyses was obtained from the literature and from direct observation of specimens housed in public repositories. The specimens mentioned below were observed directly:

1) *Huallasaurus australis*, Natural History Museum Bernardino Rivadavia, Buenos Aires, Argentina (MACN-RN 02, MACN-RN 142, MACN-RN 142B, MACN-RN 143, MACN-RN 144, MACN-RN 145, MACN-RN 146, and MACN-RN 826).

2) *Secernosaurus koerneri*, The Field Museum of Natural History (FMNH), The Field Museum, Chicago, USA (FMNH PP13423).

3) *Bonapartesaurus rionegrensis*, Vertebrate Paleontology Collection at the National University of Tucuman and the Fundación Miguel Lillo (Tucumán, Argentina), (MPCA-Pv SM2).

### Specimen provenance and geological setting

The specimens studied here were found in the Río de las Chinas Valley located 350 km to the north of the city of Punta Arenas, Ultima Esperanza Province (Magallanes Region, Chile). The studied outcrops extend along the valley in N-S direction, near to the international border with Argentina (Fig. 1). The fossil-bearing levels are assigned to the Dorotea Formation [Upper Campanian to Danian; (*21*, *74*)], which corresponds to the upper section of the continental-marine sedimentary succession that fills the Magallanes/Austral Basin. This basin was formed during the breakup of Gondwana and the opening of the Atlantic Ocean (*75*). This succession conformably overlies the Campanian to lower Maastrichtian Tres Pasos Formation (*76*) and is unconformably overlain by the Paleogene sequences of the Man Aike/Río Turbio formations of middle to late Eocene age (*77*, *78*). The Dorotea Formation represents a transition between shallow marine and continental environments (*79*–*81*). Specifically, it has been interpreted as a transition from a shallow marine shelf-edge to tide-dominated delta and fluvial systems (*79*–*82*).

The Dorotea Formation mainly comprises greenish gray and reddish brown sandstones with abundant conglomerate and siltstone lenses, thin beds of sandy calcareous concretions, and mudstones of 900 to 1200 m thickness (*79*). Along the succession, there are fossil-bearing levels with bivalves, ammonites, gastropods, sharks, plesiosaurs, mosasaurs, frogs, turtles, dinosaurs, mammals, fossil wood, and leaves (*22*, *66*, *73*, *79*, *80*).

The material here studied comes from the upper section of the Dorotea Formation in the Río de las Chinas Valley, consisting of brown sandy mudstone (60-cm thickness) with coal lenses and reddish brown fine-grained sandstone (20-cm thickness) levels [see "Column Hadrosaur level 2" (CHA-2); Fig. 1]. Numerous small- to medium-sized hadrosauroid bones were found in the sandy mudstone level and larger bones in the sandstones. The bones are preserved three-dimensionally and show linear and angular fragmentation. In addition, a 0.5-m-thick silicified trunk was found in the surface debris on a topographic slope. Apparently, this layer with hadrosaur bones extends laterally for about 5 km to the northeast, exposing itself at several points in the valley. The most notable differences have to do with the grain size at the different points where this layer is exposed. Further stratigraphic studies are needed to determine whether this entire layer originated from a single depositional event and whether bones exposed at other points also correspond to *Gonkoken*. The depositional environment of these levels with hadrosauroid bones is interpreted as the distal section of a floodplain in a continental environment (fluvial channels), with low energy. An age estimation using U-Pb detrital zircon for the levels below and above the hadrosauroid horizons provided values of 71.7 ± 1.2 Ma and 70.5 ± 5.0 Ma (*21*, *22*). An early Maastrichtian age (Late Cretaceous) for the fossil-bearing levels is most likely. Although relative error for the lower level allows for a maximum age of 72.9 Ma (shortly before the Campanian-Maastrichtian limit of 72.1 Ma), the fact that datings used detrital zircons rather than volcanic rocks imply the fossil-bearing levels could be younger.

### Character dataset and taxa

For the phylogenetic analyses (and subsequent biogeographical analyses), we used a modified version of the matrix of a recent phylogenetic analysis of all duck-billed dinosaurs (Hadrosauroidea) by Rozadilla *et al.* (*4*), with the most extensive character and taxon sample published to date. This matrix was originally built by Xing *et al.* (*38*) and posteriorly implemented by Kobayashi *et al.* (*83*, *84*). We rescored/redefined 156-character states and added 5 characters (see the Supplementary Materials). Aside from *Gonkoken*, we also added *Gobihadros mongoliensis* and *Huehuecanauhtlus tiquichensis*. As in the previous work (*4*), *Lapampasaurus* was left out from the results because its extremely partial remains (only 11 of 365 characters) preclude any relevant inference of its phylogenetic affinities. Inclusion in our analysis did not alter the topologies of the four most parsimonious trees (MPTs) and placed this South American taxon within Saurolophinae but retrieved it closest to the Japanese *Kamuysaurus*.

The character distribution was modified with Mesquite 3.70 (*85*). The characters were coded on the basis of bones belonging to adult or subadult individuals of *Gonkoken nanoi*. The character states of juvenile individuals were only coded for characters that do not show notable ontogenetic variation in duck-billed dinosaurs. We modified the scoring of some characters of *Huallasaurus australis* based on direct observation by three of the coauthors (S.S.-A., P. C.-C., and J.A.-M.). We also modified the scoring of some characters of *Secernosaurus koerneri* and *Bonapartesaurus rionegrensis* based on published images and direct observations by A.O.V. (*Secernosaurus koerneri*) and P.C.-C. (*Bonapartesaurus rionegrensis*). The resulting matrix included 77 species-level taxonomic units (three non-hadrosauroid iguanodontians and 74 hadrosauroids) scored for 365 equally weighted characters.

### Parsimony analysis

The maximum parsimony analysis was carried out in TNT version 1.6 (*86*). The non-hadrosauroid iguanodontian *Ouranosaurus nigeriensis* was selected as the outgroup. We use a “New Technology Search” using sectorial search, ratchet, drift, and tree fusing, which was set to find the minimum length tree 100 times. This search was followed by a “Traditional Search” using a tree bisection-reconnection (TBR) branch-swapping algorithm. All characters were treated as equally weighted and unordered. For all the trees, Bremer support was calculated for each node to assess its robustness by computing decay indices. Bootstrap proportions were also calculated with TNT, setting the analysis for 5000 replicates using heuristic searches (see fig. S7).

### Templeton tests

We used a Templeton test to assess the significance of different hypotheses where *Gonkoken* was forced into alternative phylogenetic positions, evaluating changes in the number of steps. We randomly used MPTs obtained from the unconstrained analysis to compare with the forced topologies, applying a one-sided Wilcoxon signed-rank test to the differences in character transformations between trees. We assessed the forced positions of *Gonkoken* as the basalmost hadrosauroid, and as a member of the Austrokritosauria (table S3). We assessed whether the difference in steps between the original and forced hypotheses was significant using Templeton Tests (*87*), implemented with a script in Tree analysis using New Technology (TNT) 1.6 (*88*).

### Selection and time calibration of the most resolved tree obtained from the parsimony analysis

Only four trees were obtained from parsimony analysis with the standard option to collapse zero-length branches. Close relationships of *Gonkoken* were fully resolved: differences among trees were all in taxa distant from *Gonkoken*, represented in a single polytomy at a basal node (see fig. S7). We selected one of the four trees to show the relationships of *Gonkoken* and for biogeographic analyses with BioGeoBEARS, which require a fully resolved tree (see below). We picked the only tree with no zero-length branches (no polytomies), which is shown in Fig. 5 (tree number 2 in TNT). To time-calibrate this tree, we ran a tip-dated Bayesian analysis with the entire topology constrained to this tree; we kept the same priors and Markov chain Monte Carlo (MCMC) parameters as in the unconstrained Bayesian analysis (see below) and used the resulting maximum clade compatibility tree (MCCT). To obtain the MCCT tree, we used the posterior files of MrBayes and the obtainDatedPosteriorTreesMrB() function of the R package paleotree v3.4.4 (*89*). Table S7 shows First Appearance Data and Last Appearance Data, which were used for the time calibration of the phylogenetic tree according to chronostratigraphic uncertainty ranges (oldest possible age and youngest possible age).

### Bayesian phylogenetic analyses (undated and tip-dated)

We conducted an undated Bayesian analysis in MrBayes v3.27a (*90*). As in the parsimony analysis, the outgroup was the non-hadrosauroid iguanodontian *Ouranosaurus nigeriensis*. We used the Mkv + Γ model of morphological evolution with ascertainment bias correction (*91*), setting the coding as variable and with six rate categories for the gamma distribution. We used two independent runs for the analysis with 40 million MCMC generations, sampling every 1000 generations and discarding 50% of the samples as burn-in. The posterior distributions for each parameter were checked to confirm that the effective sample size was >200, and the deviation of split 17 frequencies was below 0.05.

In addition, we performed a tip-dated Bayesian analysis in MrBayes v3.27a (*90*) using the same settings for the model of morphological evolution as in the undated analysis (see above). The root of the tree was calibrated using an exponential distribution with a minimum age of 129.4 Ma and a mean of 139.4 Ma. The minimum age of the root was based on the maximum age of the non-hadrosauroid iguanodontian *Ouranosaurus nigeriensis*. For calibrating the tree tips, we used a uniform distribution based on the radiometric age uncertainty of the fossils scored in the morphological matrix (*92*).

We used a normal distributed clock rate prior, with a mean of 0.02253 and an SD of 0.00839. This prior was extracted from the previously obtained undated Bayesian consensus tree and using the packages ape v. 2.3 (*93*) and fitdistrplus 1.1-8 (*94*) from R 2022.07.2 + 576 (*95*) following the methodology described in (*96*). The best model following the Bayesian information criterion was the gamma-distributed clock rate (shape = 5.92176, rate = 262.84887). However, preliminary analyses of the data showed problems of convergence between the two independent runs using this model; therefore, a normally distributed model was preferred for our analysis. We used the fossilized birth-death (FBD) model using an exponential net diversification prior with rate 1, a beta fossil sampling proportion prior with shape parameters α = 1 and β = 1, a beta turnover prior with shape parameters α = 1 and β = 1, and an extant sampling proportion of 1. We modeled branch rate variation using the independent gamma rate relaxed clock model with an exponential distribution of rate 10. We used diffuse priors for the FBD model and the clock variance, which reflect our prior uncertainty in the distribution of these parameters.

We used two independent runs for the analysis with 40 million MCMC generations, sampling every 1000 generations and discarding 50% of the samples as burn-in. The posterior distributions for each parameter were checked to confirm that the effective sample size was >200, and the deviation of split frequencies was below 0.05.

In the 50% majority-rule consensus tree of the tip-dated Bayesian analysis, *Gonkoken* is placed in a polytomy with *Eotrachodon*, *Zhanghenglong*, and *Plesiohadros* (fig. S9). Resolution improved in the undated Bayesian analysis, when geological ages were not allowed to influence the topology; as in the parsimony analysis, *Gonkoken* was retrieved closer to the clade that includes *Lophorhothon*, *Huehuecanauhtlus*, and Hadrosauridae than to *Eotrachodon*, *Zhanghenglong*, or *Plesiohadros* (fig. S8). This difference between tip-dated and undated analysis may result from the fact that the ages of the nearby *Nanningosaurus* and *Gobihadros* are very uncertain, with large possible temporal ranges. Regardless, in both approaches, many polytomies persisted regarding the close relationships of *Gonkoken*. Even in the maximum posterior probability trees (see auxiliary supplementary files), most relationships were recovered in less than 50% of trees. We therefore decided not to select any tree resulting from Bayesian phylogenetic analysis for the purposes of biogeographic analyses (which require fully resolved trees).

### Statistical biogeographic analyses

We implemented an s-DIVA (*60*–*62*) in RASP 4.2 (*62*) to reconstruct the ancestral areas for all internal nodes of the phylogeny obtained in our parsimony analysis. Six general areas were considered: Asia (A), Europe (B), Laramidia (C), Appalachia (D), South America (E), and Africa (F). Before the s-DIVA, we performed a phylogenetic analysis in TNT 1.6 to obtain the phylogenetic trees that were used in this analysis. The phylogenetic analysis recovered six most parsimonious trees without collapsing zero-length branches (1335 steps, CI: 0.390; RI: 0.816), to obtain fully resolved trees. The six trees were loaded in RASP 4.2, as well as the strict consensus tree, which was used for the graphical representation of the s-DIVA results. The “Allow Reconstruction (Slow)” option was selected to implement the method of calculating the frequency of ancestral states for each node (*97*). The Supplementary Materials contain the details of the results.

We also used the R package BioGeoBEARS v.1.12 (*59*) as a second approach for estimating the biogeographic history of duck-billed dinosaurs and the ancestral areas for *Gonkoken*. This method evaluates alternative models for extinction, dispersal, cladogenesis, and founder effect (*98*). BioGeoBEARS requires fully resolved time-calibrated trees. We did not use any tree obtained from Bayesian inference since most relationships of *Gonkoken* remained unresolved in the 50% majority-rule tree (*99*). Therefore, we used the only tree with no zero-length branches obtained from parsimony analysis, which we time-calibrated by running a tip-dated Bayesian analysis with the entire topology constrained to this tree (see above). The terminal taxa were coded for the following biogeographical provinces: Asia, Europe, Laramidia, Appalachia, South America, and Africa. We used a dispersal matrix that considers different dispersal probabilities between land masses. The dispersal weights between geographical areas were obtained from (*5*). We evaluated six different models: DIVALIKE and DIVALIKE + j (*60*), DEC and DEC + j (*100*), and BAYAREALIKE and BAYAREALIKE + j (*101*). We permitted only four simultaneous areas. For each model, we obtained the values of log-Likelihood (lnL), Dispersal (d), Extinction (e), Founder effect (j), Corrected Akaike Information Criterion (AICc), and AICc Weight (AICc + wt). These models were compared using their AICc. The model with the lowest AICc value (and higher AICc + wt) was selected as the model with the best fit to the data.

## Supplementary Materials

### This PDF file includes:

Supplementary Text Figs. S1 to S16 Tables S1 to S9 Legends for data files S1 to S8 References

## Notes

View/request a protocol for this paper from *Bio-protocol*.
