# Supplementary material for: Relict duck-billed dinosaurs survived into the last age of the dinosaurs in subantarctic Chile
Source: Sci Adv. 2023 Jun 16;9(24):eadg2456. doi: 10.1126/sciadv.adg2456 (PMC10275600; doi:10.1126/sciadv.adg2456)
Supplement: Supplementary file 1 — Supplementary Text Figs. S1 to S16 Tables S1 to S9 Legends for data files S1 to S8 References [file sciadv.adg2456_sm.pdf]

Science Advances
AAAS

Supplementary Materials for

Relict duck-billed dinosaurs survived into the last age of the dinosaurs in subantarctic Chile

Jhonatan Alarcón-Muñoz *et al.*

Corresponding author: Jhonatan Alarcón-Muñoz, jhoalarc@gmail.cl; Alexander O. Vargas, alexvargas@uchile.cl

Sci. Adv. **9**, eadg2456 (2023)
DOI: 10.1126/sciadv.adg2456

The PDF file includes:

Supplementary Text
Figs. S1 to S16
Tables S1 to S9
Legends for data files S1 to S8
References

Other Supplementary Material for this manuscript includes the following:

Data files S1 to S8

Supplementary Text

Taphonomy

Different analyses were carried out including hydraulic equivalence, bone modification, assemblage data analyses and Voorhies groups classification. The overall study of taphonomic aspects followed traditional literature (102,103). Only bones of duck-billed dinosaurs were recovered at the site, as is common in the fossil record of these dinosaurs, probably related to their gregarious behavior (19). Most of the bones were found scattered without superposition and without any evident articulation. The size of the elements ranges between 4.8 cm and 48.1 cm, the former value corresponding to the length of the smallest caudal vertebral centrum (CPAP 5351) and the latter to the length of the largest femur (CPAP 5359). 44.5% of the elements are complete and 93% present some extent of fracture. A minimal number of three individuals (MNI) has been estimated based on the number of right femora and left humeri. Relative abundance of elements from each skeletal region is shown in Table S1.

The transport of a bone element in water depends on multiple factors, such as shape, density, size and degree of articulation, in addition to the flow velocity. Disarticulated bones form distinctive classification groups ("Voorhies groups") according to the flow speed to which they are subjected (104-107). Group 1 corresponds to bones of lower density and relatively low mass, which are affected by light currents (vertebrae and phalanges, for example); group 2 is formed by elements that are eliminated gradually, mainly due to traction; and group 3 corresponds to bones that tend to resist transport (due to their greater density and size). We found a predominance of Group 3 elements, which is consistent with the interpretation of an original deposit with the influence of low stream current transportation (see Table S2). Fluvial transport is therefore the most likely explanation for the disarticulation and scattering of the elements within the quarry.

Hydraulic equivalence allows comparing the settling velocity of fossils with that of clasts to estimate whether they were deposited at similar flow velocities, that is, by a similar process (105). Assemblages that accumulated due to fluvial processes should have a high proportion of material in hydraulic equivalence with the sediment matrix, whereas a lack of hydraulic equivalence indicates that fluvial processes were not the dominant cause of accumulation (although it does not rule out fluvial influence, 108). The average length of (complete) bones is 22 cm; large bones range between 48 cm and 32 cm and represent 35% of the complete bones. The remaining 65% range between 24.6 cm and 4.8 cm in length. When comparing these dimensions with quartz grains, using the equivalence table of Behrensmeyer (105), most of the elements are found to be equivalent to gravel-sized grains. This means that the current velocity that transported these bones should have also been enough to transport gravel-sized grains. The matrix of the bonebed however consists predominantly of sandy mudstones with coal lenses and fine-grained sandstones. This discrepancy between the hydraulic equivalence of the bones and the matrix suggests that flow competence would have been insufficient to transport the bones a long distance, thus ruling out allochthonous assembly. This result is consistent with the interpretation based on the Voorhies group classification.

Analysis of pre-fossilization and diagenetic weathering are precluded due to current climatic conditions at the site (*i.e.*, snow cover during most of the year, temperature fluctuations

and strong winds during the summer) which have an important influence on the state of preservation, accelerating weathering and erosion.

Abrasion stages can be represented by a number from 0 to 3 where 0 represents pristine fossil surfaces without signs of abrasion, and 3 corresponds to fossil bones with extremely well-rounded edges (106,109). The elements at the bonebed show low levels of abrasion (84.5% with stage 0 and 15.5% with stage 1) that suggests minimal transport for most specimens. As already mentioned, practically all the bones are incomplete. 93.3% of the elements present fractures, most of which correspond to transverse and longitudinal fractures as caused by trampling, or contemporary exposure of the fossil material to temperature variations (climate) that produce the expansion and contraction of the material. No spiral fractures have been found in the analyzed fossils.

The different analyses above support the idea that the elements were either not transported or underwent minimal transport. This leads us to conclude that the bones are close to the original thanatocoenosis and probably to the habitat of these animals, being parauthoctonous and synchronic to some degree. The surface of many of the bones is damaged due to their exposure to current extreme weather conditions, precluding an appropriate bone modification analysis. The present data rule out death by predation and scavenging since there are no bite marks on the elements and no predator teeth have been recovered in the quarry. All lines of taphonomic evidence indicate that the reasons for the death of the individuals are biological. Considering this interpretation, the dominance of elements from Voorhies Group III is unusual. However, it may be the result of a flooding event, which would have removed elements from Voorhies Groups 1 and 2 at a time when the skeletons were exposed.

List of rescored and redefined characters in the data set of Rozadilla et al. (4)

1) *Secernosaurus koerneri* (character 258). Changed from 1 to ?, since the distal half of the scapula is not preserved.

2) *Secernosaurus koerneri* (character 259). Changed from 0 to ?, since the scapula is incomplete.

3) *Secernosaurus koerneri* (character 278). Changed from ? to 1, based on fig. 12 of (110).

4) *Secernosaurus koerneri* (character 290). Changed from 0 to 1, based on calculated proportions.

5) *Secernosaurus koerneri* (character 293). Changed from 2 to 1 since the supraacetabular process does not extend far enough ventrally to reach the middle of the iliac blade.

6) *Secernosaurus koerneri* (character 303). Changed from 0 to 1, as the thickening of the postacetabular process is due to its dorsomedial rotation.

7) *Secernosaurus koerneri* (character 312). Changed from 1 to 0 since, based on our observations, the pubic peduncle of the pubis is shorter and more robust compared with other hadrosauroids, see (37).

8) *Bonapartesaurus rionegrensis* (character 247). Changed from ? to 0, since the chevrons are shorter than the caudal neural spines (9).

9) *Bonapartesaurus rionegrensis* (character 291). Changed from 0 to 1 since the apex of the supraacetabular process is in an anterodorsal position with respect to the caudal tuberosity of the ischial peduncle of the ilium (9).

10) *Bonapartesaurus rionegrensis* (character 335). Changed from 1 to 0, based on the original description given in (9).

11) *Kelumapusaura machi* (character 291). Changed from 0 to 1 since the apex of the supraacetabular process is in an anterodorsal position with respect to the caudal tuberosity of the ischial peduncle of the ilium (4).

12) *Huallasaurus australis* (character 287). Changed from 0 to 1 based on our observations (4).

13) *Gonkoken nanoi* (character 263). Redefinition (new character state added). A fourth state (3) was added for the pseudoacromion process, which refers to the ventral curvature of this structure.

14) *Laiyangosaurus youngi* (character 17). Changed from 2 (undefined character in character list) to 1. Based on the description of (111).

15) *Parasaurolophus walkeri* (character 89). Changed from 2 (undefined character in character list) to 1 based on (112).

16) *Olorotitan arharensis* (character 89). Changed from 2 (undefined character in character list) to 1 based on (112).

17) *Hypacrosaurus altispinus* (character 89). Changed from 2 (undefined character in character list) to 1 based on (112).

18) *Lambeosaurus lambei* (character 91). Changed from 2 (undefined character in character list) to 1 based on (112).

19) *Lambeosaurus magnicristatus* (character 91). Changed from 2 (undefined character in character list) to 1 based on (112).

20) *Hypacrosaurus altispinus* (character 91). Changed from 2 (undefined character in character list) to 1 based on (112).

21) *Gobihadros mongoliensis* (character 99). Changed from ? to 2, based on (5), and fig. 2 of (31).

22) *Gobihadros mongoliensis* (character 256). Changed from 1 to 0, based on the description and fig. 22 (31).

23) *Gobihadros mongoliensis* (character 258). Changed from 0 to 1, based on the description and fig. 22 of (31).

24) *Gobihadros mongoliensis* (character 293). Changed from 1 to 2 based on the description and fig. 20 of (31).

25) *Gobihadros mongoliensis* (character 309). Changed from 3 to 1, based on the description and fig. 28 of (31).

26) *Gobihadros mongoliensis* (character 332). Changed from 1 to 0, based on the description and fig. 29 of (31).

27) *Adynomosaurus arcanus* (character 318). Changed from 2 to 1, based on the description and fig. 8 of (39).

28) *Adynomosaurus arcanus* (character 322). Changed from 0 to ?, since the structure is not preserved (39).

29) *Adynomosaurus arcanus* (character 323). Changed from 0 to ?, since the structure is not preserved (39).

30) *Adynomosaurus arcanus* (character 324). Changed from 0 to ?, since the structure is not completely preserved (39).

31) *Adynomosaurus arcanus* (character 325). Changed from 0 to ?, since the structure is not preserved (39).

32) *Adynomosaurus arcanus* (character 326). Changed from 0 to 1, based on fig. 8 of (39).

33) *Adynomosaurus arcanus* (character 327). Changed from 0 to ?, since the structure is not completely preserved (39).

34) *Adynomosaurus arcanus* (character 328). Changed from 0 to ?, since the distal portion of the ischial shaft is not preserved (39).

35) *Adynomosaurus arcanus* (character 329). Changed from 0 to ?, since the distal portion of the ischial shaft is not preserved (39).

36) *Adynomosaurus arcanus* (character 330). Changed from 0 to ?, since the distal portion of the ischial shaft is not preserved (39).

37) *Lophorhothon atopus* (character 334). Changed from ? to 0, based on the description and fig. 13 of (32).

38) *Lophorhothon atopus* (character 348). Changed from ? to 0, based on fig. 3 (32).

39) *Lophorhothon atopus* (character 349). Changed from ? to 0, based on fig. 6 of (32).

40) *Tethyshadros insularis* (character 4). Changed from 1 to 0, based on (48).

41) *Tethyshadros insularis* (character 6). Changed from 1 to 0, based on (48).

42) *Tethyshadros insularis* (character 9). Changed from ? to 0, based on (48).

43) *Tethyshadros insularis* (character 10). Changed from ? to 0, based on (48).

44) *Tethyshadros insularis* (character 14). Changed from 1 to ?, based on (48).

45) *Tethyshadros insularis* (character 15). Changed from 1 to ?, based on (48).

46) *Tethyshadros insularis* (character 20). Changed from 1 to 0, based on (48).

47) *Tethyshadros insularis* (character 22). Changed from 0 to 1, based on (48).

48) *Tethyshadros insularis* (character 24). Changed from ? to 0, based on (48).

49) *Tethyshadros insularis* (character 28). Changed from 0 to 0&1, based on (48).

50) *Tethyshadros insularis* (character 29). Changed from 0 to ?, based on (48).

51) *Tethyshadros insularis* (character 35). Changed from 1 to 0, based on (48).

52) *Tethyshadros insularis* (character 38). Changed from 0 to ?, based on (48).

53) *Tethyshadros insularis* (character 39). Changed from ? to 1, based on (48).

54) *Tethyshadros insularis* (character 42). Changed from ? to 0, based on (48).

55) *Tethyshadros insularis* (character 44). Changed from ? to 0, based on (48).

56) *Tethyshadros insularis* (character 45). Changed from 1 to 0, based on (48).

57) *Tethyshadros insularis* (character 46). Changed from ? to 0, based on (48).

58) *Tethyshadros insularis* (character 49). Changed from 1 to 0, based on (48).

59) *Tethyshadros insularis* (character 54). Changed from ? to 0, based on (48).

60) *Tethyshadros insularis* (character 55). Changed from 1 to 0, based on (48).

61) *Tethyshadros insularis* (character 56). Changed from 1 to 0, based on (48).

62) *Tethyshadros insularis* (character 57). Changed from 1 to 0, based on (48).

63) *Tethyshadros insularis* (character 58). Changed from ? to 0, based on (48).

64) *Tethyshadros insularis* (character 62). Changed from 1 to 0, based on (48).

65) *Tethyshadros insularis* (character 63). Changed from 0 to 1, based on (48).

66) *Tethyshadros insularis* (character 65). Changed from 1 to 0, based on (48).

67) *Tethyshadros insularis* (character 67). Changed from 1 to 0, based on (48).

68) *Tethyshadros insularis* (character 68). Changed from 1 to ?, based on (48).

69) *Tethyshadros insularis* (character 69). Changed from 1 to ?, based on (48).

70) *Tethyshadros insularis* (character 71). Changed from 1 to ?, based on (48).

71) *Tethyshadros insularis* (character 80). Changed from 1 to ?, based on (48).

72) *Tethyshadros insularis* (character 81). Changed from 0 to 1, based on (48).

73) *Tethyshadros insularis* (character 90). Changed from ? to 0, based on (48).

74) *Tethyshadros insularis* (character 95). Changed from ? to 0, based on (48).

75) *Tethyshadros insularis* (character 96). Changed from ? to 0, based on (48).

76) *Tethyshadros insularis* (character 97). Changed from 0 to 1, based on (48).

77) *Tethyshadros insularis* (character 98). Changed from 1 to 0, based on (48).

78) *Tethyshadros insularis* (character 100). Changed from 2 to 0, based on (48).

79) *Tethyshadros insularis* (character 101). Changed from 1 to 0, based on (48).

80) *Tethyshadros insularis* (character 102). Changed from ? to 0, based on (48).

81) *Tethyshadros insularis* (character 105). Changed from 1 to ?, based on (48).

82) *Tethyshadros insularis* (character 107). Changed from ? to 1, based on (48).

83) *Tethyshadros insularis* (character 110). Changed from ? to 0, based on (48).

84) *Tethyshadros insularis* (character 111). Changed from 2 to ?, based on (48).

85) *Tethyshadros insularis* (character 126). Changed from 2 to 0, based on (48).

86) *Tethyshadros insularis* (character 130). Changed from 1 to ?, based on (48).

87) *Tethyshadros insularis* (character 132). Changed from 1 to ?, based on (48).

88) *Tethyshadros insularis* (character 135). Changed from 1 to ?, based on (48).

89) *Tethyshadros insularis* (character 136). Changed from 1 to 0, based on (48).

90) *Tethyshadros insularis* (character 148). Changed from 1 to 0, based on (48).

91) *Tethyshadros insularis* (character 170). Changed from 1 to 0, based on (48).

92) *Tethyshadros insularis* (character 178). Changed from 0 to 1, based on (48).

93) *Tethyshadros insularis* (character 184). Changed from 1 to 0, based on (48).

94) *Tethyshadros insularis* (character 186). Changed from 1 to 0, based on (48).

95) *Tethyshadros insularis* (character 189). Changed from ? to 0, based on (48).

96) *Tethyshadros insularis* (character 198). Changed from 0 to ?, based on (48).

97) *Tethyshadros insularis* (character 203). Changed from 0 to 1, based on (48).

98) *Tethyshadros insularis* (character 206). Changed from 0 to ?, based on (48).

99) *Tethyshadros insularis* (character 209). Changed from 0 to ?, based on (48).

100) *Tethyshadros insularis* (character 210). Changed from 1 to ?, based on (48).

101) *Tethyshadros insularis* (character 211). Changed from 1 to ?, based on (48).

102) *Tethyshadros insularis* (character 212). Changed from 1 to ?, based on (48).

103) *Tethyshadros insularis* (character 214). Changed from ? to 1, based on (48).

104) *Tethyshadros insularis* (character 220). Changed from ? to 0, based on (48).

105) *Tethyshadros insularis* (character 221). Changed from ? to 0, based on (48).

106) *Tethyshadros insularis* (character 229). Changed from 0 to ?, based on (48).

107) *Tethyshadros insularis* (character 230). Changed from 1 to 0, based on (48).

108) *Tethyshadros insularis* (character 233). Changed from 1 to ?, based on (48).

109) *Tethyshadros insularis* (character 235). Changed from ? to 0, based on (48).

110) *Tethyshadros insularis* (character 238). Changed from 1 to 0, based on (48).

111) *Tethyshadros insularis* (character 239). Changed from 1 to 0, based on (48).

112) *Tethyshadros insularis* (character 244). Changed from 1 to 0, based on (48).

113) *Tethyshadros insularis* (character 246). Changed from 1 to 0&1, based on (48).

114) *Tethyshadros insularis* (character 247). Changed from 1 to 0, based on (48).

115) *Tethyshadros insularis* (character 252). Changed from 1 to ?, based on (48).

116) *Tethyshadros insularis* (character 253). Changed from 0 to ?, based on (48).

117) *Tethyshadros insularis* (character 254). Changed from 0 to ?, based on (48).

118) *Tethyshadros insularis* (character 255). Changed from 0 to ?, based on (48).

119) *Tethyshadros insularis* (character 264). Changed from ? to 0, based on (48).

120) *Tethyshadros insularis* (character 265). Changed from 0 to ?, based on (48).

121) *Tethyshadros insularis* (character 266). Changed from 0 to ?, based on (48).

122) *Tethyshadros insularis* (character 269). Changed from 0 to ?, based on (48).

123) *Tethyshadros insularis* (character 270). Changed from 0 to ?, based on (48).

124) *Tethyshadros insularis* (character 272). Changed from 0 to ?, based on (48).

125) *Tethyshadros insularis* (character 273). Changed from ? to 0, based on (48).

126) *Tethyshadros insularis* (character 287). Changed from 1 to 0, based on (48).

127) *Tethyshadros insularis* (character 289). Changed from 2 to ?, based on (48).

128) *Tethyshadros insularis* (character 290). Changed from 1 to ?, based on (48).

129) *Tethyshadros insularis* (character 292). Changed from 1 to ?, based on (48).

130) *Tethyshadros insularis* (character 293). Changed from 3 to 2, based on (48).

131) *Tethyshadros insularis* (character 300). Changed from 1 to 0, based on (48).

132) *Tethyshadros insularis* (character 301). Changed from 0 to 1, based on (48).

133) *Tethyshadros insularis* (character 303). Changed from 2 to ?, based on (48).

134) *Tethyshadros insularis* (character 310). Changed from 1 to 0, based on (48).

135) *Tethyshadros insularis* (character 311). Changed from 0 to ?, based on (48).

136) *Tethyshadros insularis* (character 314). Changed from 1 to ?, based on (48).

137) *Tethyshadros insularis* (character 315). Changed from 1 to ?, based on (48).

138) *Tethyshadros insularis* (character 317). Changed from 0 to ?, based on (48).

139) *Tethyshadros insularis* (character 318). Changed from ? to 0, based on (48).

140) *Tethyshadros insularis* (character 320). Changed from 1 to ?, based on (48).

141) *Tethyshadros insularis* (character 321). Changed from 1 to ?, based on (48).

142) *Tethyshadros insularis* (character 322). Changed from 1 to 0, based on (48).

143) *Tethyshadros insularis* (character 323). Changed from ? to 0, based on (48).

144) *Tethyshadros insularis* (character 325). Changed from ? to 0, based on (48).

145) *Tethyshadros insularis* (character 331). Changed from 1 to ?, based on (48).

146) *Tethyshadros insularis* (character 332). Changed from 1 to 0, based on (48).

147) *Tethyshadros insularis* (character 336). Changed from ? to 0, based on (48).

148) *Tethyshadros insularis* (character 339). Changed from 1 to ?, based on (48).

149) *Tethyshadros insularis* (character 340). Changed from 1 to 0, based on (48).

150) *Tethyshadros insularis* (character 341). Changed from 0 to 1, based on (48).

151) *Tethyshadros insularis* (character 343). Changed from 1 to 0, based on (48).

152) *Tethyshadros insularis* (character 344). Changed from ? to 0, based on (48).

153) *Tethyshadros insularis* (character 346). Changed from 0 to ?, based on (48).

154) *Tethyshadros insularis* (character 351). Changed from ? to 0, based on (48).

155) *Tethyshadros insularis* (character 353). Changed from ? to 0, based on (48).

156) *Tethyshadros insularis* (character 354). Changed from ? to 0, based on (48).

<u>List of characters added to the data set of Rozadilla et al. (4)</u>

1) Character 361 – Maxilla, ectopterygoid ridge: continuous with jugal tubercle [0] or discontinuous [1]. Added from (5), Character 244.

2) Character 362 – Maxilla, ectopterygoid ridge: ridge extends to ventral jugal tubercle [0]; main part of shelf lies distinctly below it [1]. Added from (5), Character 245.

3) Character 363 – Maxilla, neurovascular foramina: a single very large foramen below the jugal, with a second, smaller foramen below it: absent [0]; present [1]. Added from (5), Character 247.

4) Character 364 – Maxilla, posterior dentigerous process: dentigerous margin straight [0]; posterior downturn [1] strong posterior downturn, i.e., "boomerang" shape [2]. Added from (5), Character 340.

5) Character 365 – Humerus, shape of the deltopectoral crest apex: well-rounded [0]; extending abruptly to produce a prominent angular profile [1]. Added from (113), Character 37; following (29), Character 221.

<u>Reconstruction of biogeographic history</u>

The results of the BioGeoBEARS analysis are summarized in Table S4 and Fig. S11. The best-fit model in BioGeoBEARS was the DIVALIKE + j model (AICc: 200.80), which supports a Laramidian origin for the last ancestor shared by *Gonkoken* with Hadrosauridae, and for the last ancestor shared with *Eotrachodon*, at the previous node (Fig. S11). The + j versions of all models (DEC, BAYERALIKE, and DIVALIKE) showed a better fit to the data and supported a Laramidian origin for the last ancestor shared by *Gonkoken* with Hadrosauridae, and for the last ancestor shared with *Eotrachodon*. The + j models support a strong role of founder effects (114), which is consistent with discrete events of dispersal across important barriers as proposed for Hadrosauroidea.

In s-DIVA, the last ancestor shared by *Gonkoken* and Hadrosauridae is recovered as having inhabited either Laramidia + Appalachia + South America, or Appalachia + South America, with equal probability. In turn, the previous last ancestor shared by *Gonkoken* and *Eotrachodon* inhabited Appalachia rather than Laramidia, as inferred by BioGeoBEARS (Fig. S12). Despite these differences, the results of s-DIVA are generally consistent with those of BioGeoBEARS in that they support the arrival of the ancestors of *Gonkoken* from North America. Possible reasons for the differences between the results of s-DIVA and BioGeoBEARS are that only BioGeoBEARS considers the geological age of taxa in time-calibrated trees, and that

BioGeoBEARS includes a dispersal matrix that considers different dispersal probabilities between land masses.

Conceptual experiments with a hypothetical African taxon

When the Hypothetical African taxon was placed between *Eotrachodon* and *Gonkoken*, the best model (DIVALIKE + j; AICc: 218.83, see Table S5) supported Laramidia + South America as the ancestral area for the last ancestor that *Gonkoken* shared with Hadrosauridae, and Laramidia as the most likely area for the last ancestor shared with *Eotrachodon* (Fig. S13).

When a Hypothetical African taxon was added to our BioGeoBEARS analysis as sister taxon to *Gonkoken*, the best fitting model (BAYAREALIKE + j; AICc: 210.89, see Table S6) did not show significant changes and continued to support Laramidia as the ancestral area for both the last ancestor that *Gonkoken* shared with Hadrosauridae, and the last ancestor shared with *Eotrachodon* (Fig. S15).

When a hypothetical African taxon was added to our s-DIVA analysis between *Eotrachodon* and *Gonkoken*, this led to no changes in the areas supported for the last common ancestor shared by *Gonkoken* and Hadrosauridae, or the previous ancestor last shared by *Gonkoken* and *Eotrachodon* (Fig. S14). When the hypothetical African taxon was placed as sister of *Gonkoken*, Africa was included among poorly resolved areas for the last ancestor shared by *Gonkoken* with Hadrosauridae, but Europe and Asia remained absent and were also absent in the immediately previous ancestor shared with *Eotrachodon*, which is still retrieved as North American (Fig. S16). Overall, the results of both s-DIVA and BioGeoBEARS continued to support the arrival of *Gonkoken*'s ancestors from North America in all conceptual experiments.

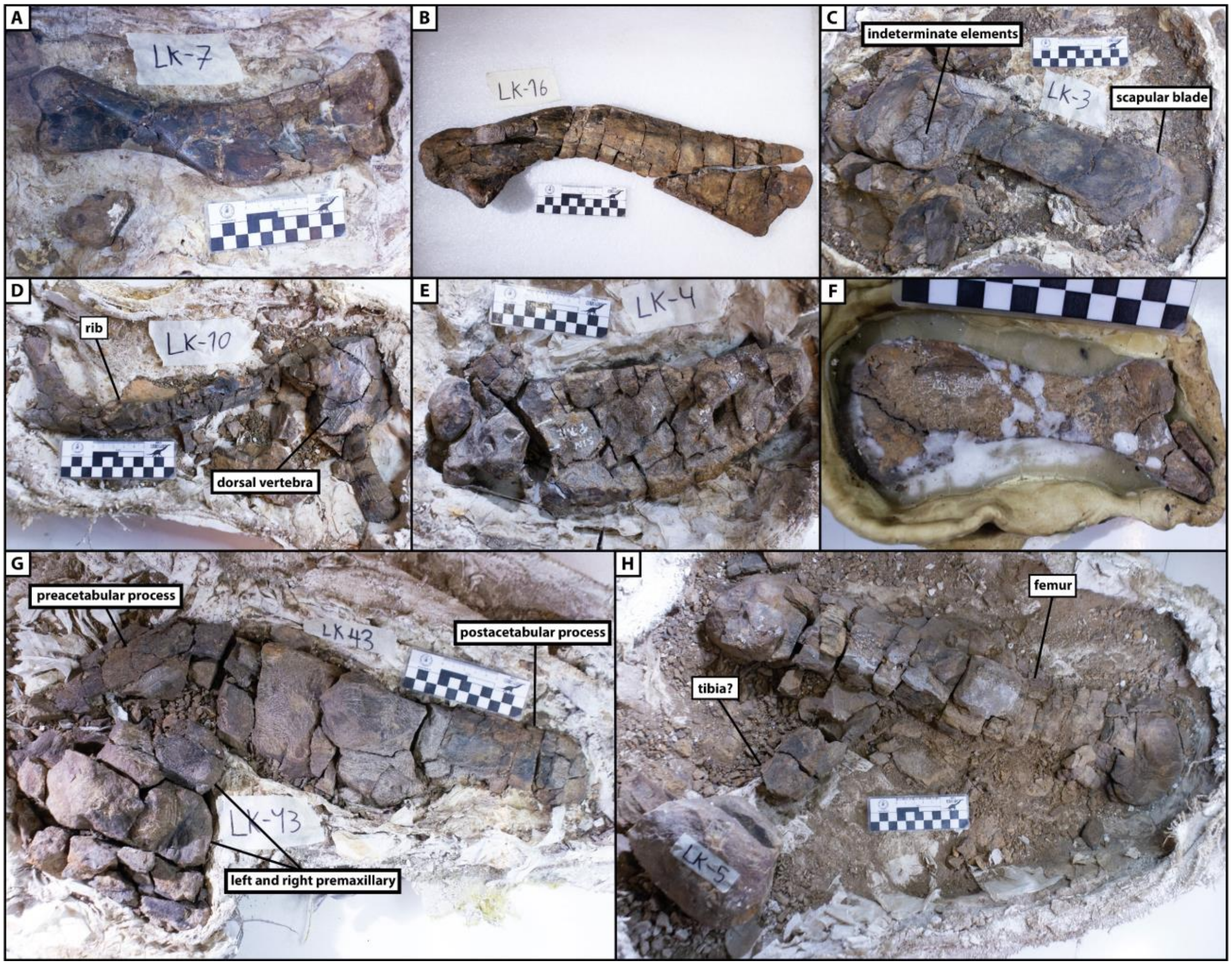

Fig. S1. Selection of some additional bone elements in the process of preparation. (**A**) left humerus of a subadult individual (LK-7) in an anteromedial view. (**B**) left scapula (LK-16) in lateral view. (**C**) left scapula in lateral view and indeterminate elements (LK-3). (**D**) right rib and dorsal vertebra (LK-10). (**E**) sacrum in lateral view (LK-4). (**F**) right pubis in medial view (LK-6). (**G**) left ilium associated with articulated premaxillaries (LK-43). (**H**) right femur in lateral view associated with a possible tibia (LK-5).

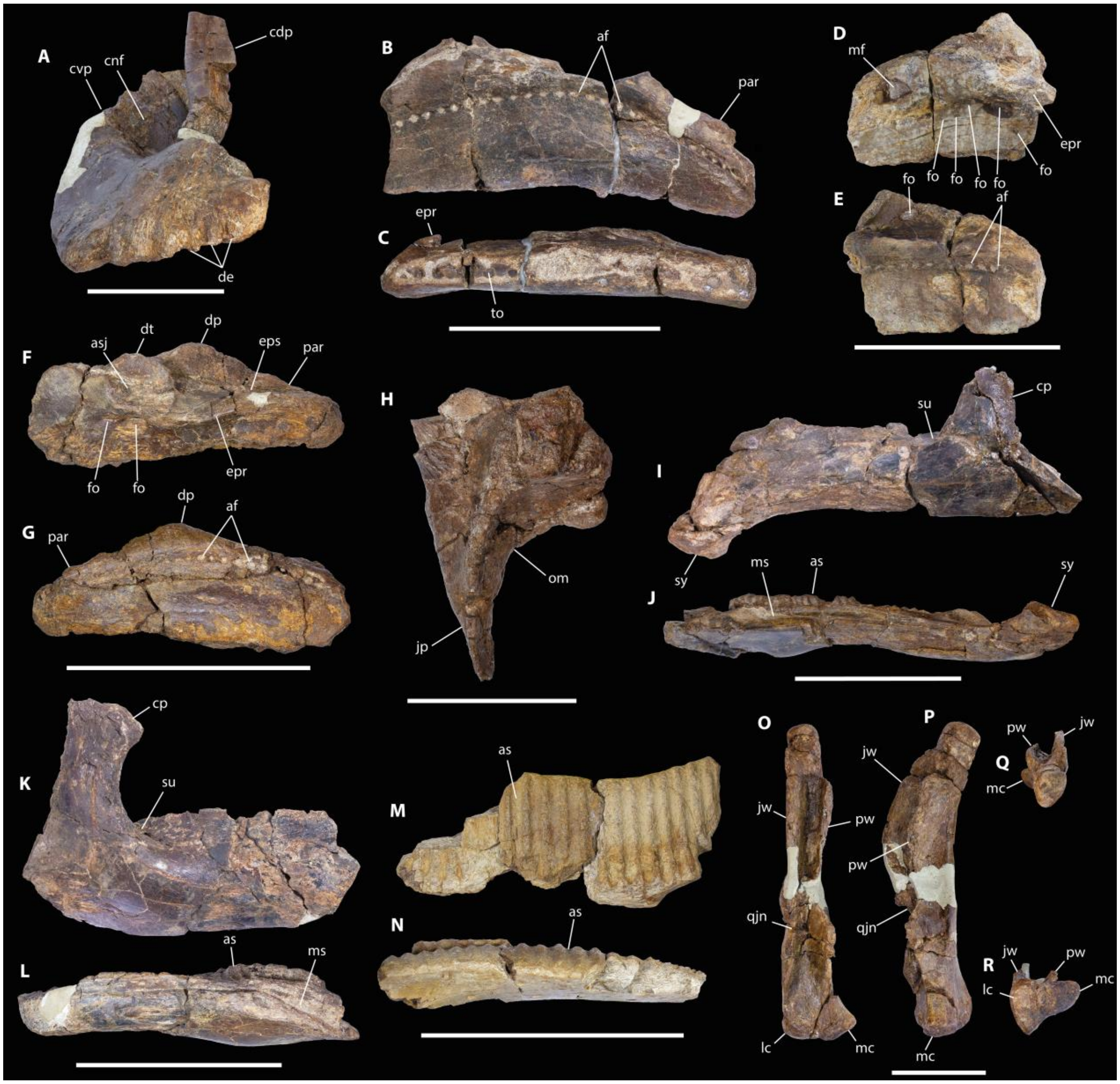

Fig. S2. Selected cranial bones of *Gonkoken nanoi*. CPAP 5337, right premaxilla in anterior view (**A**). **CPAP 5340**, right maxilla in medial (**B**) and ventral (**C**) views. **CPAP 5339**, incomplete left maxilla in lateral (**D**) and medial (**E**) views. **CPAP 5338**, incomplete left maxilla of a juvenile individual in lateral (**F**) and medial (**G**) views. **CPAP 5341**, incomplete left postorbital in medial view (**H**). **CPAP 5370**, left dentary in lateral (**I**) and ventral (**J**) views. **CPAP 5342**, incomplete right dentary in lateral (**K**) and ventral (**L**) views. **CPAP 5368**, fragment of right dentary in medial (**M**) and dorsal (**N**) views. **CPAP 5343**, right quadrate in anterior (**O**), medial (**P**), proximal (**Q**) and distal (**R**) views. Abbreviations: af: alveolar foramina; as: alveolar sulcus; asj: articular surface for the jugal; cdp: caudodorsal process; cvp: caudoventral process; cnf: circumnarial fossa; cp: coronoid process; de: denticles; dp: dorsal process; dt: dorsal tubercle; eps: ectopterygoid shelf; epr: ectopterygoid ridge; fo: foramina; jp: jugal process; jw: jugal wing; lc: lateral condyle; ms: meckelian sulcus; mf: maxillary foramen; mc: medial condyle; om: orbital margin; par: palatine ridge; pw: pterygoid wing; qjn: quadratojugal notch; su: sulcus; sy: symphysis; to: tooth. Scale bars: A, H, O-R (50 mm); B-G, I-N (100 mm).

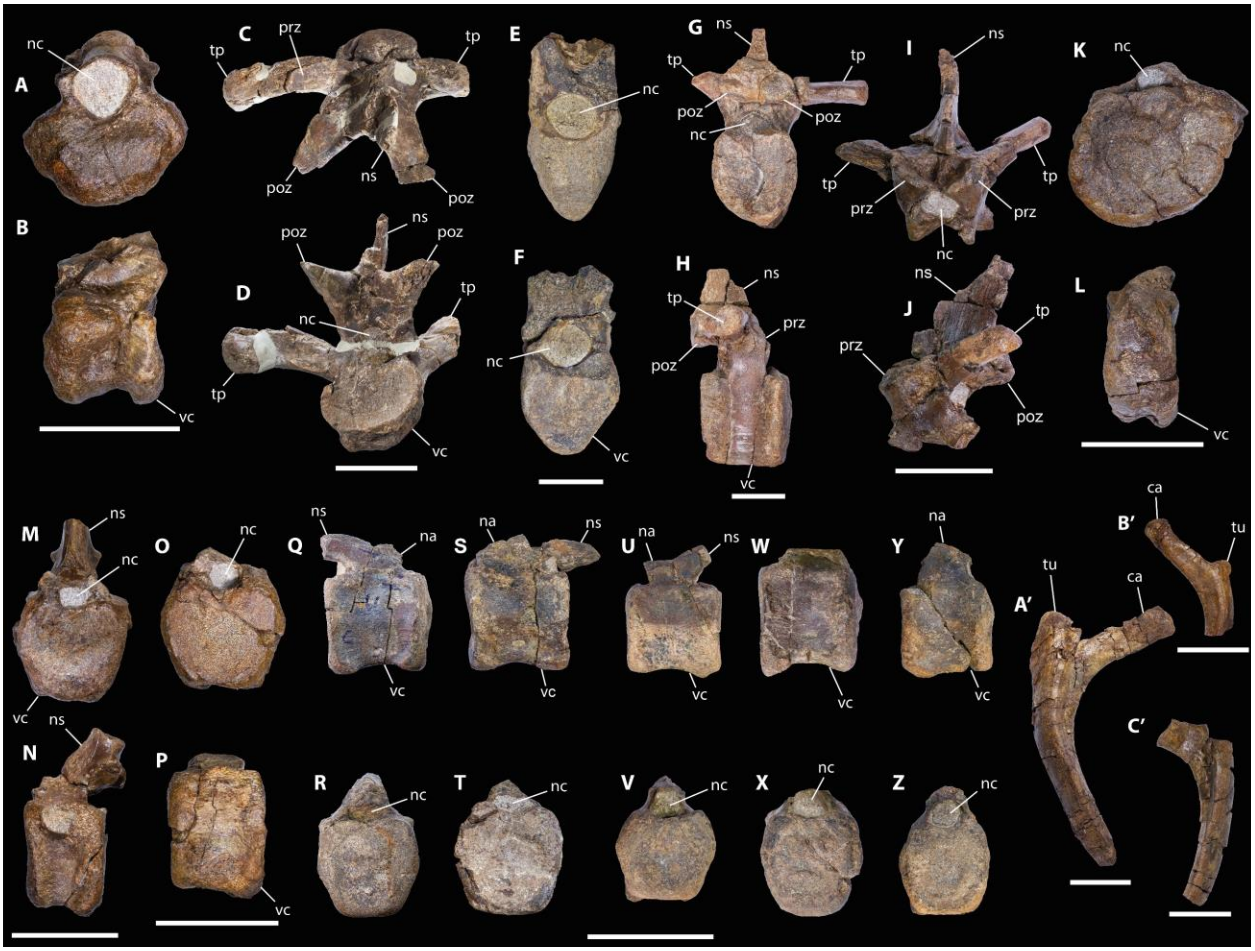

Fig. S3. Selected axial elements of *Gonkoken nanoi*. CPAP 5380, mid cervical centrum in anterior (**A**) and lateral (**B**) views. **CPAP 5344**, mid cervical vertebra in dorsal (**C**) and posterior (**D**) views. **CPAP 5345**, dorsal centrum in anterior (**E**) and posterior (**F**) views. **CPAP 5346**, dorsal vertebra in posterior (**G**) and lateral (**H**) views. **CPAP 5396**, dorsal neural arch in anterior (**I**) and lateral (**J**) views. **CPAP 5397**, proximal caudal centrum in anterior (**K**) and lateral (**L**) views. **CPAP 5398**, proximal caudal vertebra in anterior (**M**) and lateral (**N**) views. **CPAP 5399**, proximal caudal centrum in anterior (**O**) and lateral (**P**) views. **CPAP 5347** (**Q-R**), **CPAP 5348** (**S-T**), **CPAP 5349** (**U-V**), **CPAP 5350** (**W-X**) and **CPAP 5351** (**Y-Z**), mid-caudal vertebrae in lateral (**Q**, **S**, **U**, **W**, **Y**) and anterior (**P**, **S**, **U**, **W**, **Y**) views. **CPAP 5401**, right rib in anterior view (**A'**). **CPAP 5402**, proximal portion of left rib in anterior view (**B'**). **CPAP 5403**, incomplete left rib in anterior view (**C'**). Abbreviations: ca: capitulum; na: neural arch; nc: neural canal; ns: neural spine; prz: prezygapophysis; poz: postzygapophysis; tp: transverse process; tu: tuberculum; vc: vertebral centrum. Scale bars: (50 mm).

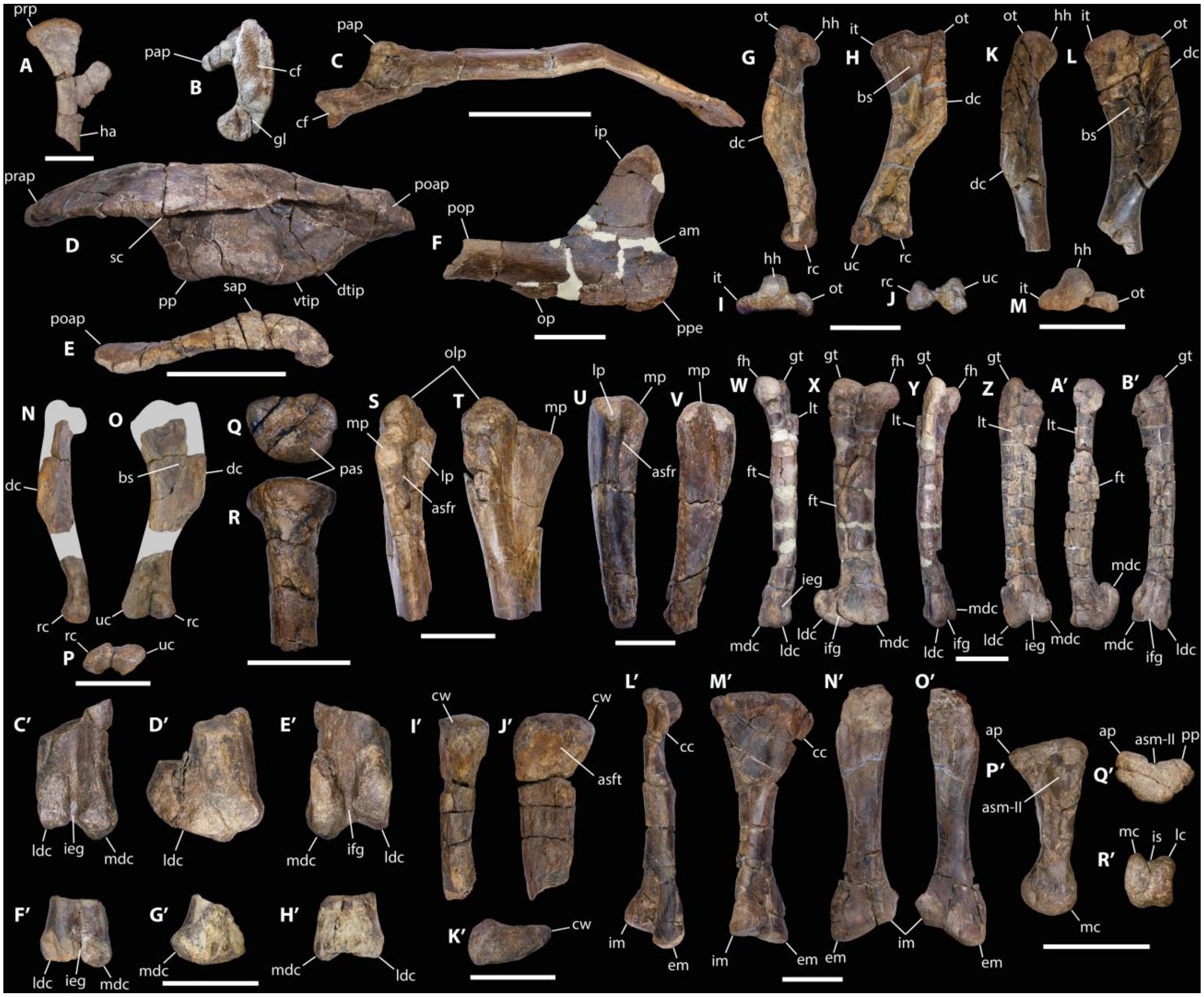

Fig. S4. Selected appendicular elements of *Gonkoken nanoi*. **CPAP 5352**, incomplete left sternum in dorsal view (**A**). **CPAP 5371**, right scapula in anterior (**B**) and dorsal (**C**) views. **CPAP 3054**, right ilium in medial view (**D**). **CPAP 5356**, postacetabular process with part of the iliac blade of a left ilium in dorsal view (**E**). **CPAP 5357**, proximal portion of right ischium in lateral view (**F**). **CPAP 5353**, complete left humerus in ventral (**G**), anteromedial (**H**), proximal (**I**), and distal (**J**) views. **CPAP 5354**, incomplete left humerus of an immature individual in ventral (**K**), anteromedial (**L**), and proximal (**M**) views. **CPAP 5369**, incomplete left humerus in ventral (**N**), anteromedial (**O**), and distal (**P**) views. **CPAP 5379**, proximal portion of right radius in proximal (**Q**) and anterior (**R**) views. **CPAP 5355**, proximal half of a left ulna in anterior (**S**), and posteromedial (**T**) views. **CPAP 5392**, incomplete right ulna in anterior (**U**), and posteromedial (**V**) views. **CPAP 5358**, left femur in medial (**W**), posterior (**X**), and lateral (**Y**) views. **CPAP 5359**, incomplete right femur in anterior (**Z**), medial (**A'**), and posterior (**B'**) views. **CPAP 5360**, distal end of the right femur in anterior (**C'**), lateral (**D'**), and posterior (**E'**) views. **CPAP 5361**, distal portion of the right femur of a juvenile individual in anterior (**F'**), lateral (**G'**), and posterior (**H'**) views. **CPAP 5363**, proximal portion of left fibula in anterior (**I'**), medial (**J'**), and proximal (**K'**) views. **CPAP 5362**, complete left tibia in anterior (**L'**), and posterior (**M'**) views. **CPAP 5372**, incomplete left tibia in lateral (**N'**), and medial (**O'**) views. **CPAP 5364**, right metatarsal III

in medial (**P’**), proximal (**Q’**), and distal (**R’**) views. Abbreviations: am: acetabular margin; asfr: articular surface for radius; asft: articular surface for the tibia; ap: anterior process; asm-II: articular surface for the metatarsal II; bs: bicipital sulcus; cf: coracoid facet; cc: cnemial crest; cw: cranial wing; dtip: dorsal tubercle of the ischial peduncle; dc: deltopectoral crest; em: external malleolus; fh: femoral head; ft: fourth trochanter; gl: glenoid; gt: great trochanter; ha: handle; hh: humeral head; ip: iliac peduncle; ieg: intercondylar extensor groove; ifg: intercondylar flexor groove; im: internal malleolus; is: intercondylar sulcus; it: inner tubercle; lp: lateral process; ldc: lateral distal condyle; lt: lesser trochanter; lc: lateral condyle; mp: medial process; mdc: medial distal condyle; mc: medial condyle; op: obturator process; olp: olecranon process; ot: outer tubercle; pap: pseudoacromion process; pas: proximal articular surface; pop: posterior process; ppe: pubic peduncle; pp: pubic process; prap: preacetabular process; prp: proximal paddle; poap: postacetabular process; rc: radial condyle; sap: supraacetabular process; sc: sacral crest; uc: ulnar condyle; vtip: ventral tubercle of the ischial peduncle. Scale bars: A, F, Q-V (50 mm); B-E, G-P, W, X, Y, Z, A’-Q’ (100 mm).

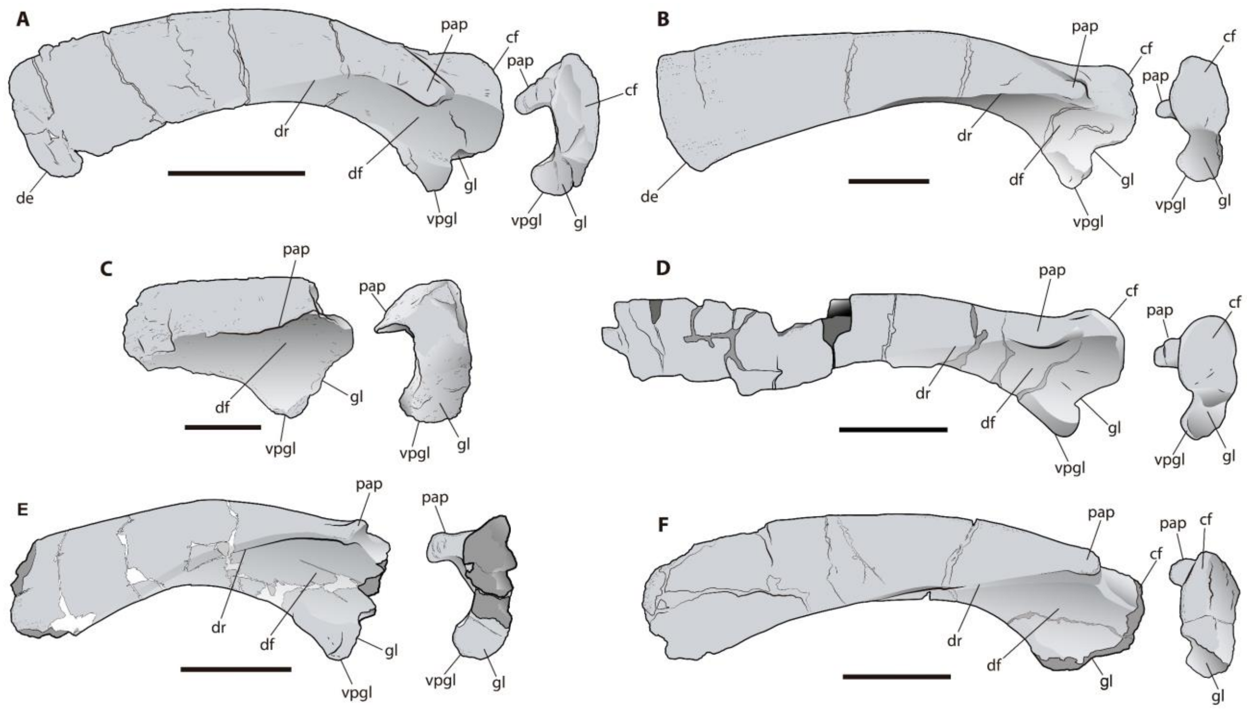

Fig. S5. Comparison of the scapula of *Gonkoken nanoi* to that of other South American hadrosauroids. (**A**) *Gonkoken nanoi* gen. and sp. nov., **CPAP 5371**, right scapula in lateral and anterior views. (**B**) *Huallasaurus australis*, **MACN RN-142**, left scapula (reversed) in lateral and anterior views (based on photographs taken by P. Cruzado-Caballero and S. Soto-Acuña). (**C**) *Lapampasaurus cholinoi*, **MPHN-Pv-01**, anterior portion of left scapula (reversed) in lateral and anterior views (based on photographs in 8). (**D**) *Kelumapusaura machi*, **MPCN-PV-810**, right scapula in lateral and anterior view (based on photographs in 4). (**E**) *Secernosaurus koerneri*, **FMNH P13423**, right scapula in lateral and anterior views (based on photographs taken by W. Simpson). (**F**) '*Willinakaqe salitrarensis*', **MPCA-Pv SM 2**, left scapula (reversed) in lateral and anterior views (based on photographs taken by P. Cruzado-Caballero). Scale bars=100 mm. Abbreviations: cf: coracoid facet; de: distal expansion; df: deltoid fossa; dr: deltoid ridge; gl: glenoid; pap: pseudoacromion process; vpgl: ventral process of the glenoid.

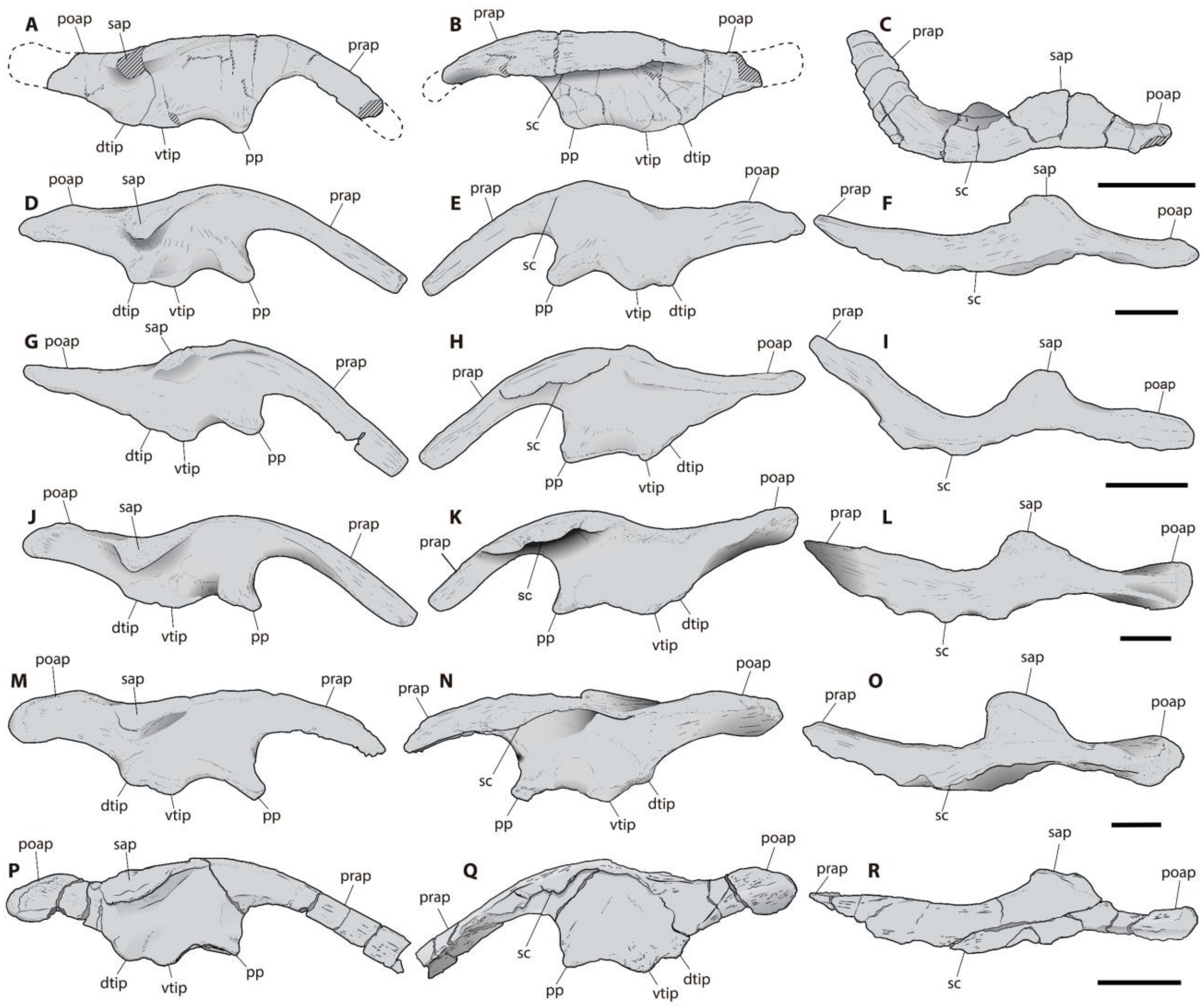

Fig. S6. Ilia of South American duck-billed dinosaurs. *Gonkoken nanoi* gen. and sp. nov. **CPAP 3054** (holotype), right ilium in lateral (**A**), medial (**B**) and dorsal views (**C**). '*Willinikaqe salitralensis*', **MPCA-Pv SM39**, right ilium in lateral (**D**), medial (**E**) and dorsal (**F**) views (based on photographs taken by P. Cruzado-Caballero). *Secernosaurus koerneri*, **FMNH P13423**, right ilium in lateral (**G**), medial (**H**) and dorsal (**I**) views (based on photographs in 110). *Huallasaurus australis*, **MACN-RN-02**, left ilium (reversed) in lateral (**J**), medial (**K**) and dorsal (**L**) views (based on photographs taken by P. Cruzado-Caballero). *Bonapartesaurus rionegrensis*, **MPCA-Pv SM2/49**, right ilium in lateral (**M**), medial (**N**) and dorsal (**O**) views (based on photographs taken by P. Cruzado-Caballero). *Kelumapusaura machi*, **MPCN-PV-811**, left ilium (reversed) in lateral (**P**), medial (**Q**) and dorsal (**R**) views (based on photographs in 4). Scale bars=100 mm. Abbreviations: dtip: dorsal tubercle of the ischial peduncle; sap: supraacetabular process; sc: sacral crest; pp: pubic process; prap: preacetabular process; poap: postacetabular process; vtip: ventral tubercle of the ischial peduncle.

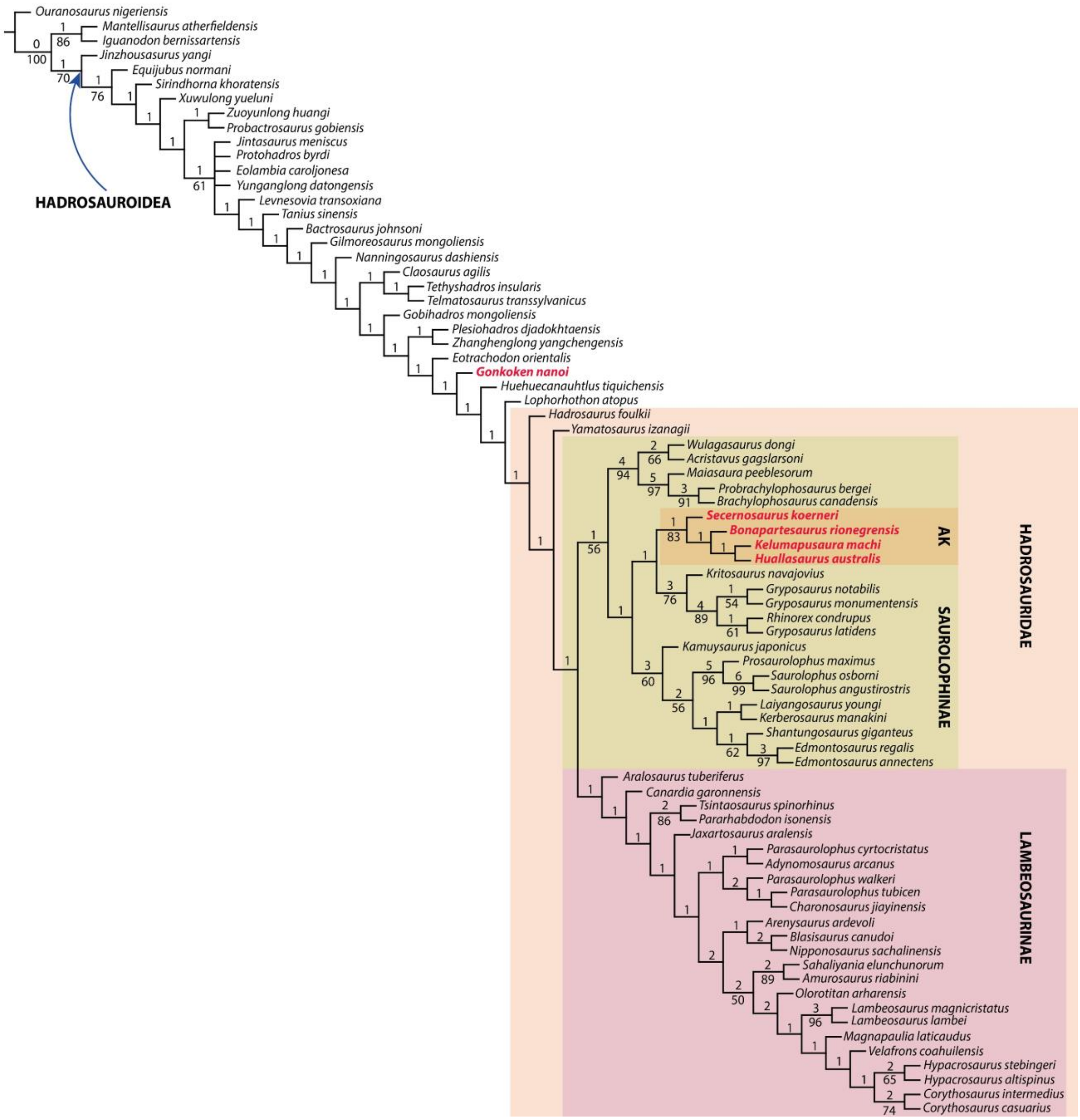

Fig. S7. Phylogenetic analyses. Strict consensus tree (parsimony analysis) of 4 MPTs of 1335 steps (CI: 0.390; RI: 0.816). Values above nodes are Bremer support. Values beneath nodes are bootstrap proportions (5000 pseudoreplicates). South American hadrosauroids are in red. AK: Austrokritosauria.

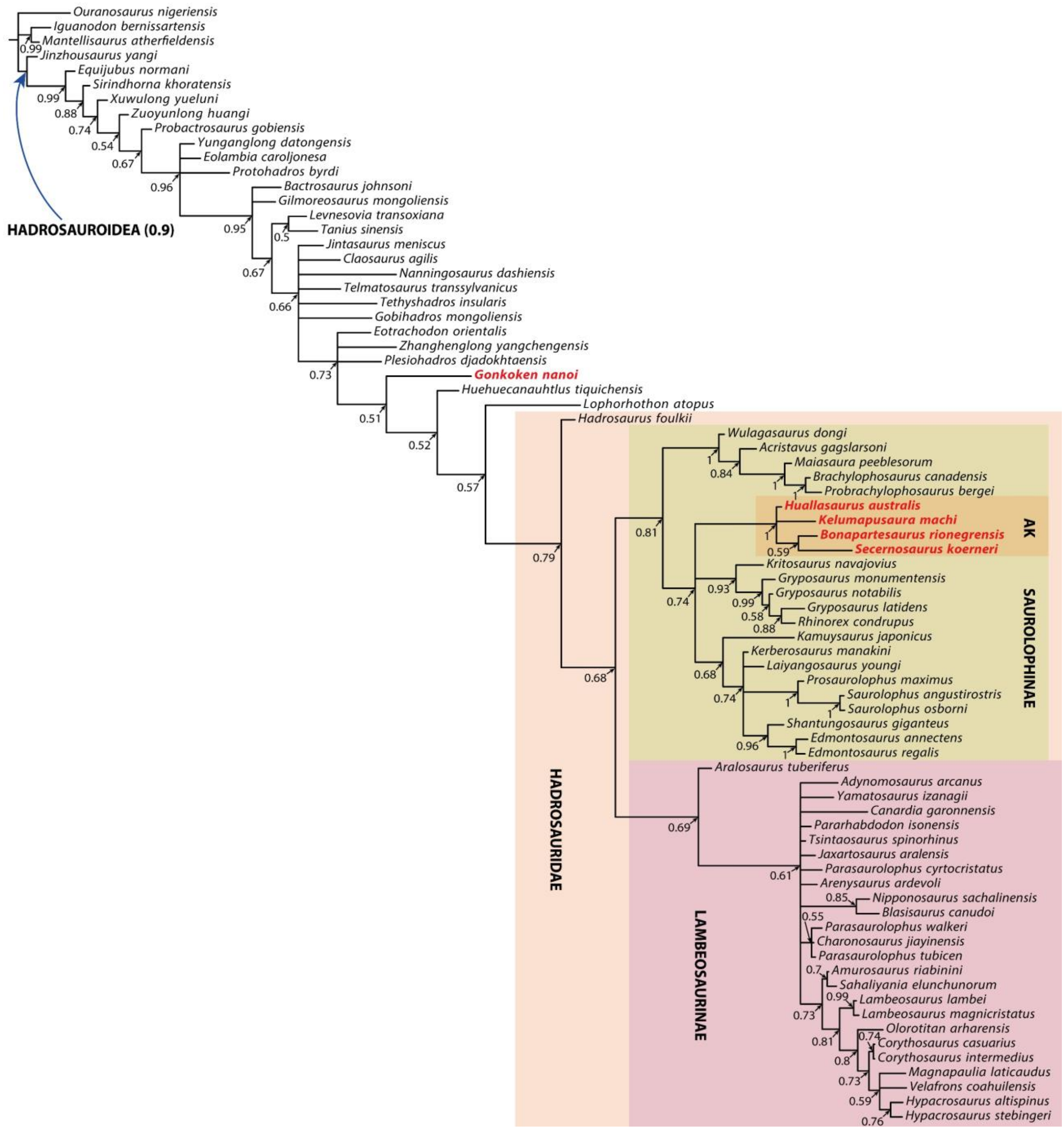

Fig. S8. 50% Majority Tree obtained by unconstrained undated Bayesian analysis. Numbers above nodes correspond to the posterior probabilities. South American hadrosauroids are in red. AK: Austrokritosauria.

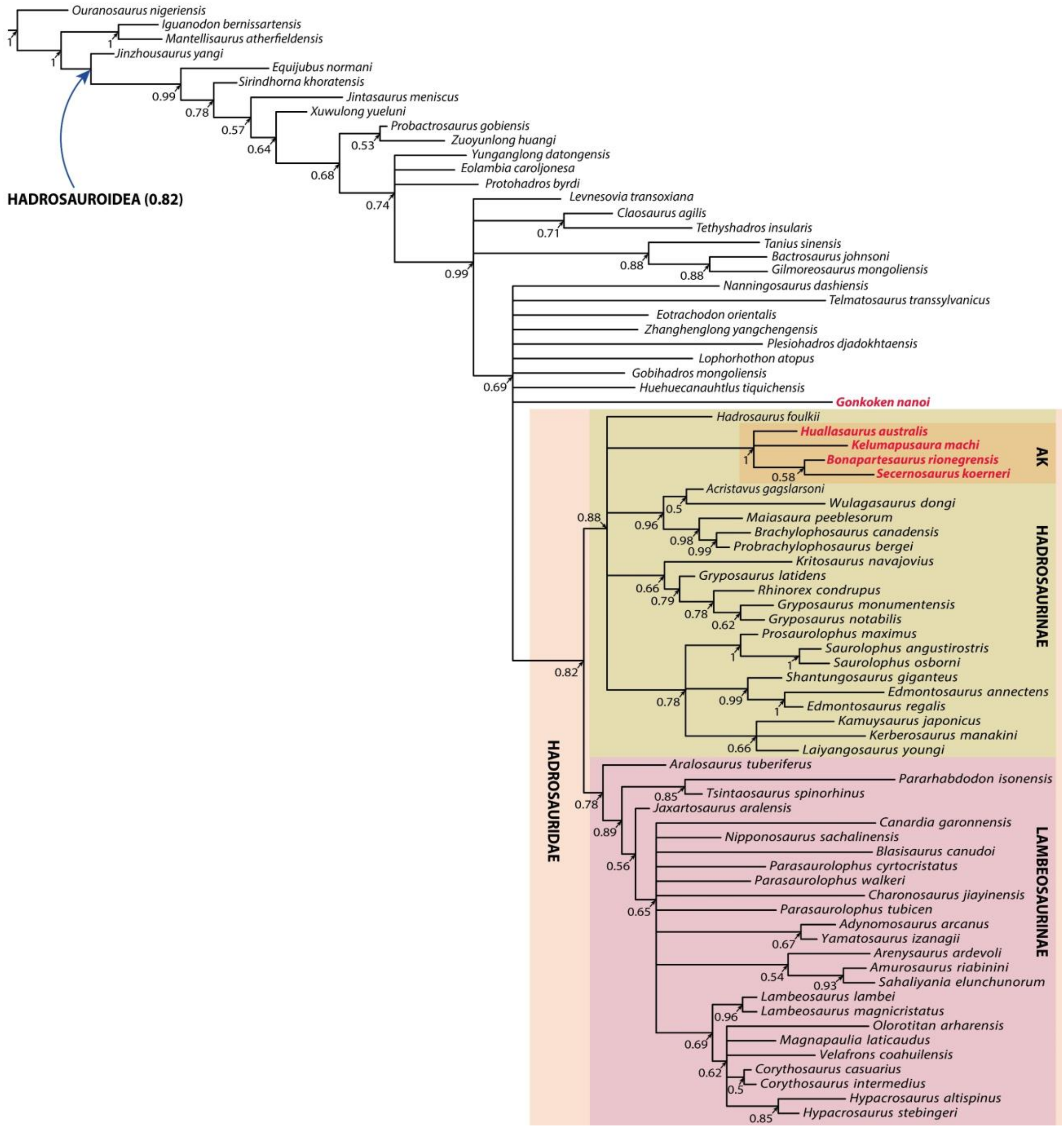

Fig. S9. 50% Majority rule tree obtained from the unconstrained tip-dated Bayesian analysis. Unlike other analyses, the clade Hadrosaurinae (Saurlophinae + *Hadrosaurus*) was recovered. Numbers above nodes correspond to the posterior probabilities. South American hadrosauroids are in red. AK: Austrokritosauria.

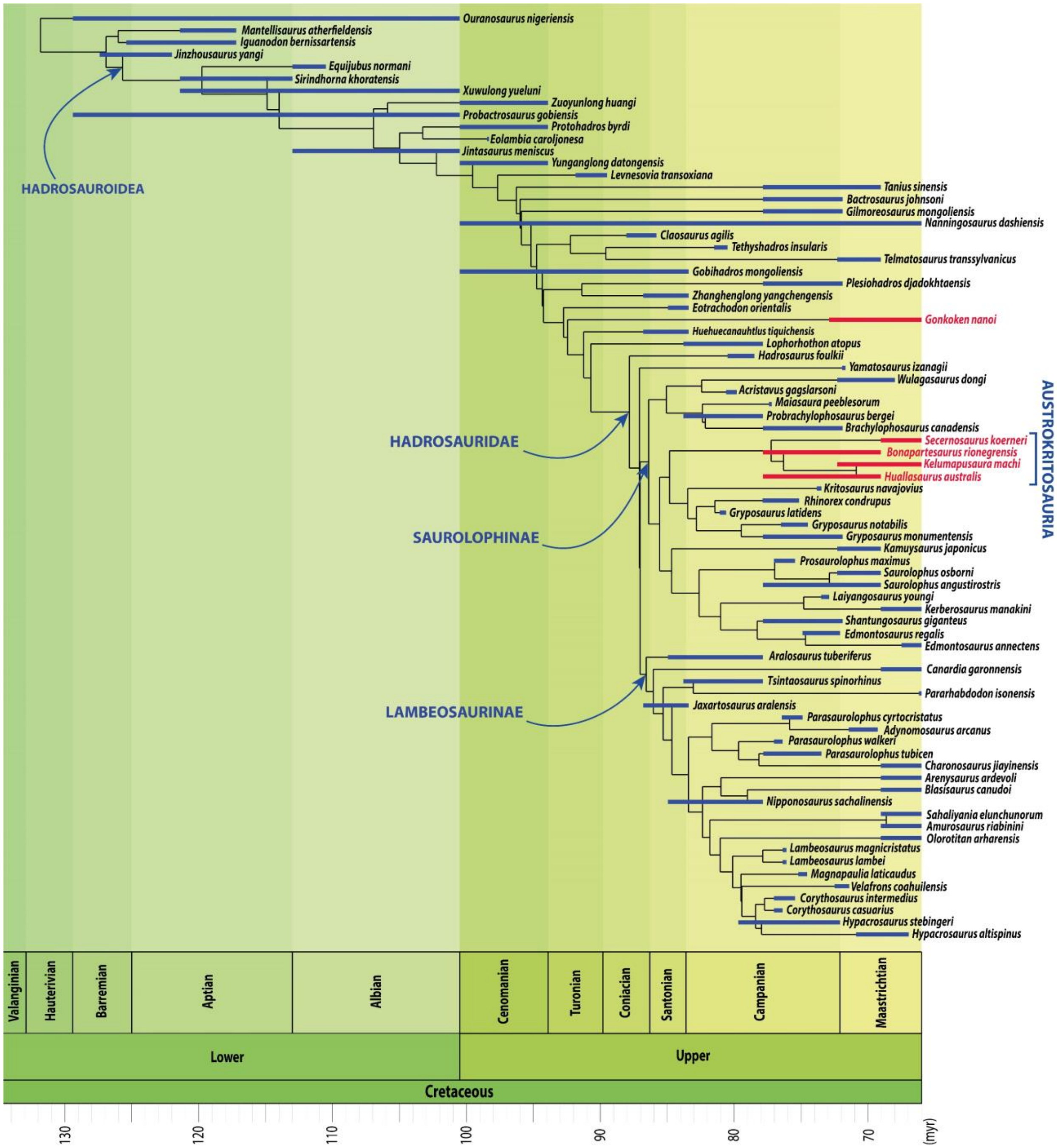

Fig. S10. Chronostratigraphic uncertainty ranges superimposed on a time-calibrated tree obtained by parsimony analysis (see Methods for selection and time calibration of this tree). South American hadrosauroids are in red.

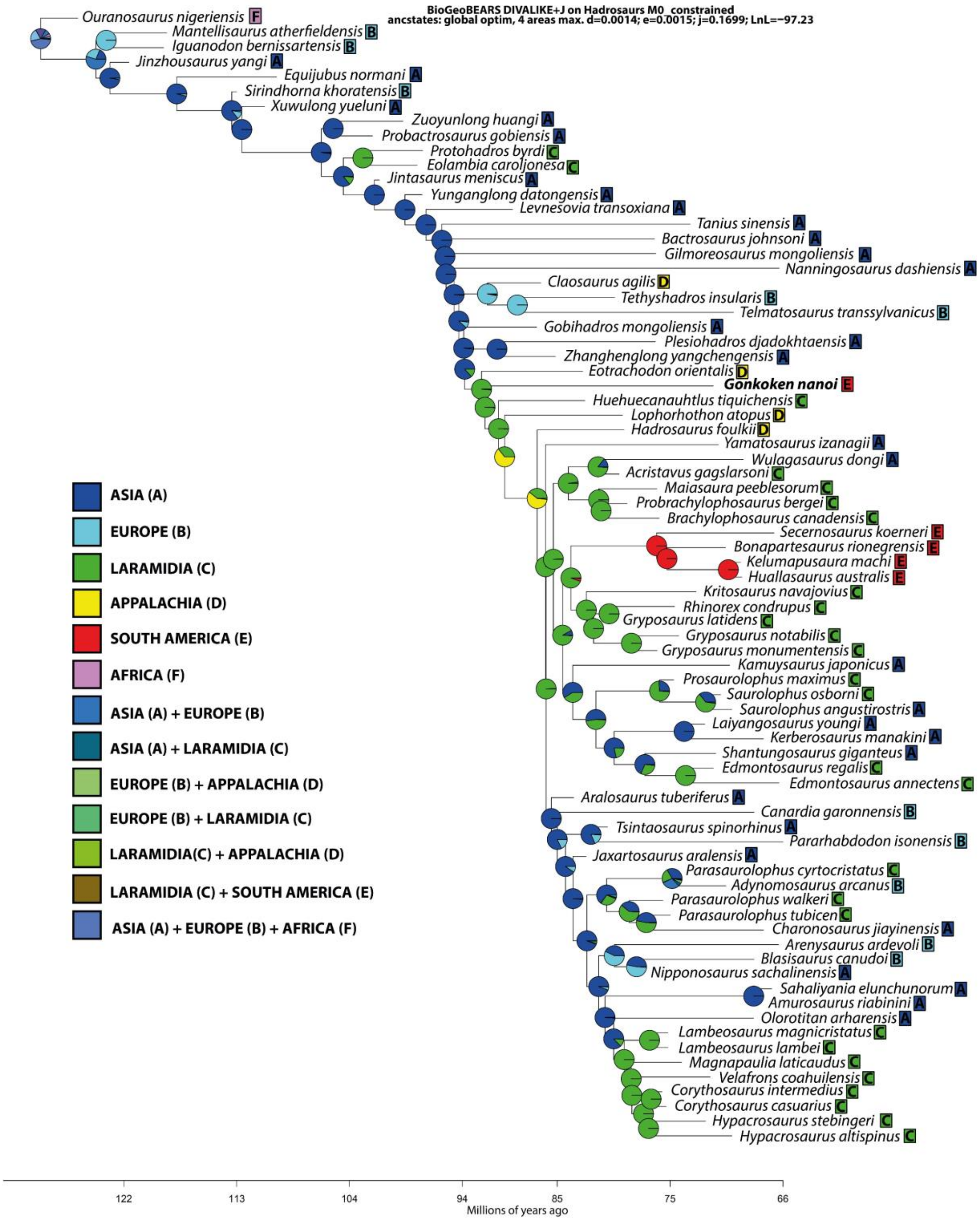

Fig. S11. Results of Biogeographical analyses. Time-calibrated tree with the results of the BioGeoBEARS analysis (DIVALIKE + j model).

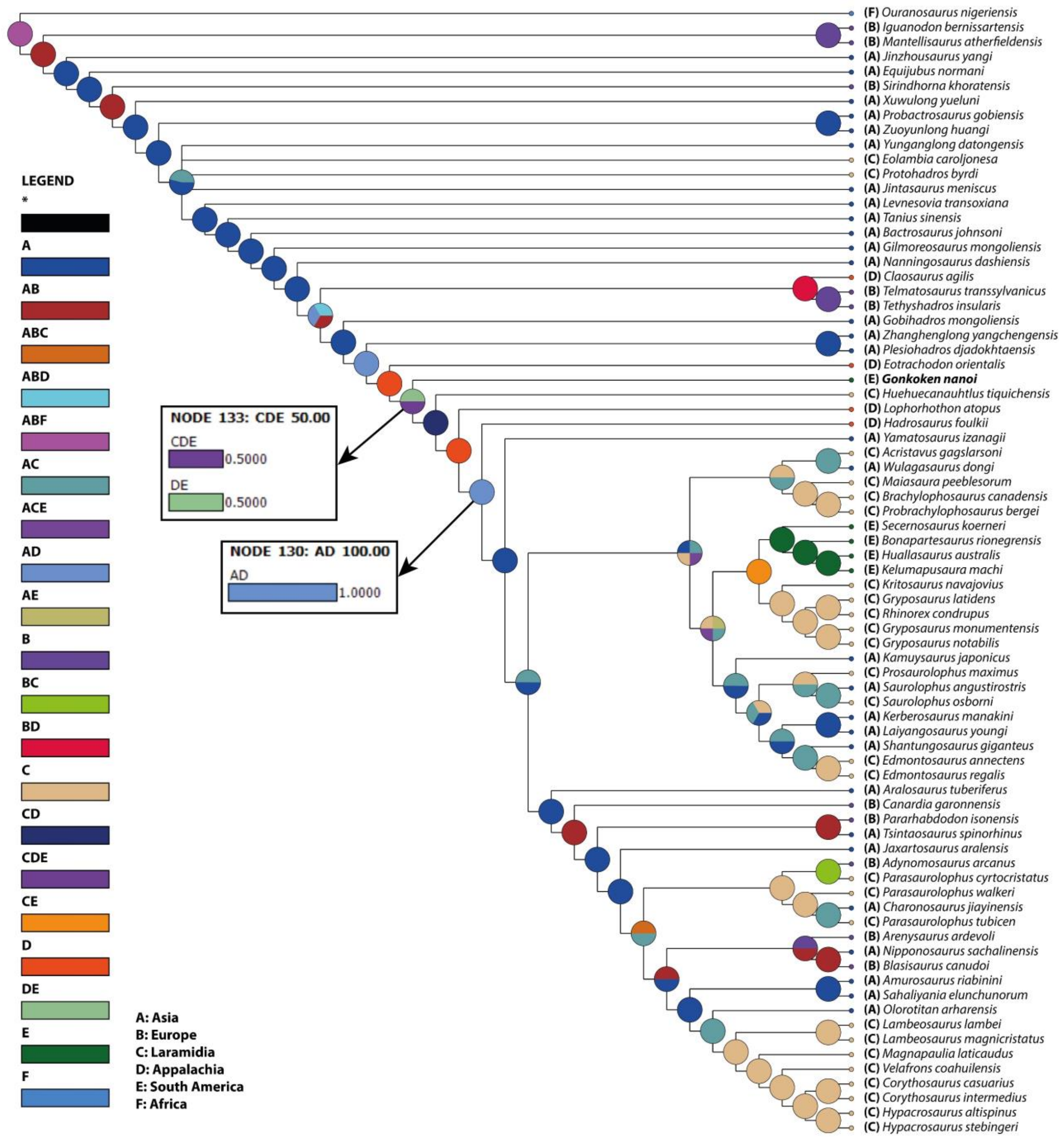

Fig. S12. Results of Biogeographical Analyses with s-DIVA. Node 130: Hadrosauridae; Node 133: *Gonkoken* + *Huehuecanauthlus* + *Lophorhothon* + Hadrosauridae.

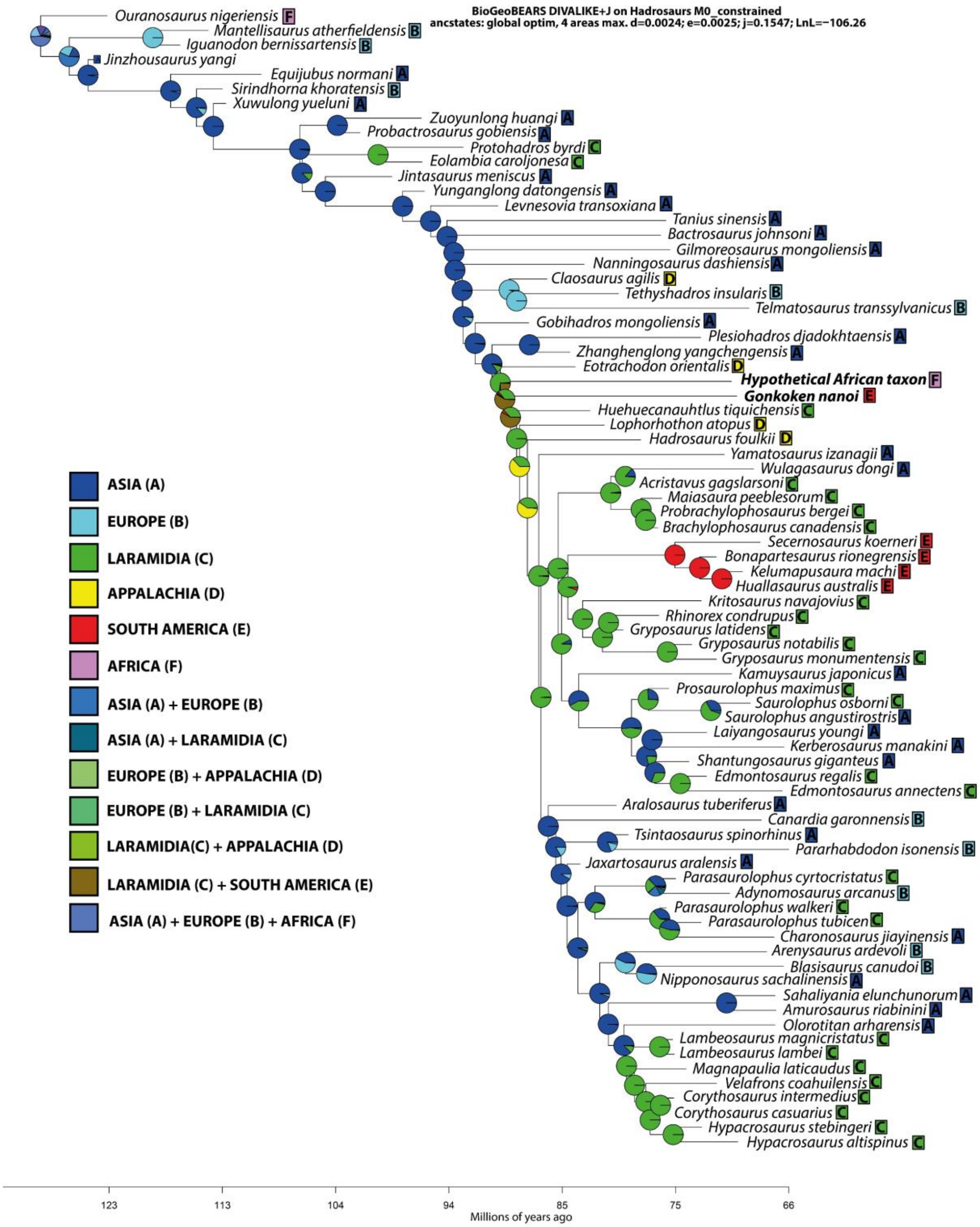

Fig. S13. Conceptual experiments with BioGeoBEARS with a "Hypothetical African taxon" between *Eotrachodon* and *Gonkoken*. Time-calibrated tree with the results of the BioGeoBEARS analysis (DIVALIKE + j model).

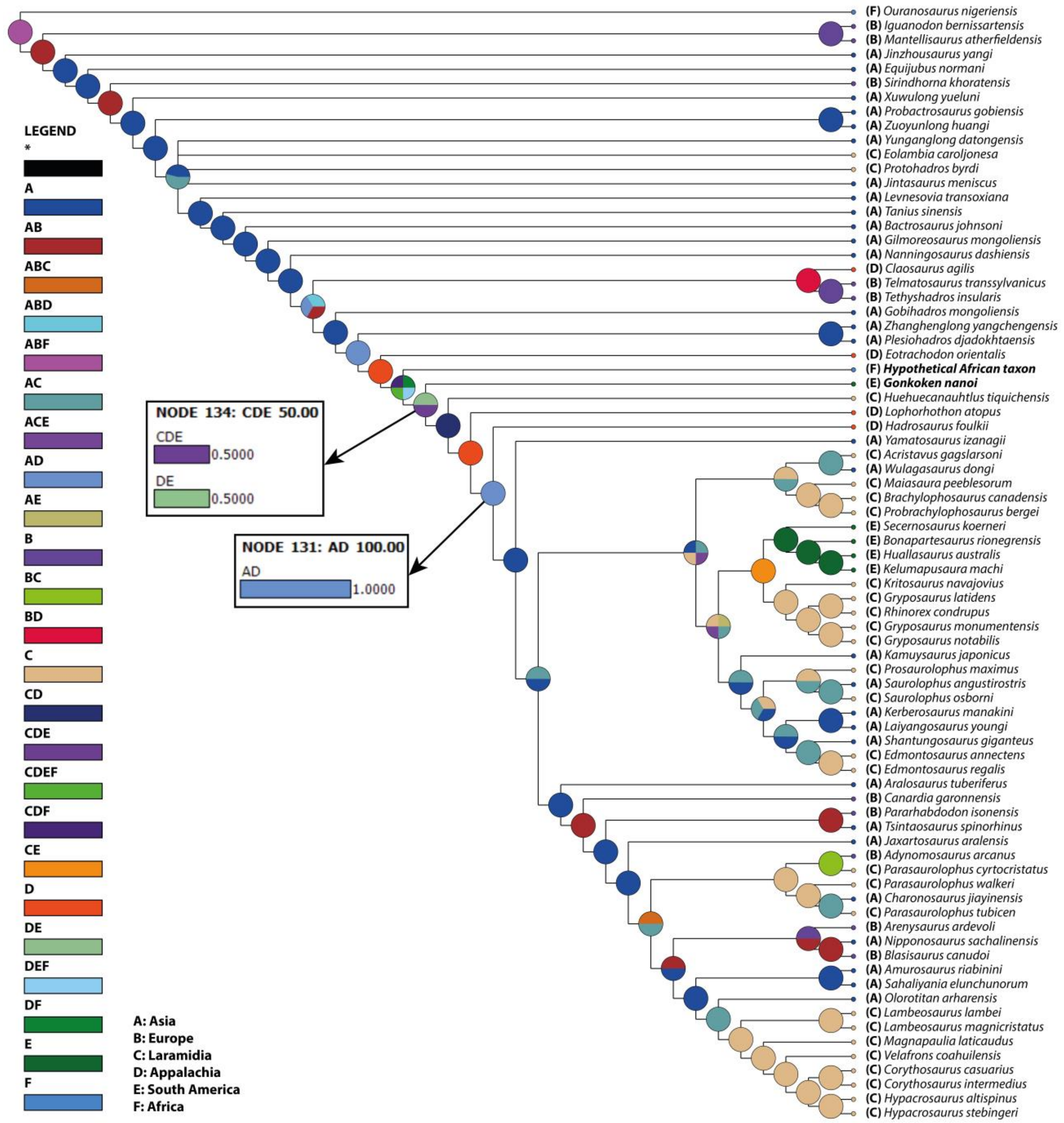

Fig. S14. Conceptual experiment with s-DIVA with the "Hypothetical African taxon" between *Eotrachodon* and *Gonkoken*. Node 131: Hadrosauridae; Node 134: *Gonkoken* + *Huehuecanauthlus* + *Lophorhothon* + Hadrosauridae.

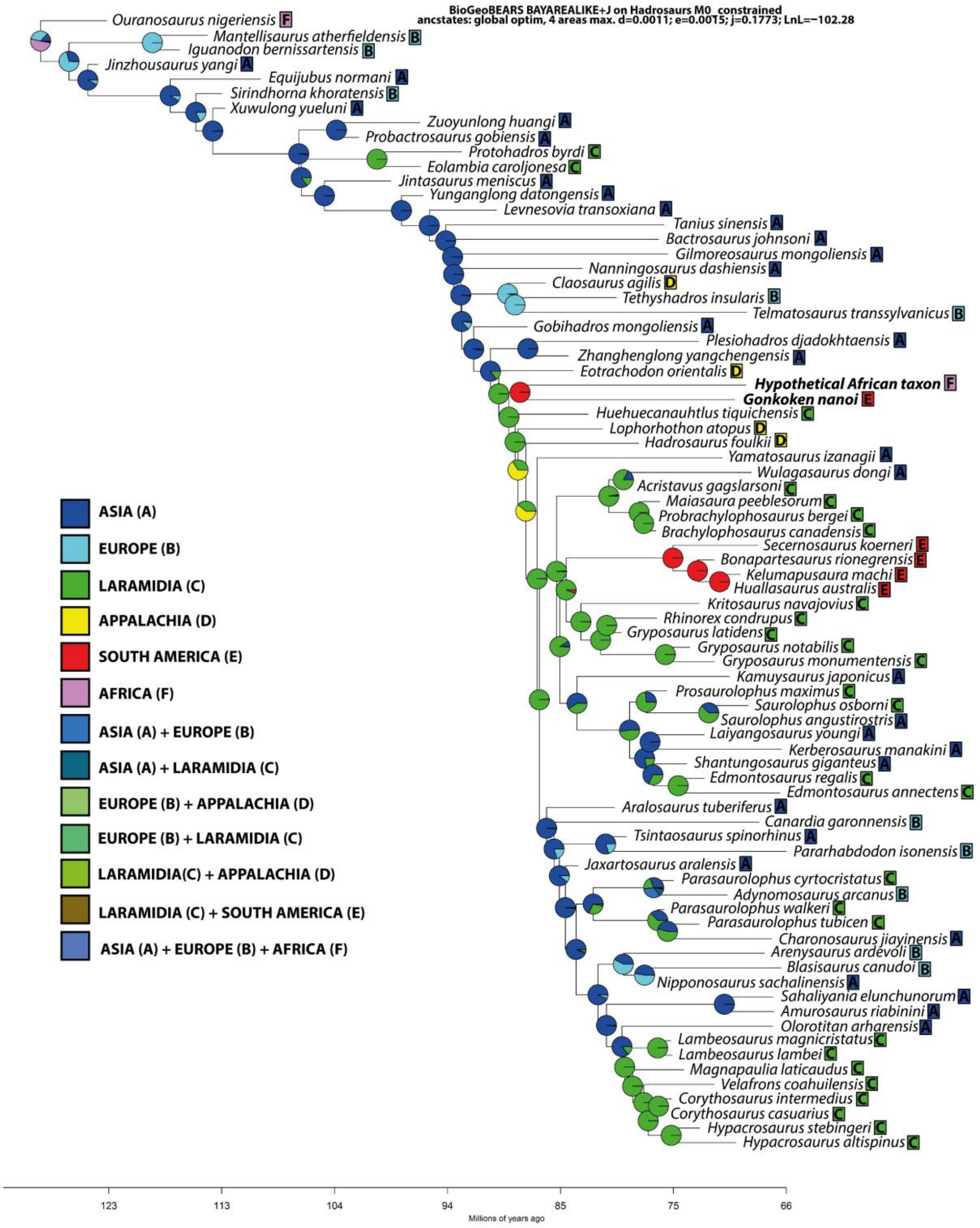

Fig. S15. Conceptual experiments with BioGeoBEARS with a "Hypothetical African taxon" as sister taxon of *Gonkoken*. Time-calibrated tree with the results of the BioGeoBEARS analysis (BAYAREALIKE + j model).

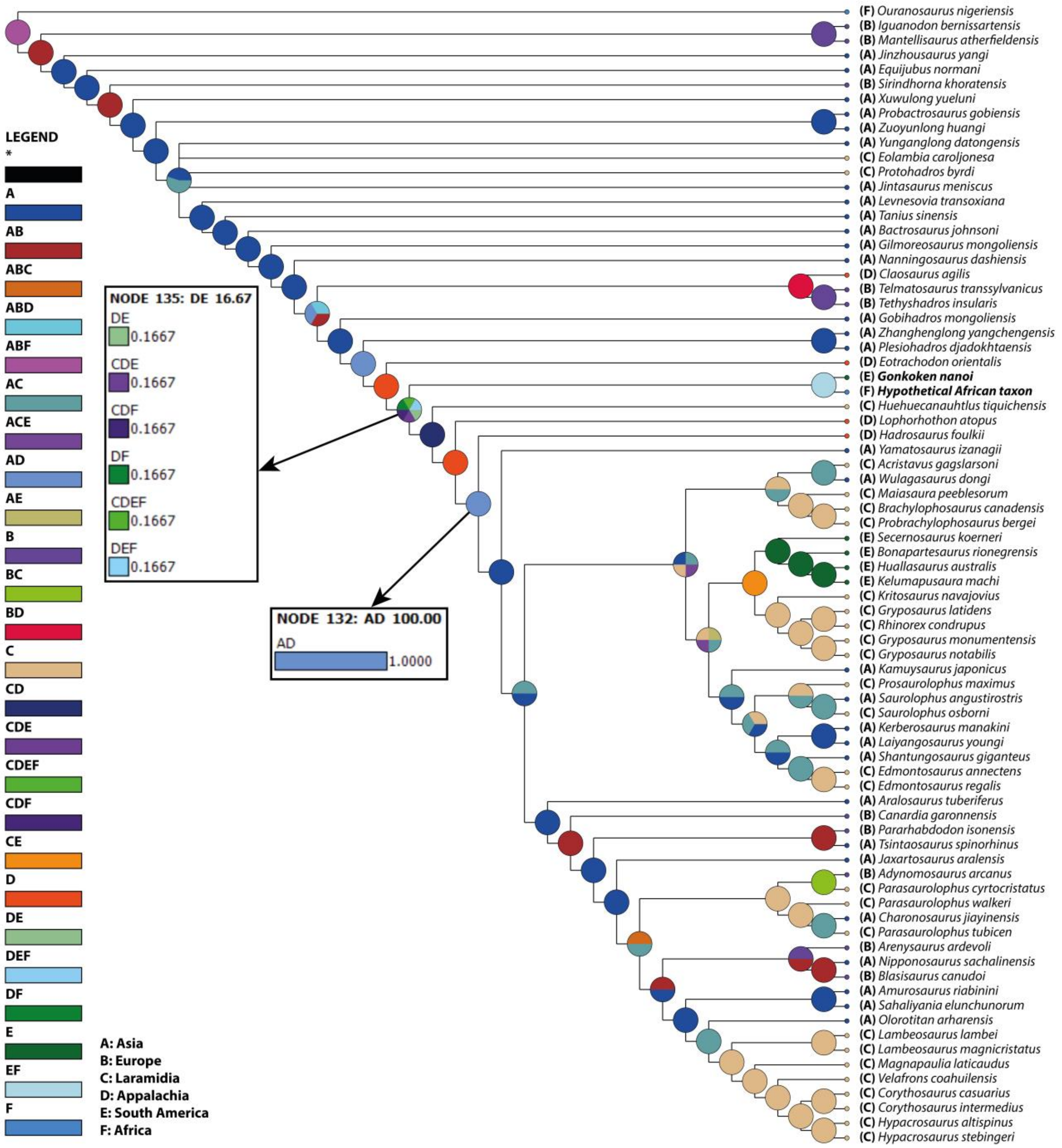

Fig. S16. Results of the s-DIVA with a "Hypothetical African taxon" as sister taxon of *Gonkoken*. Node 132: Hadrosauridae; Node 135: *Gonkoken* + *Hypothetical African taxon* + *Huehuecanauthlus* + *Lophorhothon* + Hadrosauridae.

Table S1. Representation of bones of *Gonkoken nanoi.* Distribution of skeletal regions and series.

**Representation by skeletal region**		
**Region**	**Element representation**	
Skull	9	20.00%
Vertebrae	13	28.89%
Pectoral girdle	2	4.44%
Ribs	4	8.89%
Forelimb	6	13.33%
Pelvic girdle	3	6.67%
Hindlimb	8	17.78%

Table S2. Voorhies groups. Relative frequency of elements according to Voorhies groups (based on 141). *Skull elements may be over-represented because of the disarticulated skull, nevertheless it does not change the group III predominance, even if a single skull element is considered).

**Voorhies groups**			
I	Caudal vertebrae	8	26.67%
	Cervical vertebrae	2	
	Ischium	1	
	Metatarsal	1	
II	Ulna	2	26.67%
	Radius	1	
	Fibula	1	
	Dorsal vertebrae	3	
	Sternum	1	
	Ribs	4	
III	Skull elements*	6	46.67%
	Dentaries	3	
	Humerus	3	
	Femur	4	
	Tibia	2	
	Scapula	1	
	Ilium	2	

Table S3. Summary of the results of phylogenetic analyses forcing *Gonkoken* into different positions. Number of trees, number of steps and differences in steps are indicated, in addition to Templeton Tests results to assess their significance.

	**Original position**	**As the most basal hadrosauroid**	**As Austrokritosauria**
N° Trees	4	24	914
N° Steps	1335	1381	1350
Difference in number of steps	0	46	15
Templeton Tests	N/A	significant	not significant
p-value	N/A	<0.01	>0.05

Table S4. Models and parameters for each of the analyses with BioGeoBEARS. Values of log-Likelihood (lnL), Dispersal (d), Extinction (e), Founder effect (j), Corrected Akaike Information Criterion (AICc), and AICc Weight (AICc wt) scores from each model implemented. The best model (DIVALIKE + j) was selected based on the lowest AICc value (and higher AICc + wt).

Model	lnL	d	e	j	AICc	AICc + wt
DEC	-135.85	2.05×10^{-2}	1.15×10^{-2}	0.00	275.86	4.14×10^{-17}
DEC + j	-99.38	1.53×10^{-3}	1.61×10^{-3}	0.14	205.08	9.72×10^{-2}
DIVALIKE	-138.24	2.92×10^{-2}	1.45×10^{-2}	0.00	280.64	3.79×10^{-18}
DIVALIKE + j	-97.23	1.42×10^{-3}	1.48×10^{-3}	0.17	**200.80**	**8.27×10^{-1}**
BAYAREALIKE	-168.43	2.43×10^{-2}	7.11×10^{-2}	0.00	341.02	2.93×10^{-31}
BAYAREALIKE + j	-99.62	1.08×10^{-3}	1.55×10^{-3}	0.17	205.58	7.57×10^{-2}

Table S5. Experiment 1, "Hypothetical African taxon" between *Eotrachodon* and *Gonkoken*. Values of log-Likelihood (lnL), Dispersal (d), Extinction (e), Founder effect (j), Corrected Akaike Information Criterion (AICc), and AICc Weight (AICc wt) scores from each model implemented. The best model (DIVALIKE + j) was selected based on the lowest AICc value (and higher AICc + wt).

Model	lnL	d	e	j	AICc	AICc + wt
DEC	-141.13	2.20×10^{-2}	1.43×10^{-2}	0.00	286.42	1.93×10^{-15}
DEC + j	-109.27	2.68×10^{-3}	3.45×10^{-3}	0.13	224.87	4.48×10^{-2}
DIVALIKE	-144.94	3.14×10^{-2}	2.07×10^{-2}	0.00	294.04	4.27×10^{-17}
DIVALIKE + j	-106.26	2.44×10^{-3}	2.55×10^{-3}	0.15	**218.83**	**9.16×10^{-1}**
BAYAREALIKE	-168.50	2.65×10^{-2}	7.25×10^{-2}	0.00	341.15	2.52×10^{-27}
BAYAREALIKE + j	-109.41	2.17×10^{-3}	3.13×10^{-3}	0.18	225.15	3.90×10^{-2}

Table S6. Experiment 2, "Hypothetical African taxon" as sister taxon of *Gonkoken*. Values of log-Likelihood (lnL), Dispersal (d), Extinction (e), Founder effect (j), Corrected Akaike Information Criterion (AICc), and AICc Weight (AICc wt) scores from each model implemented. The best model (BAYAREALIKE + j) was selected based on the lowest AICc value (and higher AICc + wt).

Model	lnL	d	e	j	AICc	AICc + wt
DEC	-139.57	2.18×10^{-2}	1.33×10^{-2}	0.00	283.30	1.12×10^{-16}
DEC + j	-102.75	1.11×10^{-3}	1.76×10^{-3}	0.13	211.83	3.71×10^{-1}
DIVALIKE	-143.90	3.10×10^{-2}	1.85×10^{-2}	0.00	291.96	1.48×10^{-18}
DIVALIKE + j	-105.09	2.62×10^{-3}	1.61×10^{-3}	0.08	216.50	3.58×10^{-2}
BAYAREALIKE	-168.48	2.65×10^{-2}	7.26×10^{-2}	0.00	341.11	3.13×10^{-29}
BAYAREALIKE + j	-102.28	1.07×10^{-3}	1.55×10^{-3}	0.18	**210.89**	**5.93×10^{-1}**

Table S7. FADs (First Appearance Data) and LADs (Last Appearance Data). The references used to obtain this information can be found as an auxiliary supplementary file.

Taxon	FAD	LAD
Ouranosaurus nigeriensis	129.4	100.5
Iguanodon bernissartensis	125.4	117.2
Mantellisaurus atherfieldensis	121.4	117.2
Jinzhousaurus yangi	127.4	122.0
Equijubus normani	113.0	110.5
Sirindhorna khoratensis	121.4	113.0
Xuwulong yueluni	121.4	100.5
Probactrosaurus gobiensis	129.4	100.5
Zuoyunlong huangi	100.5	93.9
Yunganglong dathongensis	100.5	93.9
Eolambia caroljonesa	98.46	98.32

Protohadros byrdi	100.5	93.9
Jintasaurus meniscus	113.0	100.5
Levnesovia transoxiana	91.85	89.5
Tanius sinensis	77.85	69.05
Bactrosaurus johnsoni	77.85	71.9
Gilmoreosaurus mongoliensis	77.85	71.9
Claosaurus agilis	88.05	85.8
Nanningosaurus dashiensis	100.5	66.0
Adynomosaurus arcanus	71.449	69.269
Telmatosaurus transsylvanicus	72.3	69.05
Tethyshadros insularis	81.5	80.5
Eotrachodon orientalis	84.95	83.4
Zhangenglong yangchengensis	86.8	83.4
Plesiohadros djadokhtaensis	77.85	71.9
Lophorhothon atopus	83.8	75.85
Hadrosaurus foulkii	80.5	78.5
Yamatosaurus izanagii	71.94	71.69
Acristavus gagslarsoni	80.6	79.8
Wulagasaurus dongi	72.3	68.0
Maiasaura peeblesorum	76.4	77.2
Brachylophosaurus canadensis	77.85	71.9
Probrachylophosaurus bergei	83.8	77.85
Kritosaurus navajovius	73.49	73.83
Huallasaurus australis	77.85	69.05
Gryposaurus latidens	81.08	80.6
Rhinorex condrupus	75.88	75.15

Gryposaurus monumentensis	77.85	71.9
Gryposaurus notabilis	76.5	74.5
Prosaurolophus maximus	77.03	75.46
Saurolophus angustirostris	77.85	69.05
Saurolophus osborni	72.3	69.05
Shantungosaurus giganteus	77.85	71.9
Edmontosaurus annectens	67.5	66.0
Edmontosaurus regalis	74.9	71.9
Kamuysaurus japonicus	72.3	69.05
Kerberosaurus manakini	69.05	66.0
Laiyangosaurus youngi	73.5	72.9
Aralosaurus tuberiferus	84.95	77.85
Canardia garonnensis	69.05	66.0
Nipponosaurus sachalinensis	84.95	77.85
Blasisaurus canudoi	69.05	66.0
Pararhabdodon isonensis	66.2	66.0
Tsintaosaurus spinorhinus	83.8	77.85
Jaxartosaurus aralensis	86.8	83.4
Parasaurolophus cyrtocristatus	76.439	74.893
Parasaurolophus walkeri	77.03	76.39
Charonosaurus jiayinensis	69.05	66.0
Parasaurolophus tubicen	77.83	73.49
Arenysaurus ardevoli	69.05	66.0
Olorotitan ararhensis	69.05	66.0
Amurosaurus riabinini	69.05	66.0
Sahaliyania elunchunorum	69.05	66.0

Lambeosaurus lambei	76.39	76.1
Lambeosaurus magnicristatus	77.8	72.1
Magnapaulia laticaudus	75.21	74.55
Velafrons coahuilensis	72.5	71.4
Corythosaurus casuarius	77.03	76.39
Corythosaurus intermedius	77.03	75.46
Hypacrosaurus altispinus	70.9	69.97
Hypacrosaurus stebingeri	79.7	72.1
Kelumapusaura machi	72.3	66.0
Bonapartesaurus rionegrensis	77.85	69.05
Secernosaurus koerneri	69.05	66.0
Gobihadros mongoliensis	100.5	83.4
Huehuecanauhtlus tiquichensis	86.8	83.4
Gonkoken nanoi	72.9	66.0

Table S8. Measurements of bones of *Gonkoken nanoi*. *Measurement based on preserved elements**.**

Specimen	Measured structure	Measurement (mm)
Premaxilla (CPAP 5337)	Maximum anteroposterior length of the pre-circummnarial area	98.2
	Maximum width	86.1
Maxilla (CPAP 5338)	Anteroposterior length	136.2*
	Maximum dorsoventral height	54.8
Maxilla (CPAP 5339)	Anteroposterior length	99.1*
Maxilla (CPAP 5340)	Anteroposterior length	162.1*
Postorbital (CPAP 5341)	Dorsoventral height	86.1
	Anteroposterior width of the base of the jugal process	56.3
Dentary (CPAP 5342)	Anteroposterior length of the alveolar surface	148.8*
	Anteroposterior width of the alveolar sulcus	~3.5
	Anteroposterior width of the coronoid process	36.6
Dentary (CPAP 5368)	Anteroposterior width of the alveolar sulcus	~5.4
Dentary (CPAP 5370)	Length of the proximal edentulous margin	34.5
	Anteroposterior length of the alveolar surface	149.3
	Length of the symphyseal process	68.2
	Anteroposterior width of the alveolar sulcus	~4.6
	Anteroposterior width of the coronoid process	33.1

Quadrate (CPAP 5343)	Dorsoventral height	168.9
	Mediolateral width of the distal end	50.5
Cervical centrum (CPAP 5380)	Anteroposterior length	43.0
	Mediolateral width	58.0
Cervical vertebra (CPAP 5344)	Anteroposterior length of the centrum	53.4
	Mediolateral width of the anterior face	53.8
	Mediolateral width of the posterior face	57.0
	Anteroposterior length of the postzygapophysis (r/l)	31.7*/35.5*
Dorsal centrum (CPAP 5345)	Anteroposterior length of the centrum	53.9
	Dorsoventral length of the centrum	39.5
Dorsal vertebra (CPAP 5346)	Anteroposterior length of the centrum	50.0
Dorsal arch (CPAP 5396)	Height preserved	72.4
	Dorsoventral height of the centrum	54.1
Proximal caudal centrum (CPAP 5397)	Anteroposterior length of the centrum	26.0
	Dorsoventral height of the centrum	58.0
Proximal caudal vertebra (CPAP 5398)	Anteroposterior length of the centrum	37.4
	Dorsoventral height of the centrum	56.3
Proximal caudal vertebra (CPAP 5399)	Dorsoventral height of the centrum	45.6
	Anteroposterior length of the centrum	40.1
Mid caudal vertebra (CPAP 5347)	Anteroposterior length of the centrum	43.3

	Dorsoventral height of the centrum	40.5
Mid caudal vertebra (CPAP 5348)	Anteroposterior length of the centrum	41.0
	Dorsoventral height of the centrum	43.7
Mid caudal vertebra (CPAP 5349)	Anteroposterior length of the centrum	39.3
	Dorsoventral height of the centrum	37.6
Mid caudal vertebra (CPAP 5350)	Anteroposterior length of the centrum	42.7
	Dorsoventral height of the centrum	44.7
Mid caudal vertebra (CPAP 5351)	Dorsoventral height of the centrum	43.9
	Anteroposterior length of the centrum	37.5
Sternum (CPAP 5352)	Preserved length	137.3
Scapula (CPAP 5371)	Total length	357.1
	Maximum dorsoventral height of the proximal end	104.3
	Dorsoventral height of the scapular blade in the midpoint	70.7
	Dorsoventral height of the distal end	89.7
Humerus (CPAP 5353)	Total length	306.8
	Length of the deltopectoral crest	134.4
	Dorsoventral width of the distal end	88.8
Humerus (CPAP 5354)	Total length	259.4*
	Length of the deltopectoral crest	129.1
Humerus (CPAP 5369)	Dorsoventral width of the distal end	78.7
Ulna (CPAP 5355)	Length	152.3*
Ulna (CPAP 5392)	Length	194.2*

Radius (CPAP 5379)	Mediolateral width of the proximal end	47.0
Ilium (CPAP 3054)	Length of the preacetabular process	134.2*
	Height of the base of the preacetabular process	5.7
	Anteroposterior length of the iliac blade	150.3
	Height of the iliac blade between the dorsal surface and the pubic process	96.4
	Anteroposterior length of the supraacetabular process	124.1
Incomplete ilium (CPAP 5356)	Anteroposterior length of the supraacetabular process	96.3
	anteroposterior length of the postacetabular process	97.8
Ischium (CPAP 5357)	Height of the iliac peduncle	67.1
	Height of the pubic peduncle	45.1
Femur (CPAP 5358)	Total length	485.1
	Mediolateral width of the distal end	70.5
Femur (CPAP 5359)	Total length	475.2
	Mediolateral width of the distal end	70.6
Femur (CPAP 5360)	Mediolateral width of the distal end	84.8
Femur (CPAP 5361)	Mediolateral width of the distal end	74.3
Tibia (CPAP 5362)	Total length	437
	Length of the cnemial crest	164.9
Tibia (CPAP 5372)	Total length	447.1*
Fibula (CPAP 5363)	Anteroposterior length of the proximal end	53.4*
Metatarsal III (CPAP 5364)	Length	169.2
	Mediolateral width of the shaft	41.4

Table S9. List of *Gonkoken nanoi* materials (CPAP) from the Río de las Chinas Valley. Some materials in preparation are included, which are represented in Fig. S1.

Specimen	Code	Observations
Incomplete right maxilla	CPAP 5340	this paper
Incomplete left maxilla of a juvenile individual	CPAP 5338	this paper
Incomplete left maxilla	CPAP 5339	this paper
Incomplete left postorbital	CPAP 5341	this paper
Incomplete right premaxilla	CPAP 5337	this paper
Left and right associated premaxillaries	LK-43	in preparation (see Fig. S1)
Incomplete right dentary	CPAP 5342	this paper
Fragmentary right dentary	CPAP 5368	this paper
Left dentary	CPAP 5370	this paper
Right quadrate	CPAP 5343	this paper
Cervical vertebra	CPAP 5344	this paper
Cervical centrum	CPAP 5380	this paper
Dorsal centrum	CPAP 5345	this paper
Dorsal vertebra	CPAP 5346	this paper
Dorsal neural arch	CPAP 5396	this paper
Mid caudal vertebra	CPAP 5347	this paper
Proximal caudal vertebra	CPAP 5397	this paper
Incomplete proximal caudal vertebra	CPAP 5398	this paper
Proximal caudal vertebra	CPAP 5399	this paper
Mid caudal vertebra	CPAP 5348	this paper
Mid caudal vertebra	CPAP 5349	this paper

Mid caudal vertebra	CPAP 5350	this paper
Mid caudal vertebra	CPAP 5351	this paper
Incomplete right rib	CPAP 5400	this paper
Incomplete right rib	CPAP 5401	this paper
Proximal portion of left rib	CPAP 5402	this paper
Incomplete left rib	CPAP 5403	this paper
Right Scapula	CPAP 5371	this paper
Left scapula	LK-3	in preparation (see Fig. S1)
Left scapula	LK-16	in preparation (see Fig. S1)
Incomplete left sternum	CPAP 5352	this paper
Left humerus	CPAP 5353	this paper
Incomplete left humerus	CPAP 5354	this paper
Left humerus	CPAP 5369	this paper
Complete left humerus of a subadult individual	LK-7	in preparation (see Fig. S1)
Incomplete right ulna	CPAP 5392	this paper
Left ulna	CPAP 5355	this paper
Right radius	CPAP 5379	this paper
Incomplete sacrum	LK-4	in preparation (see Fig. S1)
Incomplete right ilium	CPAP 3054	this paper
Left ilium fragment	CPAP 5336	this paper
Complete left ilium	LK-43	in preparation (see Fig. S1)
Incomplete left pubis	LK-6	in preparation (see Fig. S1)
Incomplete right ischium	CPAP 5357	this paper
Left femur	CPAP 5358	this paper
Incomplete right femur	CPAP 5359	this paper
Distal portion of a right femur	CPAP 5360	this paper

Distal fragment of a right femur (juvenile)	CPAP 5361	this paper
Right femur	LK-5	in preparation (see Fig. S1)
Left tibia	CPAP 5362	this paper
Incomplete left tibia	CPAP 5372	this paper
Incomplete left fibula	CPAP 5363	this paper
Right metatarsal III	CPAP 5364	this paper

Other Supplementary Materials for this manuscript includes the following:

Data file S1 (.nex format). Character-taxon data matrix.
Data file S2 (.OUT format). List of synapomorphies and characters supporting nodes.
Data file S3 (.R format). Script used for BioGeoBEARS.
Data file S4 (.txt). Dispersal matrix (hadrosauroids).
Data file S5 (.txt). Geographic data.
Data file S6 (.nex.trprobs). Posterior probabilities for trees_Undated Bayesian analysis.
Data file S7 (.nex.trprobs). Posterior probabilities for trees_Dated Bayesian analysis.
Data file S8 (.xlsx). Hadrosaur ages and references.

REFERENCES AND NOTES

1. D. B. Weishampel, Evolution of jaw mechanisms in ornithopod dinosaurs. *Adv. Anat. Embryol. Cell Biol.* **87**, 1–109 (1984).

2. F. L. Condamine, G. Guinot, M. J. Benton, P. J. Currie, Dinosaur biodiversity declined well before the asteroid impact, influenced by ecological and environmental pressures. *Nat. Commun.* **12**, 3833 (2021).

3. A. Prieto-Márquez, Global historical biogeography of hadrosaurid dinosaurs. *Zool. J. Linn.* **159**, 503–525 (2010).

4. S. Rozadilla, F. Brissón-Egli, F. L. Agnolín, A. M. Aranciaga-Rolando, F. E. Novas, A new hadrosaurid (Dinosauria: Ornithischia) from the Late Cretaceous of northern Patagonia and the radiation of South American hadrosauroids. *J. Syst. Palaeontol.* **19**, 1207–1235 (2022).

5. N. R. Longrich, X. Pereda Suberbiola, A. R. Pyron, J. Nour-Eddine, The first duckbill dinosaur (Hadrosauridae: Lambeosaurinae) from Africa and the role of oceanic dispersal in dinosaur biogeography. *Cretac. Res.* **120**, 104678 (2021).

6. M. K. Brett-Surman, Phylogeny and palaeobiogeography of hadrosaurian dinosaurs. *Nature* **277**, 560–562 (1979).

7. J. F. Bonaparte, M. R. Franchi, J. E. Powell, E. G. Sepúlveda, La Formación Alamitos (Campaniano-Maastrichtiano) del sudeste del Río Negro, con descripción de *Kritosaurus australis* n. sp. (Hadrosauridae). Significado paleogeográfico de los vertebrados. *Rev. Asoc. Geol. Argent.* **39**, 284–299 (1984).

8. R. A. Coria, B. González Riga, S. Casadío, Un nuevo hadrosáurido (Dinosauria, Ornithopoda) de la Formación Allen, Provincia de La Pampa, Argentina. *Ameghiniana* **49**, 552–572 (2012).

9. P. Cruzado-Caballero, J. Powell, *Bonapartesaurus rionegrensis*, a new hadrosaurine dinosaur from South America: Implications for phylogenetic and biogeographic relations with North America. *J. Vertebr. Paleontol.* **37**, e1289381 (2017).

10. M. A. Reguero, F. J. Goin, Paleogeography and biogeography of the Gondwanan final breakup and its terrestrial vertebrates: New insights from southern South America and the "double Noah's ark" Antarctic Peninsula. *J. South Am. Earth Sci.* **108**, 103358 (2021).

11. S. Rozadilla, F. Agnolín, M. Manabe, T. Tsuihiji, F. E. Novas, Ornithischian remains from the Chorrillo Formation (Upper Cretaceous), southern Patagonia, Argentina, and their implications on ornithischian paleobiogeography in the Southern Hemisphere. *Cretac. Res.* **125**, 104881 (2021).

12. T. H. Rich, P. Vickers-Rich, M. Fernández, S. Santillana, A probable hadrosaur from Seymour Island, Antarctic Peninsula, in *Proceedings of the Second Gondwanan Dinosaur Symposium*, Y. Tomida, T. H. Rich, P. Vickers-Rich, Eds. (National Science Museum, Tokyo, 1999, pp. 219–222.

13. J. A. Case, J. E. Martin, D. S. Chaney, M. Reguero, S. A. Marenssi, S. M. Santillana, M. O. Woodburne, The first duck-billed dinosaur (Family Hadrosauridae) from Antarctica. *J. Vertebr. Paleontol.* **20**, 612–614 (2000).

14. N. Malumián, C. Náñez, The Late Cretaceous–Cenozoic transgressions in Patagonia and the Fuegian Andes: Foraminifera, palaeoecology and palaeogeography. *Biol. J. Linn. Soc.* **103**, 269–288 (2011).

15. T. Jujihara, E. Frey, W. Stinnesbeck, P. Cruzado-Caballero, Á. Zúñiga-Reinoso, H. Mansilla, D. Rubilar-Rogers, J. Alarcón-Muñoz, M. Vogt, M. Leppe. Nuevo hadrosauroideo del Maasrichtiano inferior de la Formación Dorotea, Patagonia Austral Chilena. *Avances en Paleontología Chile, abstract I Congreso Chileno de Paleontología* 357–359 2018).

16. D. Madzia, V. M. Arbour, C. A. Boyd, A. A. Farke, P. Cruzado-Caballero, D. C. Evans, The phylogenetic nomenclature of ornithischian dinosaurs. *PeerJ* **9**, e12362 (2021).

17. M. K. Brett-Surman, J. R. Wagner, Discussion of character analysis of the appendicular anatomy in Campanian and Maastrichtian North American Hadrosaurids-variation and ontogeny, in *Horns and Beaks: Ceratopsian and Ornithopod Dinosaurs*, K. Carpenter, Ed. (Indiana Univ. Press, Bloomington, 2007), pp. 135–169.

18. D. W. E. Hone, A. Farke, M. J. Wedel, Ontogeny and the fossil record: What, if anything, is an adult dinosaur? *Biol. Lett.* **12**, 20150947 (2016).

19. B. Holland, P. R. Bell, F. Fanti, S. M. Hamilton, D. W. Larson, R. Sissons, C. Sullivan, M. J. Vavrek, Y. Wang, N. E. Campione, Taphonomy and taxonomy of a juvenile lambeosaurine (Ornithischia: Hadrosauridae) bonebed from the late Campanian Wapiti Formation of northwestern Alberta, Canada, *PeerJ* **9**, e11290 (2021).

20. A. Fernández Garay, *Diccionario Tehuelche-Español/Índice Español-Tehuelche*. Países Bajos: Escuela de Investigación de Estudios Asiáticos, Africanos y Amerindios (CNWS) (Universidad de Leiden, 2004).

21. T. M. Schwartz, J. C. Fosdick, S. A. Graham, Using detrital zircon U-Pb ages to calculate Late Cretaceous sedimentation rates in the Magallanes-Austral basin, Patagonia. *Basin Res.* **29**, 725–746 (2016).

22. N. M. Gutiérrez, J. P. Le Roux, A. Vásquez, C. Carreño, V. Pedroza, J. Araos, J. L. Oyarzún, J. P. Pino, H. A. Rivera, L. F. Hinojosa, Tectonic events reflected by palaeocurrents, zircon geochronology, and palaeobotany in the Sierra Baguales of Chilean Patagonia. *Tectonophysics* **695**, 76–99 (2017).

23. J. R. Horner, D. B. Weishampel, C. A. Foster, Hadrosauridae, in *The Dinosauria*, D. B. Weishampel, P. Dodson, H. Osmólska, Eds. (University of California Press, Berkeley, 2004), pp. 438–463.

24. A. Prieto-Márquez, G. M. Erickson, J. A. Ebersole, A primitive hadrosaurid from southeastern North America and the origin and early evolution of 'duck-billed' dinosaurs. *J. Vertebr. Paleontol.* **36**, e1054495 (2016).

25. P. Godefroit, Z.-M. Dong, P. Bultynck, H. Li, L. Feng, Sino-Belgian Cooperative Program. Cretaceous dinosaurs and mammals: 1) New *Bactrosaurus* (Dinosauria: Hadrosauroidea) material from Iren Dabasu (Inner Mongolia, P. R. China), *Bull. Inst. Roy. Sci. Natur. Belg.* **68**, 1–70 (1998).

26. F. M. Dalla Vecchia, *Tethyshadros insularis*, a new hadrosauroid dinosaur (Ornithischia) from the Upper Cretaceous of Italy. *J. Vertebr. Paleontol.* **29**, 1100–1116 (2009).

27. K. Tsogtbaatar, D. B. Weishampel, D. C. Evans, M. Watanabe, A new hadrosauroid (*Plesiohadros djadokhtaensis*) from the Late Cretaceous Djadokhtan fauna of southern Mongolia, in *Hadrosaurs*, D. Eberth, C. Evans, Eds. (Indiana Univ. Press, Bloomington, 2014), pp. 108–135.

28. A. T. McDonald, J. Bird, J. I. Kirkland, P. Dodson, Osteology of the basal hadrosauroid *Eolambia caroljonesa* (Dinosauria: Ornithopoda) from the Cedar Mountain Formation of Utah. *PLOS ONE* **7**, e45712 (2012).

29. A. Prieto-Márquez, Global phylogeny of Hadrosauridae (Dinosauria: Ornithopoda) using parsimony and Bayesian methods. *Zool. J. Linn. Soc.* **159**, 435–502 (2010).

30. N. E. Campione, K. S. Brink, E. A. Freedman, C. T. McGarrity, D. C. Evans, '*Glishades ericksoni*', an indeterminate juvenile hadrosaurid from Two Medicine Formation of Montana: Implications for hadrosauroid diversity in the latest Cretaceous (Campanian-Maastrichtian) of western North America. *Palaeobiodivers. Palaeoenviron.* **93**, 65–75 (2012).

31. K. Tsogtbaatar, D. B. Weishampel, D. C. Evans, M. Watabe, A new hadrosauroid (Dinosauria: Ornithopoda) from the Late Cretaceous Baynshire Formation of the Gobi Desert (Mongolia). *PLOS ONE* **14**, e0208480 (2019).

32. T. A. Gates, J. P. Lamb, A redescription of *Lophorhothon atopus* (Ornithopoda: Dinosauria) from the Late Cretaceous of Alabama based on new material. *Can. J. Earth Sci.* **58**, 918–935 (2021).

33. H.-D. Sues, A. Averianov, A new basal hadrosauroid dinosaur from the Late Cretaceous of Uzbekistan and the early radiation of duck-billed dinosaurs. *Proc. R. Soc. B*, **276**, 2549–2555 (2009).

34. A. Prieto-Márquez, G. M. Erickson, J. A. Ebersole, Anatomy and osteohistology of the basal hadrosaurid dinosaur *Eotrachodon* from the uppermost Santonian (Cretaceous) of southern Appalachia. *PeerJ* **4**, e1842 (2016).

35. A. Prieto-Márquez, M. A. Norell, Anatomy and relationships of *Gilmoreosaurus mongoliensis* (Dinosauria: Hadrosauroidea) from the Late Cretaceous of Central Asia. *Am. Mus. Novit.* **3694**, 1–49 (2010).

36. A. Prieto-Márquez, R. Gaete, G. Rivas, À. Galobart, M. Boada, Hadrosauroid dinosaurs from the Late Cretaceous of Spain: *Pararhabdodon isonensis* revisited and *Koutalisaurus kohlerorum*, gen. et sp. nov. *J. Vertebr. Paleontol.* **26**, 929–943 (2006).

37. A. Prieto-Márquez, "Phylogeny and historical biogeography of hadrosaurid dinosaurs," thesis, Florida State University (2008), pp. 936.

38. H. Xing, D. Wang, F. Han, C. Sullivan, Q. Ma, Y. He, D. W. E. Hone, R. Yan, F. Du, X. Xu, A new basal hadrosauroid dinosaur (Dinosauria: Ornithopoda) with transitional features from the Late Cretaceous of Henan Province, China. *PLOS ONE* **9**, e98821 (2014).

39. A. Prieto-Márquez, V. Fondevilla, A. G. Sellés, J. R. Wagner, À. Galobart, *Adynomosaurus arcanus*, a new lambeosaurine dinosaur from the Late Cretaceous Ibero-Armorican Island of the European archipelago. *Cretac. Res.* **96**, 19–37 (2019).

40. A. Prieto-Márquez, M. Á. Carrera Farias, The late-surviving early diverging Ibero-Armorican 'duck-billed' dinosaur Fylax and the role of the Late Cretaceous European Archipelago in hadrosauroid biogeography. *Acta Palaeontol. Pol.* **66**, 425–435 (2021).

41. A. Prieto-Márquez, Revised diagnoses of *Hadrosaurus foulkii* Leidy, 1858 (the type genus and species of Hadrosauridae Cope, 1869) and *Claosaurus agilis* Marsh, 1872 (Dinosauria: Ornithopoda) from the Late Cretaceous of North America. *Zootaxa* **2765**, 61–68 (2011).

42. A. Prieto-Márquez, D. B. Weishampel, J. R. Horner, The hadrosaurid dinosaur *Hadrosaurus foulkii* from the Campanian of the East coast of North America, with a review of the genus. *Acta Palaeontol. Pol.* **51**, 77–98 (2006).

43. A. A. Ramírez-Velasco, M. Benammi, A. Prieto-Márquez, J. A. Ortega, R. Hernández-Rivera, *Huehuecanauhtlus tiquichensis*, a new hadrosauroid dinosaur (Ornithischia: Ornithopoda) from the Santonian (Late Cretaceous) of Michoacán, Mexico. *Can. J. Earth Sci.* **49**, 379–395 (2012).

44. D. B. Norman, On the anatomy of *Iguanodon atherfieldensis* (Ornithischia: Ornithopoda). *Bull. Inst. R. Sci. Nat. Belg.* **56,** 281–372 (1986).

45. R.-F. Wang, H.-L. You, S.-C. Xu, S.-Z. Wang, J. Yi, L.-J. Xie, L. Jia, Y.-X. Li, A new hadrosauroid dinosaur from the Early Late Cretaceous of Shanxi Province, China. *PLOS ONE* **8**, e77058 (2013).

46. T. Qing-Wei, X. Hai, H. Yong-Guo, T. Lin, X. Xing, New hadrosauroid material from the Upper Cretaceous Majiacun Formation of Hubei Province, central China. *Vertebr. PalAs.* **53**, 245–264 (2015).

47. A. Prieto-Márquez, Skeletal morphology of *Kritosaurus navajovius* (Dinosauria: Hadrosauridae) from the Late Cretaceous of the North American south-west, with an evaluation of the phylogenetic systematics and biogeography of Kritosaurini. *J. Syst. Palaeontol.* **12**, 133–175 (2014).

48. A. A. Chiarenza, M. Fabbri, L. Consorti, M. Muscioni, D. C. Evans, J. L. Cantalapiedra, F. Fanti. An Italian dinosaur Lagerstätte reveals the tempo and mode of hadrosauriform body size evolution. *Sci. Rep.* **11**, 23295 (2021).

49. M. C. Lamanna, J. A. Case, E. M. Roberts, V. M. Arbour, R. C. Ely, S. W. Salisbury, J. A. Clarke, D. E. Malinzak, A. R. West, P. M. O'Connor, Late Cretaceous non-avian dinosaurs from the James Ross Basin, Antarctica: Description of new material, updated synthesis, biostratigraphy, and paleobiogeography. *Adv. Polar Sci.* **30**, 228–250 (2019).

50. M. C. Lamanna, G. A. Casal, L. M. Ibiricu, R. D. F. Martínez, Non-Avian dinosaur faunas of the Upper Cretaceous Bajo Barreal and Lago Colhué Huapi formations of Central Patagonia, Argentina: Paleobiogeographic and Biostratigraphic implications. XXI Congreso Geológico Argentino, Technical Session VI, Paleontología, Paleoecología y Bioestratigrafía, Puerto Madryn, Chubut, 259–260 (March 2022).

51. C. R. Scotese, Atlas of Late Cretaceous Maps, PALEOMAP Atlas for ArcGIS, volume 2, The Cretaceous, Maps 16–22, Mollweide Projection, PALEOMAP Project, Evanston, IL (2014).

52. F. J. Goin, J. N. Gelfo, L. Chornogubsky, M. O. Woodburne, T. Martin, Origins, radiations, and distribution of South American mammals: From greenhouse to icehouse worlds, in *Bones, Clones, and Biomes: The History and Geography of Recent Neotropical Mammals*, B. D. Patterson, L. P. Costa, Eds. (University of Chicago Press, Chicago, 2012), pp. 20–50.

53. F. J. Goin, M. O. Woodburne, A. N. Zimicz, G. M. Martin, L. Chornogubsky, Dispersal of vertebrates from between the Americas, Antarctica, and Australia in the Late Cretaceous and Early Cenozoic, in *A Brief History of South American Metatherians* (Springer, Dordrecht, 2016), pp. 77–124.

54. L. M. Carneiro, É. V. Oliveira, F. J. Goin, *Austropediomys marshalli* gen. et sp. nov., a new Pediomyoidea (Mammalia, Metatheria) from the Paleogene of Brazil: Paleobiogeographic implications. *Rev. Bras. Paleontol.* **21**, 120–131 (2018).

55. F. Riguetti, X. Pereda-Suberbiola, D. Ponce, L. Salgado, S. Apesteguía, S. Rozadilla, V. Arbour, A new small-bodied ankylosaurian dinosaur from the Upper Cretaceous of North Patagonia (Río Negro Province, Argentina). *J. Syst. Palaeontol.* **20**, 2137441 (2022).

56. R. S. Tykoski, A. R. Fiorillo, An articulated cervical series of *Alamosaurus sanjuanensis* Gilmore, 1922 (Dinosauria, Sauropoda) from Texas: New perspective on the relationships of North America's last giant sauropod. *J. Syst. Palaeontol.* **15**, 339–364 (2017).

57. M. D. Ezcurra, F. L. Agnolín, A new Palaeobiogeographical Model for the Late Mesozoic and Early Tertiary. *Sys. Biol.* **64**, 553–566 (2012).

58. B. Vila, A. Sellés, M. Moreno-Azanza, N. L. Razzolini, A. Gil-Delgado, J. I. Canudo, À. Golobart. A titanosaurian sauropod with Gondwanan affinities in the latest Cretaceous of Europe. *Nat. Ecol. Evol.* **6**, 288–296 (2022).

59. N. J. Matzke, BioGeoBEARS: BioGeography with Bayesian (and likelihood) Evolutionary Analysis with R Scripts. version 1.1.1, published on GitHub on November 6, 2018; http://dx.doi.org/10.5281/zenodo.1478250.

60. F. Ronquist, Dispersal-Vicariance analysis: A new approach to the quantification of historical biogeography. *Syst. Biol.* **46**, 195–203 (1997).

61. Y. Yu, A. J. Harris, X. He, S-DIVA (Statistical Dispersal-Vicariance Analysis): A tool for inferring biogeographic histories. *Mol. Phyl. Evo.* **56**, 848–850 (2010).

62. Y. Yu, A. J. Harris, C. Blair, X. He, RASP (Reconstruct Ancestral State in Phylogenies): A tool for historical biogeography. *Mol. Phyl. Evo.* **87**, 46–49 (2015).

63. O. Mateus, Cretaceous amniotes from Angola: Dinosaurs, pterosaurs, mosasaurs, plesiosaurs, turtles. *V Jornadas Internacionales sobre Paleontología de Dinosaurios y su Entorno, Salas de los Infantes, Burgos*, 71–105 (2012).

64. E. Buffetaut, A.-F. Hartman, M. Al-Kindi, A. S. Schulp, Hadrosauroid dinosaurs from the Late Cretaceous of the Sultanate of Oman. *PLOS ONE* **10**, e0142692 (2015).

65. A. Ding, M. Pittman, P. Upchurch, J. O'Connor, D. J. Field, X. Xu, The biogeography of coelurosaurian theropods and its impact on their evolutionary history. *Bull. Am. Mus. Nat. Hist.* **440**, 117–157 (2020).

66. S. Soto-Acuña, A. O. Vargas, J. Kaluza, M. A. Leppe, J. F. Botelho, J. Palma-Liberona, C. Simon-Gutstein, R. A. Fernández, H. Ortiz, V. Milla, B. Aravena, L. M. E. Manríquez, J. Alarcón-Muñoz, J. P. Pino, C. Trevisan, H. Mansilla, L. F. Hinojosa, V. Muñoz-Walther, D. Rubilar-Rogers, Bizarre tail weaponry in a transitional ankylosaur from subantarctic Chile. *Nature* **600**, 259–263 (2021).

67. D. C. Evans, P. M. Barrett, K. L. Seymour, Revised identification of a reported *Iguanodon*-grade ornithopod tooth from the Scollard Formation, Alberta, Canada. *Cretac. Res.* **33**, 11–14 (2011).

68. G. C. Salinas, R. D. Juárez Valieri, L. E. Fiorelli, Comparación de los hadrosaurios de América del Sur y Europa. Abstracts 9° Congreso Argentino de Paleontología y Bioestratigrafía (Córdoba, 2006), pp. 89.

69. N. H. Borinder, S. F. Poropat, N. E. Campione, T. Wigren, B. P. Kear, Postcranial osteology of the basally branching hadrosauroid dinosaur *Tanius sinensis* from the Upper Cretaceous Wangshi Group of Shandong, China. *J. Vertebr. Paleontol.* **41**, e1914642 (2021).

70. J. García-Girón, A. A. Chiarenza, J. Alahuhta, D. G. DeMar Jr., J. Heino, P. D. Mannion, T. E. Williamson, G. P. Willson Mantilla, S. L. Brusatte, Shifts in food webs and niche stability shaped survivorship and extinction at the end-Cretaceous. *Sci. Adv.* **8**, eadd5040 (2022).

71. A. A. Chiarenza, P. D. Mannion, D. J. Lunt, A. Farnsworth, L. A. Jones, S.-J. Kelland, P. A. Allison, Ecological niche modelling does not support climatically-driven dinosaur diversity decline before the Cretaceous/Paleogene mass extinction. *Nat. Commun.* **10**, 1091 (2019).

72. R. E. Molnar, J. Wiffen, A presumed titanosaurian vertebra from the Late Cretaceous of North Island, New Zealand, *Arch. Mus. Nac. (Rio de J.)*, **65**, 505–510 (2007).

73. S. N. Davis, S. Soto-Acuña, R. A. Fernández, J. Amudeo-Plaza, M. A. Leppe, D. Rubilar-Rogers, A. O. Vargas, J. A. Clarke, New records of Theropoda from a Late Cretaceous (Campanian-Maastrichtian) locality in the Magallanes-Austral Basin, Patagonia, and insights into end Cretaceous theropod diversity. *J. South Am. Earth Sci.* **122**, 104163 (2023).

74. S. W. M. George, S. N. Davis, R. A. Fernández, L. M. E. Manríquez, M. A. Leppe, B. K. Horton, J. A. Clarke. Chronology of deposition and unconformity development across the Cretaceous–Paleogene boundary, Magallanes-Austral Basin, Patagonian Andes. *J. South Am. Earth Sci.* **97**, 102237 (2020).

75. K. T. Biddle, M. A. Uliana, R. M. Mitchum Jr., M. G. Fitzgerald, R. C. Wright, The stratigraphic and structural evolution of the central and eastern Magallanes Basin, southern South America, in *Foreland Basins*, P. A. Allen, P. Homewood, Eds. (Special Publication of the International Association of Sedimentologists 8, 1986), pp. 41–61.

76. B. G. Daniels, N. C. Auchter, S. M. Hubbard, B. W. Romans, W. A. Matthews, L. Stright, Timing of deep-water slope evolution constrained by large-*n* detrital and volcanic ash zircon geochronology, Cretaceous Magallanes Basin, Chile. *Geol. Soc. Am. Bull.* **130**, 438–454 (2018).

77. N. Malumián, A. Caramés, Upper Campanian-Paleogene from the Río Turbio coal measures in southern Argentina: Micropaleontology and the Paleocene/Eocene boundary. *J. South Am. Earth Sci.* **10**, 189–201 (1997).

78. H. H. Camacho, J. O. Chiesa, S. G. Parma, Relaciones estratigráficas entre formaciones terciarias en el occidente de la Provincia de Santa Cruz. *Rev. Asoc. Geol. Argent.* **53**, 273–281 (1998).

79. L. M. E. Manríquez, E. L. Lavina, R. A. Fernández, C. Trevisan, M. A. Leppe, Campanian-Maastrichtian and Eocene stratigraphic architecture, facies analysis, and paleoenvironmental evolution of the northern Magallanes Basin (Chilean Patagonia). *J. South Am. Earth Sci.* **93**, 102–118 (2019).

80. L. M. E. Manríquez, E. L. Lavina, R. G. Netto, R. S. Horodyski, M. Leppe, M. Evolution of a high latitude high-energy beach system (Maastrichtian–Eocene, Magallanes/Austral Basin, Chilean Patagonia). *Sediment. Geol.* **426**, 106026 (2021).

81. H. A. Rivera, J. P. Le Roux, M. Farías, N. M. Gutiérrez, A. Sánchez, S. Palma-Heldt, Tectonic controls on the Maastrichtian-Danian transgression in the Magallanes-Austral foreland basin (Chile): Implications for the growth of the Southern Patagonian Andes. *Sediment. Geol.* **403**, 105645 (2020).

82. T. M. Schwartz, S. A. Graham, Stratigraphic architecture of a tide-influenced shelf-edge delta, Upper Cretaceous Dorotea Formation, Magallanes-Austral Basin, Patagonia. *Sedimentology* **62**, 1039–1077 (2015).

83. Y. Kobayashi, T. Nishimura, R. Takasaki, K. Chiba, A. R. Fiorillo, K. Tanaka, T. Chinzoring, T. Sato, K. Sakurai, A new hadrosaurine (Dinosauria: Hadrosauridae) from the marine deposits of the Late Cretaceous Hakobuchi Formation, Yezo Group, Japan. *Sci. Rep.* **9**, 12389 (2019).

84. R. Takasaki, A. R. Fiorillo, R. S. Tykoski, Y. Kobayashi, Re-examination of the cranial osteology of the Arctic Alaskan hadrosaurine with implications for its taxonomic status. *PLOS ONE* **15**, e0232410 (2020).

85. W. P. Maddison, D. R. Maddison, Mesquite: A modular system for evolutionary analysis. Version 3.70 (2021); www.mesquiteproject.org.

86. P. A. Goloboff, M. E. Morales, TNT version 1.6, with a graphical interface for MacOS and Linux, including new routines in parallel. *Cladistics* **39**, 144–153 (2023).

87. A. R. Templeton, Phylogenetic inference from restriction endonuclease cleavage site maps with particular reference to the evolution of humans and the apes. *Evolution* **37**, 221–244 (1983).

88. A. N. Schmidt-Lebuhn, TNT script for the Templeton Test (2016); DOI:10.13140/RG.2.1.3484.9522.

89. D. W. Bapst, paleotree: An R package for paleontological and phylogenetic analyses of evolution. *Methods Ecol. Evol.* **3**, 803–807 (2012).

90. F. Ronquist, M. Teslenko, P. van der Mark, D. L. Ayres, A. Darling, S. Höhna, B. Larget, L. Liu, M. A. Suchard, J. P. Huelsenbeck, MrBayes 3.2: Efficient Bayesian phylogenetic inference and model choice across a large model space. *Syst. Biol.* **61**, 539–542 (2012).

91. P. Lewis, A likelihood approach to estimating phylogeny from discrete morphological character data. *Soc. Syst. Biol.* **50**, 913–925 (2001).

92. H. P. Püschel, J. E. O'Reilly, D. Pisani, P. C. J. Donoghue, The impact of fossil stratigraphic ranges on tip-calibration, and the accuracy and precision of divergence time estimates. *Palaeontology* **63**, 67–83 (2020).

93. E. Paradis, J. Claude, K. Strimmer, APE: Analyses of phylogenetics and evolution in R language. *Bioinformatics* **20**, 289–290 (2004).

94. M. L. Delignette-Muller, C. Dutang, fitdistrplus: An R package for fitting Ddistributions. *J. Stat. Softw.*, **64**, 1–34 (2015).

95. R. Core Team, *R: A Language and Environment for Statistical Computing* (R Foundation for Statistical Computing, 2019).

96. G. F. Gunnell, D. M. Boyer, A. R. Friscia, S. Heritage, F. K. Manthi, E. R. Miller, H. M. Sallam, N. B. Simmons, N. J. Stevens, E. R. Seiffert, Fossil lemurs from Egypt and Kenya suggest an African origin for Madagascar's aye-aye. *Nat. Commun.* **9**, 3193 (2018).

97. A. J. Harris, Q.-Y. Xiang, Estimating ancestral distributions of lineages with uncertain sister groups: A statistical approach to dispersal-vicariance analysis and a case using *Aesculus* L. (Sapindaceae) including fossils. *J. Syst. Palaeontol.* **47**, 349–368 (2009).

98. N. J. Matzke, Model selection in historical biogeography reveals that founder-event speciation is a crucial process in island clades. *Syst. Biol.* **63,** 951–970 (2014).

99. J. E. O'Reilly, P. C. Donoghue, The efficacy of consensus tree methods for summarizing phylogenetic relationships from a posterior sample of trees estimated from morphological data. *Syst. Biol.* **67**, 354–362 (2018).

100. R. H. Ree, S. A. Smith, Maximum likelihood inference of geographic range evolution by dispersal, local extinction, and cladogenesis. *Syst. Biol.* **57**, 4–14 (2008).

101. M. J. Landis, N. J. Matzke, B. R. Moore, J. P. Huelsenbeck, Bayesian analysis of biogeography when the number of areas is large. *Syst. Biol.* **62**, 789–804 (2013).

102. A. K. Behrensmeyer, Terrestrial vertebrate accumulations, in *Taphonomy: Releasing the Data Locked in the Fossil Record*, A. Allison, D. E. G. Briggs, Eds. (Plenum Press, New York, 1991), pp. 291–335.

103. R. R. Rogers, S. M. Kidwell, A conceptual framework for the genesis and analysis of vertebrate skeletal concentrations, in *Bonebeds: Genesis, Analysis, and Paleobiological Significance*, R. R. Rogers, D. A. Eberth, A. R. Fiorillo, Eds. (University of Chicago Press, Chicago, 2007), pp. 1–64.

104. M. R. Voorhies, Taphonomy and Population Dynamics of an Early Pliocene Vertebrate Fauna, Knox County, Nebraska (Contributions to Geology, Special Paper, 2, University of Wyoming Press: Laramie), pp. 1–69.

105. A. K. Behrensmeyer, The taphonomy and paleoecology of Plio-Pleistocene vertebrate assemblages east of Lake Rudolf, Kenya. *Bull. Mus. Comp. Zool.* **146**, 473–578 (1975).

106. M. J. Ryan, A. P. Russell, D. A. Eberth, P. J. Currie, The Taphonomy of a *Centrosaurus* (Ornithischia: Certopsidae) bone bed from the Dinosaur Park Formation (upper Campanian), Alberta, Canada, with comments on cranial ontogeny. *PALAIOS* **16**, 482–506 (2001).

107. P. Bell, N. E. Campione, Taphonomy of the Danek Bonebed: A monodominant *Edmontosaurus* (Hadrosauridae) bonebed from the Horseshoe Canyon Formation, Alberta. *Can. J. Earth Sci.*, **51**, 1–15 (2014).

108. K. Loughney, C. Badgley, The influence of depositional environment and basin history on the taphonomy of mammalian assemblages from The Barstow Formation (middle Miocene), California. *PALAIOS* **35**, 175–190 (2020).

109. A. R. Fiorillo, Taphonomy of Hazard Homestead Quarry (Ogallala Group), Hitchcock County, Nebraska. *Contrib. Geol. Univ. Wyo.* **26**, 57–97 (1988).

110. A. Prieto-Márquez, G. C. Salinas, A re-evaluation of Secernosaurus koerneriand Kritosaurus australis (Dinosauria, Hadrosauridae) from the Late Cretaceous of Argentina. *J. Vertebr. Paleontol.* **30**, 813–837 (2010).

111. J. Zhang, X. Wang, Q. Wang, S. Jiang, X. Cheng, R. Qiu, A new saurolophine hadrosaurid (Dinosauria: Ornithopoda) from the Upper Cretaceous of Shandong, China. *An. Acad. Bras. Ciênc.* **91**, e20160920 (2017).

112. D. C. Evans, R. R. Reisz, Anatomy and relationships *of Lambeosaurus magnicristatus*, a crested hadrosaurid dinosaur (Ornithischia) from The Dinosaur Park Formation, Alberta. *J. Vertebr. Paleontol.* **27**, 373–393 (2007).

113. D. B. Weishampel, D. B. Norman, D. Grigorescu, *Telmatosaurus transsylvanicus* from the Late Cretaceous of Romania: The most basal hadrosaurid dinosaur. *Palaeontology* **36**, 361–385 (1993).

114. N. R. Longrich, A ceratopsian dinosaur from the Late Cretaceous of eastern North America, and implications for dinosaur biogeography. *Cretac. Res.* **57**, 199–207 (2016).

115. F. Bertozzo, F. M. Dalla Vecchia, M. Fabbri, The Venice specimen of *Ouranosaurus nigeriensis* (Dinosauria, Ornithopoda). *PeerJ* **5**, e3403 (2017).

116. G. Paul, A revised taxonomy of the iguanodont dinosaur genera and species. *Cretac. Res.* **29**, 192–216 (2008).

117. J. Yans, J. Dejax, D. Pons, C. Dupuis, P. Taquet, Implications paléontologiques et géodynamiques de la datation palynologique des sédiments à faciès wealdien de Bernissart (bassin de Mons, Belgique). *C. R. Palevol* **4**, 135–150 (2005).

118. G. Paul, Turning the old into the new: A separate genus for the gracile iguanodont from the Wealden of England, in *Horns and Beaks: Ceratopsian and Ornithopod Dinosaurs,* K. Carpenter, Ed. (Indiana Univ. Press, Bloomington, 2006), pp. 69–77.

119. W. Yang, S. Li, B. Jiang, New evidence for Cretaceous age of the feathered dinosaurs of Liaoning: Zircon U-Pb SHRIMP dating of the Yixian Formation in Sihetun, northeast China, *Cretac. Res.* **28**, 177–182 (2007).

120. P. M. Barrett, R. J. Butler, X.-L. Wang, X. Xu, Cranial anatomy of the iguanodontoid ornithopod *Jinzhousaurus yangi* from the Lower Cretaceous Yixian Formation of China. *Acta Palaeontol. Pol.* **54**, 35–48 (2009).

121. F. Tang, Z.-X. Luo, Z.-H. Zhou, H.-L. You, J. A. Georgi, Z.-L. Tang, X.-Z. Wang, Biostratigraphy and palaeoenvironment of the dinosaur-bearing sediments in Lower Cretaceous of Mazongshan area, Gansu Province, China. *Cretac. Res.* **22**, 115–129 (2001).

122. H.-L. You, Z.-X. Luo, N. H. Shubin, L. M. Witmer, Z.-L. Tang, F. Tang, The earliest-known duck-billed dinosaur from deposits of late Early Cretaceous age in northwest China and hadrosaur evolution. *Cretac. Res.* **24**, 347–355 (2003).

123. A. T. McDonald, S. C. R. Maidment, P. M. Barrett, H. You, P. Dodson, Osteology of the basal hadrosauroid *Equijubus normani* (Dinosauria, Ornithopoda) from the Early Cretaceous of China, in *Hadrosaurs*, D. Eberth, C. Evans, Eds. (Indiana Univ. Press, Bloomington, 2014), pp. 44–72.

124. M. Shibata, P. Jintasakul, Y. Azuma, H.-L. You, A new basal hadrosauroid dinosaur from the Lower Cretaceous Khok Kruat Formation in Nakhon Ratchasima Province, Northeastern Thailand. *PLOS ONE* **10**, e0145904 (2015).

125. H. You, D. Li, W. Liu, A new Hadrosauriform Dinosaur from the Early Cretaceous of Gansu Province, China. *Acta Geol. Sin.* **85**, 51–57 (2011).

126. V. J. Itterbeeck, V. S. Markevich, D. J. Hone, The age of the dinosaur-bearing Cretaceous sediments at Dashuiguo, Inner Mongolia, P.R. China based on charophytes, ostracods and palynomorphs. *Cretac. Res.* **25**, 391–409 (2004).

127. A. K. Rozhdestvensky, New Iguanodonts from Central Asia. Phylogenetic and taxonomic interrelationships of late Iguanodontidae and early Hadrosauridae. *Palaeontol. Zhurnal.* **1966**, 103–116 (1966).

128. D. B. Norman, On Asian ornithopods (Dinosauria: Ornithischia). 4. Probactrosaurus Rozhdestvensky, 1966. *Zool. J. Linn. Soc.* **136**, 113–144 (2002).

129. R.-F. Wang, H.-L. You, S.-Z. Wang, S.-C. Xu, J. Yi, L.-J. Xie, L. Jia, H. Xing, A second hadrosauroid dinosaur from the early Late Cretaceous of Zuoyun, Shanxi Province, China. *Hist. Biol.* **29**, 17–24 (2017).

130. R. L. Cifelli, J. I. Kirkland, A. Weil, A. L. Deino, B. J. Kowallis, High-precision $^{40}Ar/^{39}Ar$ geochronology and the advent of North America’s Late Cretaceous terrestrial fauna. *Proc. Natl. Acad. Sci. U.S.A.* **94**, 11163–11167 (1997).

131. J. J. Head, A new species of basal hadrosaurid (Dinosauria, Ornithischia) from the Cenomanian of Texas. *J. Vertebr. Paleontol.* **18**, 718–738 (1998).

132. H.-L. You, D. Q. Li, A new basal hadrosauriform dinosaur (Ornithischia: Iguanodontia) from the Early Cretaceous of northwestern China. *Can. J. Earth Sci.* **46**, 949–957 (2009).

133. H. Xing, Y. He, L. Li, D. Xi, A review of the study of the stratigraphy, sedimentology, and paleontology of the Iren Dabasu Formation, Inner Mongolia, in *Proceedings of the Thirteenth Annual Meeting of the Chinese Society of Vertebrate Paleontology*, D. Wei, Ed. (China Ocean Press, Beijing, 2012), pp. 1–44.

134. V. J. Itterbeeck, D. J. Horne, P. Bultynck, N. Vandenberghe, Stratigraphy and palaeoenvironment of the dinosaur-bearing Upper Cretaceous Iren Dabasu Formation, Inner Mongolia, People’s Republic of China. *Cretac. Res.* **26**, 699–725 (2005).

135. J. Mo, Z. Zhao, W. Wang, X. Xu, The first hadrosaurid dinosaur from southern China. *Acta Geol. Sin.*, **81**, 550–554 (2007).

136. V. Fondevilla, V. Riera, B. Vila, A. G. Sellés, J. Dinarès-Turell, E. Vicens, R. Gaete, O. Oms, À. Galobart, Chronostratigraphic synthesis of the latest Cretaceous dinosaur turnover in south-western Europe. *Earth Sci. Rev.* **191**, 168–189 (2019).

137. A. V. Bojar, S. Halas, H.-P. Bojar, D. Grigorescu, Ş. Vasile, Upper Cretaceous volcanoclastic deposits from the Haţeg Basin, South Carpathians (Romania): K-Ar ages and intrabasinal correlation. *Geochronometria* **38**, 182–188 (2011).

138. W. B. Gallagher, Recent mosasaur discoveries from New Jersey and Delaware, USA: Stratigraphy, taphonomy and implications for mosasaur extinction. *Neth. J. Geosci.* **84**, 241–245 (2005).

139. Y. Kobayashi, R. Takasaki, K. Kubota, A. R. Fiorillo, A new basal hadrosaurid (Dinosauria: Ornithischia) from the latest Cretaceous Kita-ama Formation in Japan implies the origin of hadrosaurids. *Sci. Rep.* **11**, 8547 (2021).

140. T. A. Gates, J. R. Horner, R. R. Hanna, C. R. Nelson, New unadorned hadrosaurine hadrosaurid (Dinosauria, Ornithopoda) from the Campanian of North America. *J. Vertebr. Paleontol.* **31**, 798–811 (2011).

141. D. W. Fowler, Revised geochronology, correlation, and dinosaur stratigraphic ranges of the Santonian-Maastrichtian (Late Cretaceous) formations of the Western Interior of North America. *PLOS ONE* **12**, e0188426 (2017).

142. P. Godefroit, H. Shulin, Y. Tingxiang, P. Lauters, New hadrosaurid dinosaurs from the uppermost Cretaceous of northeastern China. *Acta Palaeontol. Pol.* **53**, 47–74 (2008).

143. E. A. Freedman Fowler, J. R. Horner, A new brachylophosaurin hadrosaur (Dinosauria: Ornithischia) with an intermediate nasal crest from the Campanian Judith River Formation of Northcentral Montana. *PLOS ONE* **10**, e0141304 (2015).

144. L. M. Ibiricu, G. A. Casal, B. N. Alvarez, A. De Sosa Tomas, M. C. Lamanna, P. Cruzado-Caballero, New hadrosaurid (Dinosauria: Ornithopoda) fossils from the uppermost Cretaceous of central Patagonia and the influence of paleoenvironment on South American hadrosaur distribution. *J. South Am. Earth Sci.* **110**, 103369 (2021).

145. F. Bertozzo, C. Dal Sasso, M. Fabbri, F. Manucci, S. Maganuco, Redescription of a remarkably large *Gryposaurus notabilis* (Dinosauria: Hadrosauridae) from Alberta, Canada. *Mem. Soc. ital. sci. nat. Mus. civ. stor. nat. Milano* **43**, 1–56 (2017).

146. T. A. Gates, R. Scheetz, A new saurolophine hadrosaurid (Dinosauria: Ornithopoda) from the Campanian of Utah, North America, *J. Syst. Palaeontol.* **13**, 711–725 (2015).

147. T. A. Gates, S. Sampson, A new species of *Gryposaurus* (Dinosauria: Hadrosauridae) from the late Campanian Kaiparowits Formation, southern Utah, USA. *Zool. J. Linn. Soc.* **151**, 351–376 (2007).

148. P. R. Bell, Cranial osteology and ontogeny of *Saurolophus angustirostris* from the Late Cretaceous of Mongolia with comments on *Saurolophus osborni* from Canada. *Acta Palaeontol. Pol.* **56**, 703–722 (2011).

149. P. R. Bell, Redescription of the skull of *Saurolophus osborni* Brown 1912 (Ornithischia: Hadrosauridae). *Cretac. Res.* **32**, 30–44 (2011).

150. H. Xing, X. Zhao, K. Wang, D. Li, S. Chen, J. C. Mallon, Y. Zhang, X. Xu, Comparative osteology and phylogenetic relationship of *Edmontosaurus* and *Shantungosaurus*(Dinosauria: Hadrosauridae) from the Upper Cretaceous of North America and East Asia. *Acta Geol. Sin.* **88**, 1623–1652 (2014).

151. N. E. Campione, D. C. Evans, Cranial growth and variation in *Edmontosaurs* (Dinosauria: Hadrosauridae): Implications for Latest Cretaceous Megaherbivore Diversity in North America. *PLOS ONE* **6**, e25186 (2011).

152. Y. L. Bolotsky, P. Godefroit, A new hadrosaurine dinosaur from the Late Cretaceous of Far Eastern Russia. *J. Vertebr. Paleontol.* **24**, 351–365 (2004).

153. J. Yan, J.-F. Chen, T.-S. Gao, K. A. Foland, X. D. Zhang, M.-W. Liu, Studies on petrology and geochemistry of the Late Cretaceous basalts and mantle-derived xenoliths from eastern Shandong. *Acta Petrol. Sin.* **21**, 99–112 (2005).

154. Y.-Q. Liu, H.-W. Kuang, N. Peng, H. Xu, Y.-X. Liu, Sedimentary facies of dinosaur trackways and bonebeds in the Cretaceous Jiaolai Basin of Shandong Province and their paleogeographical implications. *Earth Sci. Front.* **18**, 9–24 (2011).

155. A. Prieto-Márquez, F. M. Dalla Vecchia, R. Gaete, À. Galobart, Diversity, relationships, and biogeography of the lambeosaurine dinosaurs from the European Archipelago, with description of the new aralosaurin *Canardia garonnensis*. *PLOS ONE* **8**, e69835 (2013).

156. D. Suzuki, D. B. Weishampel, N. Minoura, *Nipponosaurus sachalinensis* (Dinosauria; Ornithopoda): Anatomy and Systematic Position within Hadrosauridae. *J. Vertebr. Paleontol.* **24**, 145–164 (2004).

157. P. Cruzado-Caballero, X. Pereda-Suberbiola, J. I. Ruiz-Omeñaca, *Blasisaurus canudoi* gen. et sp. nov., a new lambeosaurine dinosaur (Hadrosauridae) from the Latest Cretaceous of Arén (Huesca, Spain). *Can. J. Earth Sci.* **47**, 1507–1517 (2010).

158. A. Prieto-Márquez, J. R. Wagner, The 'Unicorn' dinosaur that wasn't: A new reconstruction of the crest of *Tsintaosaurus* and the early evolution of the Lambeosaurine Crest and rostrum. *PLOS ONE* **8**, e82268 (2013).

159. A. Averianov, L. Nessov, A new Cretaceous mammal from the Campanian of Kazakhstan. *Neues Jahrb. Geol. Paläont. Monatsh.* **1995**, 65–74 (1995).

160. T. A. Gates, D. C. Evans, J. J. W. Sertich, Description and rediagnosis of the crested hadrosaurid (Ornithopoda) dinosaur *Parasaurolophus cyrtocristatus* on the basis of new cranial remains. *PeerJ* **9**, e10669 (2021).

161. P. Godefroit, S. Zan, L. Jin, *Charonosaurus jiayinensis* n.g., n.sp., a lambeosaurine dinosaur from the Late Maastrichtian of northeastern China. *C. R. Acad. Sci. Ser. II* **330**, 875–882 (2000).

162. P. Cruzado-Caballero, J. I. Canudo, M. Moreno-Azanza, J. I. Ruiz-Omeñaca, New material and phylogenetic position *of Arenysaurus ardevoli*, a Lambeosaurine Dinosaur from the Late Maastrichtian of Arén (Northern Spain). *J. Vertebr. Paleontol.* **33**, 1367–1384 (2013).

163. P. Godefroit, Y. L. Bolotsky, I. Y. Bolotsky, Osteology and relationships of *Olorotitan arharensis*, A hollow-crested Hadrosaurid Dinosaur from the Latest Cretaceous of Far Eastern Russia. *Acta Palaeontol. Pol.* **57**, 527–560 (2012).

164. P. Godefroit, Y. L. Bolotsky, V. J. Itterbeeck, The lambeosaurine dinosaur *Amurosaurus riabinini,* from the Maastrichtian of Far Eastern Russia. *Acta Palaeontol. Pol.* **49**, 585–618 (2004).

165. B. R. Peecook, J. A. Wilson, R. Hernández-Rivera, M. Montellano-Ballesteros, G. P. Wilson, First tyrannosaurid remains from the Upper Cretaceous “El Gallo” formation of Baja California, Mexico. *Acta Palaeontol. Pol.* **59**, 71–81 (2014).

166. A. Prieto-Márquez, L. M. Chiappe, S. H. Joshi, The Lambeosaurine Dinosaur *Magnapaulia laticaudus* from the Late Cretaceous of Baja California, Northwestern Mexico. *PLOS ONE* **7**, e38207 (2012).

167. M. A. Loewen, S. D. Sampson, E. K. Lund, A. A. Farke, M. C. Aguillón-Martínez, C. A. de Leon, R. A. Rodríguez-de la Rosa, M. A. Getty, D. A. Eberth, Horned dinosaurs (Ornithischia: Ceratopsidae) from the Upper Cretaceous (Campanian) Cerro del Pueblo Formation, Coahuila, Mexico, in *New Perspectives on Horned Dinosaurs: The Royal Tyrrell Museum Ceratopsian Symposium*, M. J. Ryan, B. J. Chinnery, D. A. Eberth, Eds. (Indiana Univ. Press, Bloomington, 2010), pp. 99–116.
